# Emerging cancer therapies: targeting physiological networks and cellular bioelectrical differences with non-thermal systemic electromagnetic fields in the human body – a comprehensive review

**DOI:** 10.3389/fnetp.2024.1483401

**Published:** 2024-12-10

**Authors:** Frederico P. Costa, Bertram Wiedenmann, Eckehard Schöll, Jack Tuszynski

**Affiliations:** ^1^ Oncology Department, Hospital Sírio Libanês, São Paulo, Brazil; ^2^ Department of Hepatology and Gastroenterology, Berlin, Germany; ^3^ Institut für Theoretische Physik, Technische Universität Berlin, Berlin, Germany; ^4^ Department of Physics, University of Alberta, Edmonton, AB, Canada; ^5^ Dipartimento di Ingegneria Meccanica e Aerospaziale (DIMEAS), Politecnico di Torino, Turin, Italy; ^6^ Department of Data Science and Engineering, The Silesian University of Technology, Gliwice, Poland

**Keywords:** network physiology, non-thermal, electromagnetic fields, radiofrequency, cancer treatment, cancer cells, oscillations, resonance

## Abstract

A steadily increasing number of publications support the concept of physiological networks, and how cellular bioelectrical properties drive cell proliferation and cell synchronization. All cells, especially cancer cells, are known to possess characteristic electrical properties critical for physiological behavior, with major differences between normal and cancer cell counterparts. This opportunity can be explored as a novel treatment modality in Oncology. Cancer cells exhibit autonomous oscillations, deviating from normal rhythms. In this context, a shift from a static view of cellular processes is required for a better understanding of the dynamic connections between cellular metabolism, gene expression, cell signaling and membrane polarization as states in constant flux in realistic human models. In oncology, radiofrequency electromagnetic fields have produced sustained responses and improved quality of life in cancer patients with minimal side effects. This review aims to show how non-thermal systemic radiofrequency electromagnetic fields leads to promising therapeutic responses at cellular and tissue levels in humans, supporting this newly emerging cancer treatment modality with early favorable clinical experience specifically in advanced cancer.

## 1 Introduction

The new field of network physiology uses the paradigm that in the human organism multicomponent physiological systems, each with its own regulatory mechanism, continuously interact to coordinate their functions in an integrated network (Ivanov 2021). It was first noted in 2012 that network physiology reveals relations between network topology and physiological function (Bashan et al., 2012). Coordinated network interactions among organs are essential to generating distinct physiological states and maintaining health. Physiological interactions occur at multiple levels of integration and across spatiotemporal scales to optimize organ functions and synchronize their dynamics at the organism level. These interactions are mediated by various signalling pathways that work in parallel to facilitate stochastic and nonlinear feedbacks (Ivanov et al., 1998; Hausdorff et al., 2001) across scales leading to different coupling forms (Bartsch and Ivanov 2014).

Recent publications have emphasized physiological networks and the pivotal role of cellular bioelectrical properties in driving cell proliferation, and cell synchronization with implications for cancer therapy. Innovative treatments based on non-thermal radiofrequency electromagnetic fields (EMF) have been developed, exploiting the unique electrical nature of all cells, particularly cancer cells, that exhibit autonomous oscillations distinct from healthy counterparts. This necessitates an understanding of the dynamic connections between cellular cybernetics, encompassing various processes like gene expression, cell signaling, and membrane polarization in human models.

This review illustrates the potential of targeting cellular dynamics using EMF as an emerging cancer treatment by exploring various aspects of EMF. We will begin by discussing how EMF, generated by biological processes, influence systemic network organization in the human body. We will provide examples of biological interactions and regulatory mechanisms modulated by EMF. Next, we explore the physical aspects of EMF within biological systems, covering topics such as signal propagation, interference in dynamic systems, and the electrophysiology associated with EMF. Additionally, we introduce several applications, such as pulsed or alternating electric, magnetic, and electromagnetic fields currently used in Medicine with a focus on locoregional targets, in contrast to EMF that provide a novel systemic oncology therapeutics. Finally, we discuss the direct effects of EMF at tissue, cellular and subcellular structures, presenting a comprehensive body of evidence from clinical and translational research. We end this review introducing EMF generator medical devices currently in clinical development, offering a future direction view for this promising novel field of oncology therapeutics.

### 1.1 EMF as the primary messenger in physiological networks

The human organism operates in a systemic network integrating organ systems, individual organs, cells, and biomolecules, all interacting on various levels in a physiological network. This framework of networks emphasizes dynamic interactions between the multitude of elements, resulting in an overall comprising, collective behavior not existing in isolated systems (Schöll, 2022). These interactions are nonlinear and adaptive, encompassing biophysical and biochemical control mechanisms, as well as communication and information exchange between cells and organs. These interactions determine the phenotype, which refers to the observable physical properties of an organism (Ivanov 2021; Nijhout and Reed, 2014). The given properties can be anatomical, biochemical, physiological, or behavioral traits that are evident in an organism (Nijhout and Reed, 2014). The phenotype results from the interaction of its genetic makeup (genotype) with the environment. These characteristics denote an evolutionary advantage for complex organisms facing constant genetic and environmental variations. However, disruptions in the homeostatic regime, caused by genetic perturbations or environmental shifts, can increase both phenotypic variation and the correlation between genetic and phenotypic variations. Robustness and stability are inherently imperfect and are maintained through dynamic processes, making them susceptible to disturbances from both genetic and environmental sources (Nijhout and Reed, 2014; Sawicki et al., 2022). The balance between stability and change is delicate, and by unleashing genetic variation, it may significantly facilitate rapid phenotypic evolution. This complex framework is crucial in both normal and pathological physiological states (Sawicki et al., 2022; Ivanov 2021).

The human body displays a vast array of rhythms and oscillations, including circadian rhythms, cell cycles, and hormone secretion. These omnipresent oscillators (systems that exhibit oscillatory, or repetitive, back-and-forth motion) frequently interact within tissues, organs, and cells, giving rise to complex behaviors that range from synchronization to chaos (Heltberg et al., 2021). The interaction of different oscillators is the physical mechanism known for generating a variety of dynamics. It is essential to understand that biological systems utilize these intricate dynamics, and these phenomena exhibit universal characteristics across different biological scales, from cellular to protein levels, which may explain the biological system’s ability to adapt and evolve in the face of both internal and external pressures (Heltberg et al., 2021). Recent studies on dynamical physiological networks have illuminated the range of collective phenomena, notably synchronization, a widespread characteristic in networks of coupled nonlinear oscillators (Schöll, 2021). The patterns of synchronization observed are diverse: cluster synchronization, for example, sees the network segmented into groups of synchronized elements (Dahms et al., 2012), while partial synchronization, such as chimera states, features a mix of coherent (synchronized) and incoherent (desynchronized) states existing simultaneously (Schöll, 2022; Berner et al., 2022). The concept of synchronization is of universal importance in natural and technological systems (Rosenblum et al., 2001).

We believe that the study of dynamics in supporting life’s complexity is a significantly overlooked area in science. When external (environmental) and internal systems show oscillations, the resulting dynamics depend on two main factors: the frequency ratio, the natural frequency relationship between the oscillators when they are not interacting, and the interaction strength, which describes how much an oscillator is influenced by the external cycle, often related to the amplitude of that cycle (Heltberg et al., 2021). This feature generally holds in physiological networks, and it has been in particular investigated for cortical input in brain dynamics (Sawicki and Schöll, 2024). The network dynamics of an entire organism cannot be accurately described just by adding up the behaviors of its individual systems. These dynamics can be significantly affected by small changes in the behavior of one system or by changes in how strongly it interacts with other systems, even if the overall network structure does not change (Uthamacumaran and Zenil, 2022). Experiments with microelectrode arrays provide insights about the compromise between spatial and temporal resolution for neuronal physiological networks, which allow extracellular voltage recordings of neural populations, as an example (Ballini et al., 2014; Emmenegger et al., 2019). Considering that neural oscillations involve very low energy (synaptic interactions), interaction strength of coupled oscillators would be mainly determined by spatial proximity and site of stimulation. However, microscopic oscillations result in the generation of field superpositions on a mesoscopic scale that also superpose with all the other produced oscillators and become detectable at the macroscopic scale. For this reason, we hypothesize that the frequency of oscillation (fundamental frequencies) is the fundamental component for understanding the patterns of synchronization, resonance, and chaos.

Current approaches in the single-cell molecular profiling of cancer cells are based on static, single-time point analyses. In time series, the molecular networks driving cancer processes may exhibit complex dynamics (Uthamacumaran, 2021). Modeling of the state space of cancer signaling and detection of partial synchronization patterns and desynchronization may have relevant clinical implications for cancer treatment. We believe normal and cancer networks behave as the classical paradigmatic Van der Pol oscillator (a nonlinear oscillator with a time-varying damping) that exhibits chaotic switching between two types of regular motion, namely periodic and quasiperiodic oscillations in the principal resonance region under exposure to EMF (Kadji et al., 2007; Van der Pol et al., 1927). For this reason, we postulate that EMF operate as the primary messenger for physiological and pathological network behavior. This review will discuss the scientific evidence supporting this statement.

### 1.2 Endogenous EMF

The human body generates numerous EMF (also called bioelectromagnetic fields) through its biological processes as a result of the electrical activities of the cells, tissues, and organs (Young, Hunt, and Ericson, 2021; Abdul Kadir, Stacey, and Barrett-Jolley, 2018; Kaestner et al., 2018; Tuszynski, 2019). These diverse EMF are present on different spatio-temporal scales, and they are superpositions of electric (E) and magnetic (B) vector fields (Robert, 2022). The voltage-activated ion channels produce relatively small EMF in the cellular membrane, which itself supports a large electric field across its thickness (Kaestner et al., 2018). In the central nervous system, for instance, a stratified hierarchy of endogenous EMF permeates the entirety of human physiology, extending from the cerebral cortex through deep brain structures and into an extensive extracerebral network distributed throughout the body. This organization of EMF is postulated to orchestrate and modulate neural activities in peripheral neural assemblies and other tissues, including those in the stomach and heart, synchronizing them with the cerebral rhythmic patterns (Hales, 2014).

The heart and the brain are the two most important organs generating EMF, with the EMF of the heart being several orders of magnitude higher than that of the brain (Fujiwara et al., 2018). It is well known that their electrical activity and variability is monitored by the electrocardiogram (ECG) and the electroencephalogram (EEG), respectively. This activity stems from the dynamics of ion channels in the plasma membrane. The cardiac myocytes, the sinoatrial pacemaker cells, and neurons are all excitable cells. However, other body tissues, including muscle, bone, and all non-excitable cells, also interact and generate EMF, though with much lower intensity (Hales, 2014; Suzuki et al., 2020; Vaiciuleviciute et al., 2021; Langthaler et al., 2022). EMF signals generated by non-excitable cells are challenging to measure and those EMF signals can be represented through models that consider the random and unpredictable behavior of ion channels. For instance, the dynamic, non-linear oscillations of subcellular calcium, that act as a mechanism for detecting extremely weak signals in biological systems, exemplify this approach (Shen and Larter, 1995; Sandblom and Galvanovskis, 2000). Excitable and non-excitable are two categories of cells that differ in their ability to generate and conduct electrical signals. The former can generate action potentials that are transmitted over longer distances (up to 1 m). The latter cannot transmit electric signals, but instead use biochemical signals to communicate. Both categories of cell types are interconnected to conduct synchronized activities throughout the body by chemical and electric as well as contact interactions (Ghomsi et al., 2015). The physical basis of EMF interactions is based on non-linear behavior (Uthamacumaran, 2021), and it is well explained by the macroscopic form of Maxwell’s equations (Robert, 2022). The human organism is a multi-component physiological system with its own regulatory mechanisms that continuously interact to coordinate their functions in an integrated complex network (Ivanov 2021). On a micro-scale, the endogenous EMF are relatively weak, but they have an intricate fine structure, where a system of field sources is spatially extended. The interaction (e.g., coupling) of the different sources of endogenous EMF is represented by a complex system characterized as non-linear, non-homogenous, anisotropic, non-stationary, and far-from-thermodynamic equilibrium (Hales, 2014). The totality of the generated EMF permeates the tissue, including all intracellular and extracellular spaces. As a result, in a living organism under normal physiological conditions, those fields exist as a seamless unity regardless of their sources. In the case of pathologies, this highly dynamically ordered state of biological matter may undergo a breakdown of global synchronization. This is a very important paradigm of our network approach.

### 1.3 EMF biological interaction

Low-Energy EMF can have non-ionizing interactions with biological systems, leading to thermal and non-thermal effects, depending on the exposure’s frequency, intensity, and duration. Those interactions as described by a variety of physical mechanisms, leading to potential epigenetic alterations. Thermal effects occur when EMF energy increases the biological system’s temperature, analogously to heated vibrating/bubbling water. Thermal effects are mostly associated with higher frequency EMFs, particularly those in the microwave range (300 MHz–300 GHz) and the upper radio frequency range due to their high rate of absorption by biological systems. Infrared radiation (IR), just below the visible light frequency range (around 300 GHz to 400 THz), can also produce thermal effects, primarily heating the skin and the superficial tissues. The corresponding electrical field intensity is also essential for thermal effects, measured in Volts per meter (V/m). For devices close to the body, such as mobile phones, the Specific Absorption Rate (SAR) is also used to measure intensity in Watts per kilogram (W/kg). Regulatory bodies set SAR limits to ensure devices do not cause significant thermal effects. For example, the USA Federal Communications Commission (FCC) has set a SAR limit of 1.6 W/kg averaging over 1 g of tissue for the head and torso (https://www.fcc.gov/) representing low-energy EMF. Non-thermal effects are not related to the heating of tissues but involve other mechanisms at the cellular or molecular level (Wust, Kortüm, et al., 2020). These may include, for instance, changes in cell membrane permeability, ions flux, alterations in enzyme activities, changes in cellular growth patterns, changes in protein dynamics, and impacts on nerve cell signaling. Non-thermal effects are primarily associated with a low-frequency range (LF: 30 kHz–300 kHz), very low-frequency (VLF: 300 Hz to 30 kHz), and extremely low-frequency range (ELF: 0 Hz–300 Hz). The latter is in the range of most biological oscillations intrinsic to the human body.

Interest in the electrical characteristics of key cellular components such as microtubules, actin filaments, DNA, and ion channels has led to notable discoveries, particularly regarding how these elements respond to alternating current (AC) (e.g., alternating oscillation) in terms of electrical conductivity (e.g., a measure of a material’s ability to conduct an electric current) (Tuszynski, 2019). Since cells are mostly composed of water, which accounts for 70% of their mass, water’s role in conducting electrical signals is pivotal. Electrical conduction through proteins and DNA involves not just electrons and protons but also various ions in the cell’s cytoplasm. The cellular environment is rich in ions such as potassium, sodium, chloride, magnesium, and calcium, along with protons that contribute to the cell’s natural ionic conductivity, influenced by the cell membrane’s potential and ion channel activity. These ions may move freely within the cytoplasm or along electric field lines, often following paths defined by the cell’s polymeric structures, which is significant for understanding how ionic concentrations and oscillation frequencies affect conductivity in living cells (Freire, Bernal-Méndez, and Pérez, 2020a).

**FIGURE 1:**
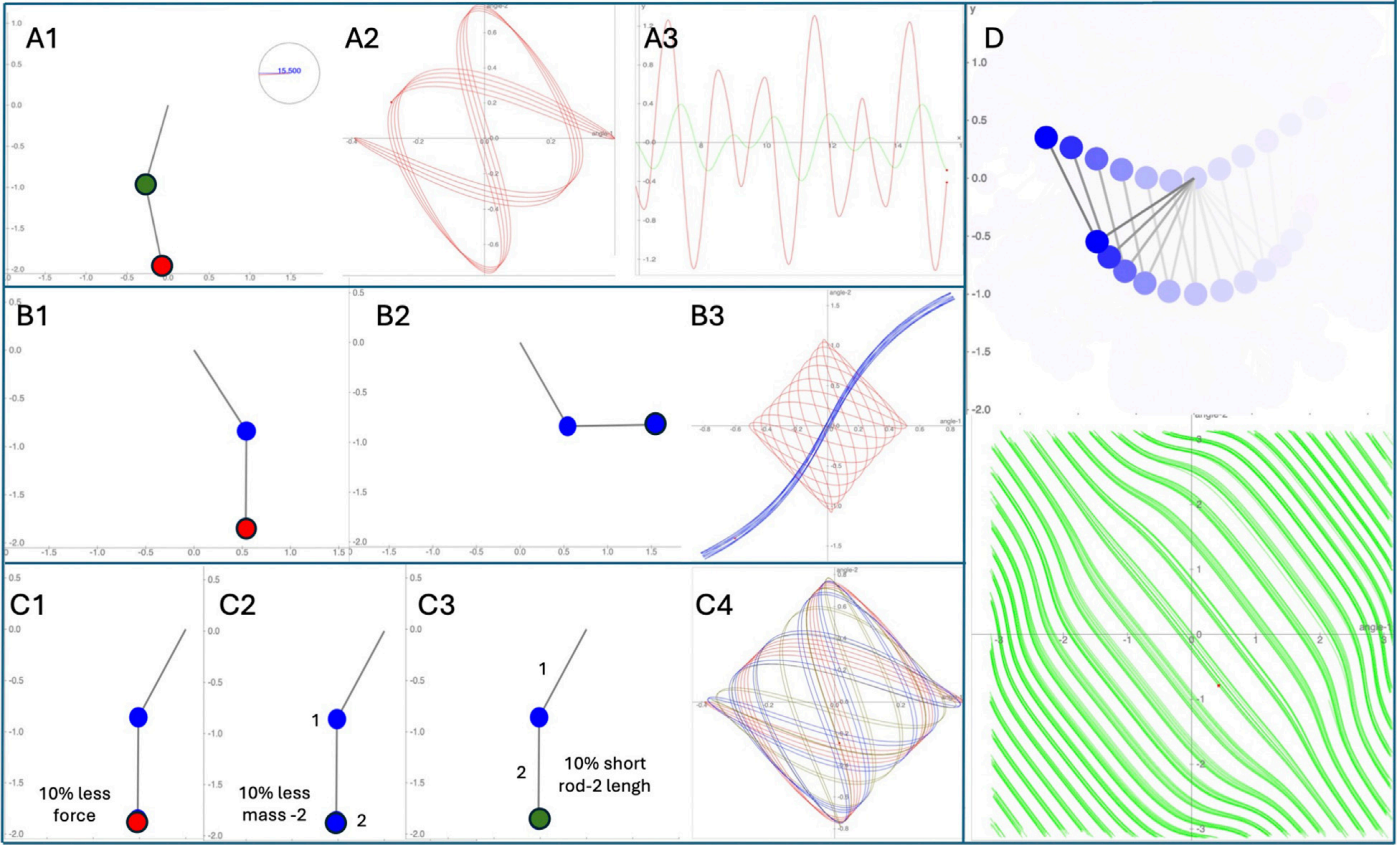
Schematic description of double pendulum simulations. **(A1)**: double pendulum at 15 s simulation, **(A2)**: Lower pendule phase-time motion represented by sequential small divergent trajectories, **(A3)**: Time graphic showed two coupled oscillations (upper rod – green, lower rod – red) with aperiodic amplitude variations. **(B1, B2)** represented two identical double pendulums at different initial conditions. **(B3)**: Phase-time motion represented two different trajectories (red – B1, blue – B2). **(C1–C3)** represented three double pendulums with only one variable difference (10%) among the others. **(C4)**: Phase-time motion at 15 s simulation for **(C1–C3)** with same initial condition showed three different trajectories. **(D)**: In motion, double pendulum showed chaotic behavior (green).

The cell membrane has been demonstrated to exhibit complex impedance-like behavior during rapid ionic flux changes, providing resistance to variations in ionic current through its channels or pumps. It comprises a double phospholipid layer, separating external ions from those within the cytoplasm and charged proteins. Although pure lipid layers are excellent insulators, the actual biological membrane is a complex mix of proteins and lipids, with many proteins acting as channels to facilitate charge movement, effectively reducing the membrane’s resistance, which is crucial for electrophysiological studies (Bezanilla, 2008). Additionally, the membrane’s ability to separate opposite charges endows it with capacitance, enabling it to store and release electric energy. The membrane’s electrical properties are equivalent in-parallel RLC circuit (electrical circuit consisting of a resistor (R), an inductor (L), and a capacitor (C), connected in parallel). This arrangement means that when subjected to a sinusoidal electromagnetic force, the cell membrane can filter the input, producing currents at specific frequencies akin to how an RLC circuit operates (Der Pol et al., 1927; Kumai, 2017) (Figure 1). RLC circuits exhibit resonant behavior when subjected to externally applied voltage oscillations. Hence, they naturally vibrate or oscillate, like a child on a swing when pushed periodically at the right rate. Natural oscillations occur when a restoring force pulls the system back to its equilibrium position when displaced, and there is inertia that causes the system to overshoot the equilibrium. To observe natural oscillations in an RLC circuit, an external oscillating force is needed to displace the system from its equilibrium position and let it go. If an oscillating force is applied to a system that already oscillates naturally at matching harmonic frequencies, the system will enter in resonance, where the oscillations progressively grow to a very large amplitude corresponding to the highest rate of energy transfer.

### 1.4 EMF biological regulation

Many systems within the human body are under neural regulation in physiological conditions (West, Brown, and Enquist, 1997). Adaptation to dynamic physiological states occurs under strictly controlled conditions (Ivanov et al., 1996). A model framework of complex networks has been developed that supports the concept of dynamic interactions (coupling) between physiological systems (Bartsch et al., 2015). These interconnected systems undergo transitions from one physiological state to another on different timescales (Hales, 2014). Bioelectromagnetic fields or endogenous EMF are involved in the synchronization of regulatory processes by sending clear “signals” arising from specific coherent atomic/molecular/cell/tissue/organ-level sources (Hales, 2014). This synchronized steady state is known as the “electromagnetic homeostasis” (De Ninno and Pregnolato, 2017). This concept has been accepted by a number of researchers, however, the understanding of the complexity of those physiological network relations expressing non-linear behaviors and their topology is still a challenge (Bartsch et al., 2015; Uthamacumaran, 2021; Sun et al., 2022; Scholkmann, 2015; Limansky et al., 2014). One example of network physiology regulation on different time scales is the cross-frequency coupling of the brain (Canolty and Knight, 2010).

Neural plasticity manifests across various organizational levels and temporal scales, adapting to diverse inputs and producing assorted output dynamics. At the microscopic scale, areas of the brain, defined by a specific frequency band (e.g., alpha 11–12 Hz, sigma 12–16 Hz, beta 16–30 Hz, gamma 25–140 Hz, theta 4–7 Hz and delta 0.5–4 Hz), can interact with other areas of the brain with different frequency bands through entanglement (von Stein and Sarnthein 2000). The coupling between areas with low-frequency phase and high-frequency amplitude has been observed in cognitive processes of decision making, motivation and memory, as an example (Schroeder and Lakatos 2009). At the macroscopic scale, neural plasticity and neuromodulation play pivotal roles in modifying brain excitability and managing physiological states such as sleep and arousal. This dynamic communication within the brain’s network is evident not only in the synchronized activity within identical frequency bands across different brain regions but also through the interactions between distinct frequency bands. These interactions, traditionally linked with specific physiological states and functions, serve as markers of such states. The network’s dynamics regulate the collective behavior of brain rhythms, with network topology and the strength of connections undergoing hierarchical reorganization during transitions between states. These findings underscore the significance of brain-rhythm interactions in the generation of physiological states and cognition, highlighting key contributions in the field (Liu et al., 2015; Lin et al., 2020; Chen B. et al., 2022).

The human body is composed of some 37 trillion living cells, and it is a thermodynamically open system that utilizes metabolic energy to maintain its functions. The organism should be kept at an elevated level of efficiency and stability even under constant environmental stress, which requires the establishment of coherent energy transfer dynamics (Olaya-Castro, Nazir, and Fleming, 2012). This state is known as “metabolic homeostasis” (Wilson and Matschinsky, 2021). Conversely, lack of homeostasis, through allostatic overload due to stress or disease, may lead to a transition to pathological states of human physiology (McEwen and Wingfield, 2010). It is well recognized that sequential physiological abnormalities in patients lead to multiple organ dysfunction syndromes under critical conditions that can still be reversible in response to intensive care procedures (Asim, Amin, and El-Menyar, 2020), thereby representing a progressive network failure of uncoupling/recoupling processes involving physiological “oscillators” (Buchman, 1996). This is completely in line with the new paradigm of a “network disease,” suggested for instance for epilepsies (Lehnertz et al., 2013; Gerster et al., 2020). This new framework acknowledges that human diseases stem not from isolated molecular faults but from intricate interactions among numerous molecular agents (Conte et al., 2019). The loss of physiological variability implies systemic isolation by uncoupling between systems and their stochastic processes (Pincus, 1994). The direct estimate of physiological system coupling is associated with measurements of variability (Force et al., 1996). Heart rate variability (HRV) is a well-accepted surrogate marker for physiological variability and organism-wide stability (D'Angelo et al., 2023; Tiwari et al., 2021). HRV describes the variations between consecutive heartbeats, known as R–R intervals (RRI) over a time interval (Force et al., 1996).

## 2 Physical characteristics of EMF in biological systems

### 2.1 Stress response

The human body behaves like any heterogeneous media (e.g., matter) in the path of EMF wave (Nath et al., 2021). EMF exposure can be perceived by the human body as an environmental stress that can produce causal effects in human tissues by complex interactions between EMF and electrostatically charged components of the biological system (Kivrak et al., 2017). In general, resonance occurs when a physical or biological system is exposed to an external force (e.g., EMF) that oscillates with the system’s natural frequency of oscillation. However, in non-linear systems the resonant frequency range broadens (and can comprise multiple resonance frequencies) and there is a relationship between different oscillations known as cross-frequency coupling (phase-locking zones or Arnold tongues) (Gérard and Goldbeter, 2012; Glass, 2001), as it is well exemplified by the brain dynamics or even the cell cycle. Resonance oscillatory systems are coherent, described by the numerical ratios between them, and act as an information processing system (Oizumi, Albantakis, and Tononi, 2014; Hunt and Schooler, 2019; Rassi et al., 2019). External periodic stimulations (e.g., sinusoidal EMF wave) produce periodic synchronized rhythms and aperiodic rhythms inducing quasiperiodic rhythms, where different frequencies have some level of interaction strength (e.g., chaotic dynamics) (Glass, 2001; Uthamacumaran, 2021). Physiological oscillations can be synchronized by appropriate external stimuli, and the effects on the physiological oscillations can then be analyzed (Glass, 2001). Physiological rhythms can be stimulated by regular, periodic inputs that occur in the context of medical devices (e.g., heart pacemakers) or natural variations in the ambient conditions (e.g. circadian rhythms). Considering the chaotic dynamics of physiological oscillations, well-timed interaction (e.g., sinusoidal EMF wave) can produce strong effects on the dynamics of the human body (Shinbrot, 1995). These frequency- and phase-synchronization phenomena (alignment of oscillations in terms of their frequency and phase) are well-known in the physics of nonlinear dynamical systems where an external force with a particular frequency can cause coupled initially desynchronized oscillators to become synchronized over time, which has found numerous applications in biology and physiology (Jiménez et al., 2022). We believe that this mechanism is eminently applicable to the effects of EMF on cancer cells. This example can be likened to a maestro leading an orchestra. Initially, the individual instruments emit random noises, but when guided by the maestro’s precise movements at a specific frequency, they harmonize to create a beautiful sonata.

EMF exposure (produced by an external oscillator) acts as an external stimulus that interacts with the internal oscillator (e.g., human body) where resonant frequencies lead to the most efficient energy transfer between the two systems (e.g., analogous to a parent pushing a child on a swing) (Robert, 2022). Energy transfer can be efficient at frequencies that are subharmonics (fractions of the fundamental frequency) or harmonics (integer multiples of the fundamental frequency) of the internal oscillator (Uthamacumaran, 2021). The synchronization and entrainment of oscillators is known as frequency-locking, or phase-locking, and allows the oscillators to coordinate their activities (Heltberg et al., 2021). The emergent synchronization patterns are based on the initial properties of the oscillators and their coupling strengths (Uthamacumaran and Zenil, 2022; Jensen and Krishna, 2012; Mackey and Glass, 1977).

Synchronization patterns are the accepted mechanism of the direct EMF effect on excitable cells that propagate to the tissues. EMF affect action potential signaling by direct coupling to become a second signaling system, routinely reported as a “closed loop” behavior (Anastassiou et al., 2010). EMF represent the “regions of space” where electric and magnetic fields interact with charged particles (via Coulomb and Lorentz forces) that mediate bioelectrical attributes of cells, such as the membrane potential (Accardi, 2015; Zhou et al., 2015; Freire, Bernal-Méndez, and Pérez, 2020b). For example, ionic currents flow into and out of a neuron, causing changes in the extracellular space (ECS) potential that can then influence the excitability of other nearby neurons close by. This type of signaling is known as ephaptic signaling, now considered relevant for neuronal synchronization (Han et al., 2018). Moreover, the ECS gap occupies approximately one-fifth of the brain’s volume, structured in a web filled with interstitial fluid. The ECS can be viewed as a container of ions used for electrical activity (Hrabetova et al., 2018). ECS occupies a narrow space in the synapse and produces EMF (Hales, 2014). The intracellular space (ICS) and ECS are separated by a lipid bilayer membrane that possesses specific dielectric properties (material that is a poor conductor of electricity), dependent on its biochemical composition and EMF frequency (Dilger et al., 1979). Again, coherence is the fundamental element for the dynamic formation and interaction of EMF. The ion motion between those spaces through channels that are synchronized in position, direction and time produce synchronized currents. The resultant EMF are also coherent, and they influence the nearby space interacting with all other dipole moments (measure of the separation of positive and negative electrical charges within a system) and moving electric charges. Many ion channels in particular locations produce polarization in other regions of adjacent ECS and ICS, with space-charge equilibration effects. Across the ion channels, ionic currents are present on the microscopic scale, with EMF superposed in space. One example of this phenomena is the neuron firing action potentials. The resultant EMF generated during this process are coherent and extend into the surroundings interacting with nearby dipoles, electric charges and creating localized polarization effects. This polarization spreads, affecting neighboring regions through space-charge equilibration, thereby influencing the overall electrical activity of the neural network. It is interesting to note that such synchronizing effects of cortical input depending upon the frequency and location of the input has been found in simulations and although this was investigated with the aim to understand sound input into the auditory cortex, this seems to be far more generally applicable to electrical input into various brain regions (Sawicki and Schöll, 2024). Under specific conditions, a polarization effect occurring in the regional ECS/ICS can lead to the generation of field superpositions on a mesoscopic scale. These EMF also superpose with all the other produced fields and become detectable at the macroscopic scale (e.g., organ level) (Hales, 2014). This polarization is important to cell membrane potential and voltage-gated activity (Bonzanni et al., 2020). A relationship between cellular proliferation and a cellular electrical potential (e.g., membrane potential) has been long recognized and studied (Binggeli and Weinstein, 1986; Wonderlin and Strobl, 1996; Cone, 1971; Cone and Tongier, 1973; Rao et al., 2015).

### 2.2 Signal propagation

Electromagnetic waves travel in the direction perpendicular to the plane in which the electric field E and magnetic field B vectors oscillate over time. Electromagnetic wave propagation follows Maxwell’s equations and has a velocity equal to the speed of light in vacuum (Robert, 2022). The oscillating electric fields (EF) “accelerate” charged particles in alternating directions. The magnetic fields “align” magnetic moment possessing molecules (magnetic dipoles or spins) like a compass needle that aligns with the Earth’s magnetic fields. Magnetic fields also cause moving charges to rotate in so-called cyclotron orbits in the plane perpendicular to the magnetic field direction. EMF can propagate with and without a medium irrespective of their frequency. EMF waves are subjected to reflection, refraction, and diffraction. Light is a small slice of the electromagnetic spectrum, and we are all familiar with the described behavior because we can literally see it. Electromagnetic waves transmit energy through space, interacting with the material substrate based on wave frequency and intensity (Robert, 2022).

Radiofrequency (RF) waves are at the very low end of the EMF spectrum with their frequencies in the range of 3 Hz–3 THz. When RF is applied at an extremely low to moderate electric field intensity (<150 V/m), the risk of thermal damage is known to be minimal if detectable at all (Funk, Monsees, and Ozkucur, 2009). For both electric fields and electromagnetic waves, as they enter and propagate through matter, part of the energy is absorbed according to frequency-dependent characteristics of the material (e.g., dielectric properties). The energy loss produces storage of electrical energy (polarization), dissipation of charged particles and reorientation of dipoles (Panagopoulos, Johansson, and Carlo, 2015). Thus, electric fields and EMF energy propagates to all parts of the tissue including all intracellular and extracellular spaces delivering energy to the tissue. The dielectric and conductometric behavior of cells are shape dependent in a frequency range where the interfacial polarization characteristic of highly heterogeneous systems occurs (Di Biasio and Cametti, 2007). EMF produce electric fields in the tissue by induction (Ravin et al., 2020). However, EMF suffer energy loss or attenuation during propagation due to energy absorption, resulting in decrease in the amplitude of the signal. EMF propagation is different from alternating electric fields, which are generated by surface electrodes and electrons flowing (e.g., electrical current) between the opposite electrodes (anode and cathode) in an alternating fashion at specific frequencies whereby electrical currents move in the tissues preferentially through the extracellular space. The cells attenuate alternating electrical currents based on their high impedance. However, structural changes during cytokinesis result in an inhomogeneous intracellular electric field distribution, concentrated heavily at the cleavage furrow where cells divide. This non-uniform field triggers the emergence of dielectrophoretic forces acting on polar molecules in cells (Tuszynski et al., 2016).

EMF, in contrast to electric fields, can travel through space. For example, in radio broadcasting, the amplitude of a signal is altered to carry information, whereby all information is transmitted through electromagnetic waves. In electronic communication, for instance, amplitude modulation (AM) is essential as a primary method for EMF signal transmission. AM is carrying signals by changing the shape of the envelope for carrier frequency waves (Tuszynski and Costa, 2022). Importantly, AM signals may produce oscillatory perturbations that interact with biological rhythms at very low and even extremely low frequencies (Lewczuk et al., 2014). These interactions become more noticeable (e.g., recordable) when the target tissue emits rhythmic electrical signals from cells that naturally pulse at similar rates as the oscillations found in AM signals, which range from 1 to 100 Hz (Tuszynski and Costa, 2022; Bertagna et al., 2021; Sawicki and Schöll, 2024).

### 2.3 Dynamic response

Until now the concept of EMF interactions with cellular targets has remained relatively unfamiliar to most cancer researchers and oncologists (Gherardini et al., 2014). This interaction underscores the fundamental concept that all cells, especially cancer cells, exhibit distinct electrical properties that are crucial in determining their physiological behavior. An example of the interaction of EMF and biological oscillations is the synchronization of circadian rhythms (Patke, Young, and Axelrod, 2020; Schmal et al., 2022). A molecular foundation for circadian oscillations is evident at the cellular level where key elements underpinning these autonomous cellular oscillations include fluctuations in gene transcription, protein biosynthesis and ionic flow such as ion translocation, and ionic concentration within the cytoplasm. Ionic currents perpetuate the circadian alterations at the rate of action potential generation, thereby regulating the synchronization and functional dynamics of neuronal networks (Stangherlin, 2023). These properties are not static; they exhibit complex, nonlinear dynamics (e.g., complex systems such as weather patterns and stock markets) highly contingent upon the cells’ initial conditions. This means that the effect of EMF on cellular behavior can vary significantly based on the characteristics of the cell, their microenvironment, tissue location and organic homeostasis structure. Thus, it is understandable that normal and cancer cells would respond differently to the same EMF exposure since they are characterized by vastly different dielectric constants, electric conductivities and transmembrane potential gradients. The interaction between EMF and cellular bioelectrical systems is embedded within the body’s systemic framework (Freddolino and Tavazoie, 2012).

All living cells operate as open thermodynamic systems, which implies they participate in exchanging energy with their surrounding environment thereby encompassing intracellular pathways, biochemical and bioenergetic processes providing swift enablement of adaptation to alterations of the microenvironment. For example, to maintain energy efficiency, cells store the energy by the biosynthesis of Adenosine Triphosphate (ATP) through glycolysis or oxidative phosphorylation under anaerobic and aerobic conditions, respectively. Another example is the distribution of ion channels and transporters in the plasma membrane. These specific molecules enable epithelial cells to create ionic gradients within the cell, allowing them to regulate the transportation/traffic of various molecules such as sugars and amino acids. Epithelial cells achieve and uphold membrane protein polarity by employing sorting and trafficking mechanisms, ensuring that cargo is directed to either the apical or basolateral cell surface (Levic and Bagnat, 2022). These pathways exhibit a so-called oscillatory behavior, regulated by negative feedback mechanisms to optimize efficiency, and mitigate the production of reactive oxygen species (ROS). Such oscillations contribute to the evolutionary selection and stabilization of cellular structures, embodying an effective method for the temporal and spatial encoding and transmission of information (Cheong and Levchenko, 2010). Biological oscillatory systems are characterized by cyclic motions with repeatable intervals, where one complete cycle defines a period of oscillation. In periodic motions, frequency is defined as the number of oscillations per time unit. Oscillatory patterns are synchronized across cellular populations in response to diverse biochemical and metabolic activities which provide functional advantages over static signaling inputs. Thus, essential molecules present in biological tissue such as ions, water molecules, proteins, nucleic acids, and lipids, possess either a net electrical charge or a dipole moment, often in periodic motion, hence can be affected by EMF (Fenwick, Esteban-Martín, and Salvatella, 2011).

EMF induced synchronization is easier to demonstrate studying action potentials in excitable cells rather than in non-excitable cells. However, EMF induced synchronization of gene expression and metabolic pathways – though more challenging – can be also demonstrated in non-excitable cells (Del Olmo et al., 2023; Friesen, Baracos, and Tuszynski, 2015; Piszczek et al., 2022; Moraveji et al., 2016; Mousavi Maleki et al., 2022; Phillips, 1993; Zimmerman et al., 2012). When the external EMF is locked at specific frequencies, the internal system exhibits different amplitudes of oscillation in every cell, regardless of the cell type. The key characteristic of this interaction is that the period of oscillation remains constant, independent of the amplitude (e.g., constancy of period, regardless of the amplitude) and it has limited cycles of oscillations with a given amplitude and period (Robert, 2022). However, most biological oscillations have a mechanism involving a delayed negative feedback loop (see review (Baker and Rutter, 2023)). This process can be conceptualized as involving an activator, labeled “x,” which leads to the production of a protein, “y.” This protein then activates a transcriptional inhibitor, designated “z,” that represses the activity of the activator “x.” Depending on the heterogeneity and complexity of a negative feedback loop, when the external EMF is locked, it can result in a positive influence (e.g., speed up) with diversion dynamics of the oscillations or can produce a negative influence (e.g., slow down) with conversion dynamics of the oscillations (Del Olmo et al., 2023). This activator-inhibitor mechanism is well studied, in particular in excitable media where it can be described by a paradigmatic model known as the FitzHugh-Nagumo model (Lindner et al., 2004; Schöll 2020, Schöll, 2021). Delayed feedback loops, on the other hand, occur frequently in physiological and neuronal networks due to finite propagation times and signal processing times, and represent a versatile concept of control (Schöll et al., 2009; Schöll et al., 2016; Sawicki and Schöll, 2024; Erneux 2024; Rosenblum 2024).

Different cyclic-controlled elements exhibiting positive and negative feedback can show complex interactions with external EMF. Adjustments in the biological system in response to the external force can lead to substantial changes in its long-term dynamics (e.g., initial condition) resulting in intricate phenomena. Negative feedback loops play a crucial role in stabilizing regulatory systems, enabling them to reach their equilibrium state more efficiently (Ferrell, 2013). Cell cycle checkpoints can be likened to such negative feedback loops. For instance, during mitosis, the spindle assembly checkpoint is triggered upon detecting unoccupied kinetochores. This checkpoint halts the progression through mitosis, allowing the cell additional time to ensure proper kinetochore occupation. Consequently, the activation of a checkpoint due to unoccupied kinetochores initiates a feedback mechanism that reduces the number of unoccupied kinetochores - a classic example of a negative feedback loop (Ferrell, 2013).

This quality makes feedback loops valuable in maintaining homeostasis within the entire system. The prevalent control architecture in heterogeneous multiscale problems is hierarchical, where control at the macroscale is decomposed into nested control problems at decreasing mesoscales. Signals of different scales are modeled and controlled accordingly, with finer scales addressing small amplitude, high-frequency signals, and larger scales handling larger amplitude, lower frequency signals. This hierarchical structure allows for nested design of regulatory feedback loops, with outer loops regulating coarser scales and inner loops regulating finer scales. Thus, feedback regulation operates across scales without hierarchical constraints (Sepulchre, Drion, and Franci, 2019). It is recognizable that this scenario exhibits two or more stable oscillation states, known as multi-stability. This behavior expresses high variability with dynamical switching of oscillatory modes often observed in non-linear biological systems that become chaotic (Goldberger et al., 2001). Chaos refers to a well-defined mathematical concept that can emerge in deterministic dynamical systems. The concept of chaos is defined when two or more dynamical behaviors, separated initially by an arbitrarily small distance in their phase space, will exhibit exponentially diverging trajectories in phase space as time proceeds (Wang et al., 2023).

### 2.4 Systemic behavior

Understanding that due to external EMF the system dynamics can diverge significantly from its initial condition, it is important to consider both trajectories (initial and new). In other words, in the context of chaos theory, small changes in the initial conditions of a system can led to significantly different outcomes. This sensitivity to initial conditions is a hallmark of chaotic systems. Thus, the system dynamics can switch between different trajectories and the new state influenced by EMF overlaps or modifies the original state. As an example, imagine a dough being pressed, stretched, and folded while making pastry. The EMF is the work force applied in the dough also named “attractor” (e.g., a magnet that pulls the system’s behavior towards itself), and it is conceivable that distinct types of attractors and conditions coexist. The kneaded pastry started as a dough (e.g., initial condition) to become dispersed, leading to a loss of specific information about these initial conditions during its transformation into pastry. In special conditions, the attractors could be composed of fractal structures confined to a limited space. Those attractors can bring the system to chaotic dynamics (aperiodic and sensitively dependent upon initial conditions). Another example is the image of a coupled double pendulum, with the second pendulum hanging from the first one. When you set this system in motion, it exhibits chaotic behavior (Rafat, Wheatland, and Bedding, 2008). Despite having deterministic rules governing its motion (Newton’s laws of motion), the system’s behavior is highly sensitive to initial conditions. Even small differences in the starting positions or velocities of the pendulums can lead to vastly different trajectories over time. This sensitivity to initial conditions is a hallmark of chaotic systems. As the pendulums swing, they create intricate and seemingly random patterns, making their future behavior unpredictable beyond a certain point (Figure 1).

Another aspect of oscillatory systems with feedback loops is that they typically exhibit oscillations where the feedback acts to counter the system’s output (damping) (Kochen et al., 2022). Damped system dynamics often result from the inherent randomness in biochemical reactions, that disrupts phase coherence and dampens oscillations. Cancer cell behavior has been proposed to present damped EMF activities (Pokorný et al., 2020). Cancer often leads to the system expending more energy than it can replenish, resulting in a gradual reduction in oscillation amplitude and an increase in entropy (quantifies the degree of disorder or randomness in a system). Characteristic bioelectric behavior is one of the new accepted hallmarks of cancer and it is crucial for understanding how electrical signals and charges contribute to the onset, growth, and progression of cancer, and the emerging significance of bioelectricity in cancer proliferation and metastasis (Moreddu, 2024; Schwartz, Supuran, and Alfarouk, 2017; Hanahan and Weinberg, 2011). Cancer cells exhibit autonomous oscillations, deviating from normal rhythms observed in processes like glycolysis (a phenomenon known as the Warburg Effect whereby a shift occurs from the predominantly oxidative phosphorylation mode of energy production toward glycolysis) (Vander Heiden, Cantley, and Thompson, 2009; Rietman et al., 2013; Sawicki et al., 2022). This indicates a distinct signaling system in cancer cells compared to healthy ones. Furthermore, cancer can be conceptualized as exhibiting parasitic energy consumption, that leads to a dampening effect on the cellular electromagnetic field. A key aspect of this process is mitochondrial dysfunction in cancer cells (again, the Warburg effect), that significantly reduces respiration. This dysfunction reverses the membrane polarity of the structured water layers surrounding mitochondria, thereby dampening the electromagnetic activity of cells in the vicinity. Consequently, the oscillatory frequency and power of the electromagnetic field generated by these cells are altered. This alteration potentially disrupts communication between normal and cancer cells, leading to decreased control over chemical reactions and an increased likelihood of random genetic mutations (Pokorný et al., 2020; Kamińska et al., 2015).

**FIGURE 2:**
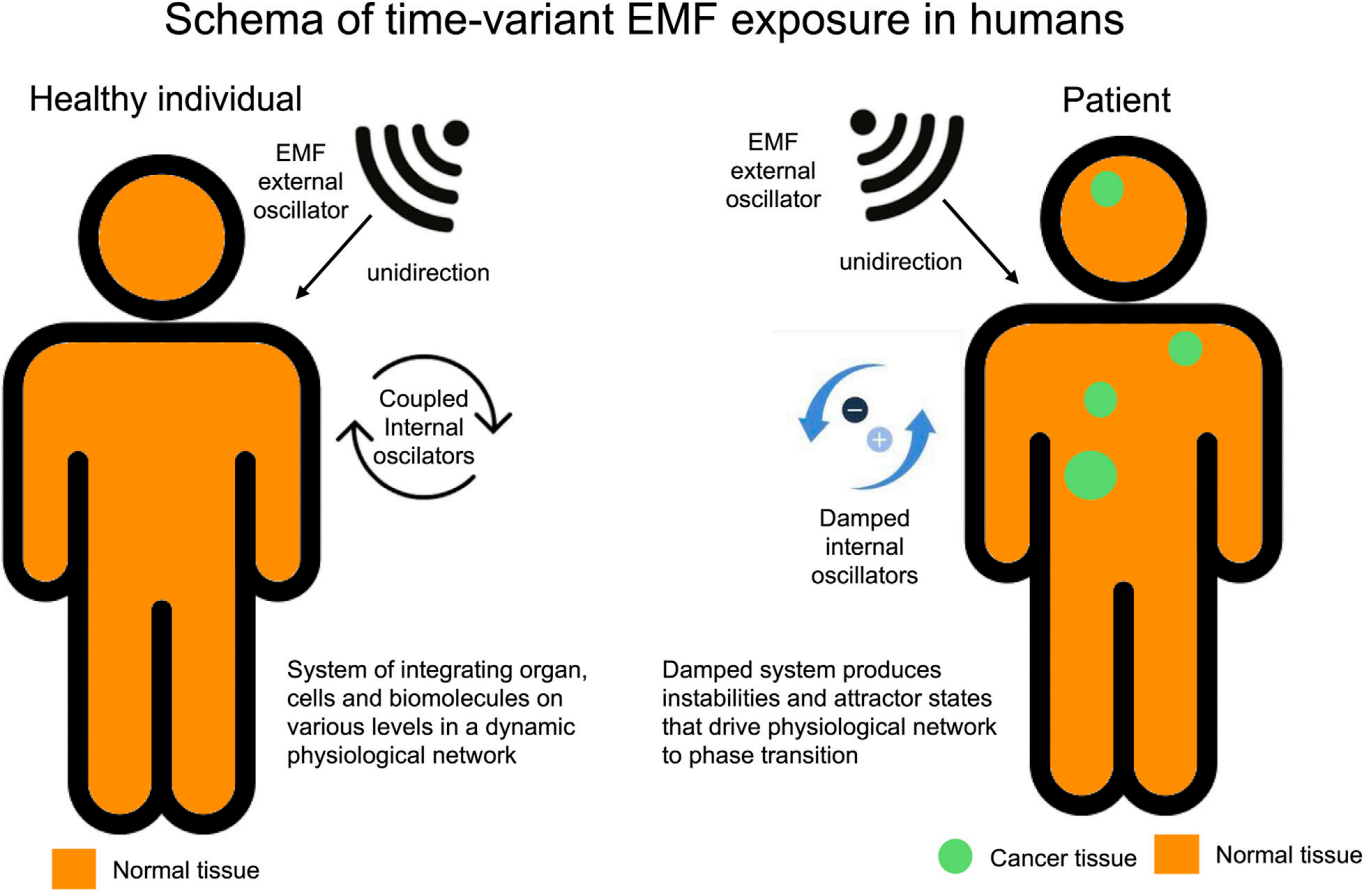
In healthy individuals, time-varying electromagnetic fields (EMF) do not affect systemic hemodynamics. This lack of effect is due to the complex and tightly integrated dynamics of a healthy system, which remains stable even when exposed to the low energy levels of EMF used, as these are too weak to cause any interference. However, in less coherent systems, there is an observed susceptibility to time-varying EMF. For example, in diseases like cancer, the normally orderly dynamics can become disrupted, losing their overall synchrony. Time-varying EMF, when applied at specific frequencies, is designed to help restore this synchrony, despite the disruptive influence of the cancer.

Cancer cells explore a more complex version of oscillator interaction, where both oscillators are internal and one oscillator interacts with the other and *vice versa* with progressive and disproportional damping forces driving the system to a phase transition to new states of dynamic instability (Davies, Demetrius, and Tuszynski, 2011). The new state of dynamic instability makes the system more sensitive to external force interference (e.g., EMF). One example is the dynamic instability of microtubules, that refers to the stochastic switching between growth and shrinkage phases exhibited by certain biological polymers. For example, microtubules can undergo dynamic instability, where they alternate between phases of growth (polymerization) and shrinkage (depolymerization). During dynamic instability, the system becomes more sensitive to external forces or perturbations. This sensitivity arises because the system is in a state of flux, with individual polymers undergoing rapid changes in length and stability. External forces can influence the direction and rate of polymer growth or shrinkage, potentially altering the overall behavior of the system (Horio and Murata, 2014). Resonance frequencies, once applied to non-linear damped systems, increase the amplitude of forced oscillations, challenging the initial conceptualization of the system. When the amplitude of these oscillations grows sufficiently large, the oscillator typically deviates from linear behavior, increases entropy, and starts exhibiting more intricate dynamics. During this phase, the forces that usually dampen oscillations are not the primary focus. As a result, the damped oscillation system, under these conditions, can develop increased complexity and potentially revert to chaotic behavior. On a macroscale, the external EMF that are modulated at harmonic and subharmonic frequencies could re-establish normal system dynamics, i.e., leading to a return to a normal state of bioelectric homeostasis (Figure 2) (Tagne Nkounga et al., 2023).

### 2.5 Electrophysiology of EMF

**FIGURE 3:**
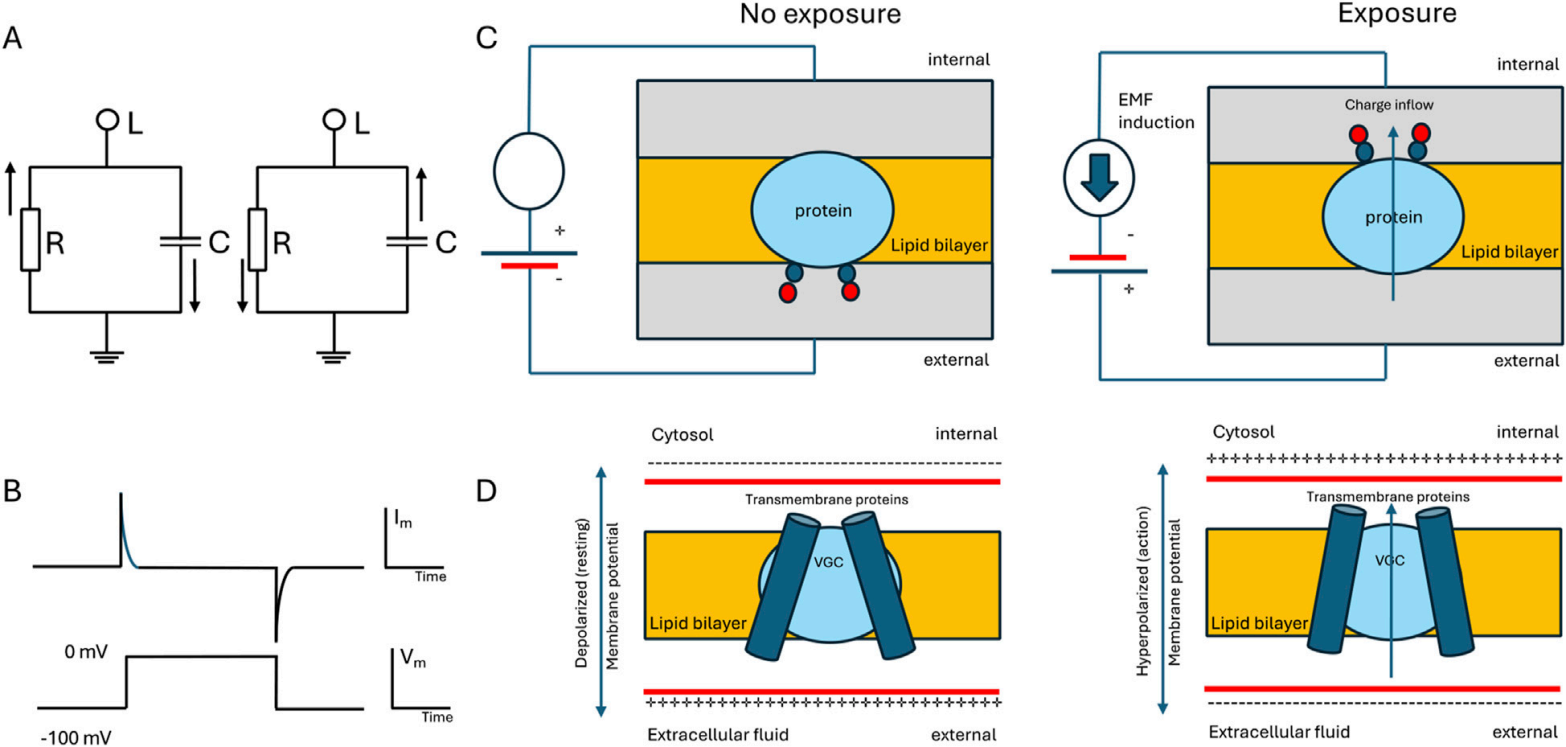
Simplified diagram illustrating the electrical characteristics of a plasma membrane and voltage-gated channels (VGC). **(A)** Circuit representation displaying the parallel arrangement of membrane capacitance and membrane resistance. RLC circuits exhibit resonant behavior by EMF exposure, alternating current (oscillation) at natural frequency. **(B)** Graphs depicting a command voltage step (bottom) and the corresponding current response when a basic plasma membrane is subjected to voltage clamping (top). **(C)** The example shows the membrane protein with two charges (positive charges are blue and negative countercharges are red). When the membrane voltage is reversed by EMF induction, these charges move from outside to inside of the membrane, crossing the entire electric field. **(D)** VGC model with transmembrane movement. Changing the membrane potential (from the left to right panels) causes VGC to open allowing the charges to move across the field of the lipid bilayer.

One of the direct targets of EMF effects are voltage-gated ion channels present in excitable and non-excitable cells (Pall, 2013). Cells exhibit a notable membrane potential, typically in the range of +50 to −90 mV range, characterized by a negative electrical potential inside the cell relative to the surrounding extracellular medium. This membrane potential plays a critical role in the cell cycle dynamics and is controlled by protein ion channels especially within the plasma membrane, especially through the action of voltage-gated channels that allow ions to flow in and out in a regulated manner due to their selectivity and active transport properties (Cervera, Alcaraz, and Mafe, 2016). Especially excitable cells encompass a variable dynamic behavior representing different forms of cellular synchronization (Nicola et al., 2018). Excitable cells are also known as “electrogenic cells” because they can generate and propagate an electrical potential across their membrane and produce endogenous EMF that express coupled effects locally and at a distance. The electrical potential is created by maintaining different concentrations of ions inside and outside the cell (Figure 3).

EMF exposure leads to changes in heart rate variability by just a few milliseconds at specific frequencies (Capareli et al., 2023). It is hypothesized that a direct interference of EMF in ion channels and the autonomic system regulation of myocytes is causal, and the mechanism could be well-explained at the plasma membrane level regulation of the cardiac rhythm. Sodium inactivating currents, known as I_Na_, are responsible for the swift depolarization phase in the initiation of the action potential (inward current activated on hyperpolarization), are known as natural pacemakers (DiFrancesco, 1986). These currents generate short-lived, inactivating currents rapidly within 1-2 milliseconds. This rapid inactivation of currents, combined with the slower onset of voltage-gated potassium currents, facilitates the membrane repolarization, thereby ending the electrical signal. The sinoatrial cells (e.g., cardiac pacemaker cells) have a very peculiar current known as “funny” channels that is a special inward sodium current triggered by hyperpolarization to diastolic voltage levels and cyclic adenosine monophosphate (cAMP) concentration. The phosphorylation-dependent process is relevant for funny channels activation and EMF exposure may reduce intracellular cAMP in humans (DiFrancesco, 1986; Laszlo et al., 2017). Its characteristics are apt for creating repetitive activity and adjusting the spontaneous rate of activity. The intensity of this “funny” current’s activation at the conclusion of an action potential affects the rate at which action potentials are fired (DiFrancesco, 1986). Action potentials, once the membrane threshold for activation is reached, always maintain the same amplitude, regardless of the strength of the stimulus. Such a threshold effect is typical for excitable dynamical systems, and is also referred to as the all-or-nothing principle in biology (Adrian, 1914).

While the role of excitable cells in generating and propagating electrical signals is essential for functions such as cardiac rhythm regulation, non-excitable cells utilize bioelectricity in a more subtle but equally crucial manner. These cells do not generate action potentials but instead maintain and communicate their bioelectric state through other mechanisms. This different use of bioelectric properties underlines the diversity of cellular functions and highlights the unique ways in which cells harness electricity to sustain life processes. Just as excitable cells rely on rapid changes in ion concentrations to transmit electrical impulses, non-excitable cells leverage their electrical state to orchestrate a wide array of physiological activities, establishing a crucial link between genetic patterns and complex body patterning (Moreddu, 2024). Non-excitable cells use ion channels to establish their electrical state and use electric synapses, by forming intercellular pores between adjoining cells known as gap junctions, to transmit their electrical state to adjacent cells. Gap junctions are specialized areas in the plasma membrane that contain numerous intercellular channels, facilitating the direct movement of ions and small molecules (with a molecular weight below approximately 1.2 kDa) between cells. These channels are composed of a family of transmembrane proteins known as connexin, and there are 20 distinct types/isoforms of connexins. Gap junctions are particularly important for a variety of key functions, including cell growth and differentiation control, and maintaining the homeostatic balance of tissues (Totland et al., 2020).

Cancer cells are non-excitable cells expressing lower resting membrane potential than normal cells contributing to their pathological behavior. Although these characteristics bear some resemblance to “excitable” tissues, the dynamics of the membrane potential in cancer cells is not well understood. Recent high-throughput, cellular-level membrane potential imaging has shown that the membrane potential in certain breast cancer lines (MDA-MB-231) fluctuates more dynamically compared to non-cancerous cells driven by voltage gated sodium channels (VGSC) (Quicke et al., 2022). MDA-MB-231 cells reveal electrical patterns characterized by asynchronous bipolar spikes, with average spike current magnitudes ranging from 40 to 140 pA. The temporal distribution of these spikes typically follows a Gaussian curve, peaking at around 30 ms (Ribeiro et al., 2020). However, like excitable cells, cancer cells have VGSC as observed in several cancer cell types (Roger et al., 2015). Their activation, triggered by membrane depolarization, occurs by generating transient sodium currents. Inward current through VGSC would sufficiently depolarize the membrane to activate other voltage gated ion channels such as calcium channels. Although voltage gated calcium channels do not require a sodium gradient, they can be activated by membrane depolarization, that occurs near VGSC (Levin, 2021). This altered behavior makes cancer cells more susceptible to EMF exposure. Extremely low frequency-EMF has been shown to hinder the growth of various malignant cell lines, while not affecting non-malignant cells (Buckner et al., 2015; Zimmerman et al., 2012; Jimenez et al., 2018).

In the cell, an intricate interplay among various calcium (Ca2+) transporters allow the cytosolic Ca2+ concentration to oscillate, similar to a radio signal (Smedler and Uhlén, 2014). This process involves encoding, where inputs are processed and translated into changes in cytoplasmic Ca2+ concentration, and decoding, which selectively influences the activity of specific Ca2+-sensitive targets (Salazar, Zaccaria Politi, and Höfer, 2008). This method of signal transmission is both durable and mechanistically fascinating, allowing for efficient encoding of specific information within the signal that can traverse the cell without causing damage. EMF stimuli can initiate a distinct Ca2+ signaling pathway. Furthermore, EMF stimuli that involve frequency and amplitude encoding are more effective than constant signals, showing a crucial dependence on the timing of inputs (Boulware and Marchant, 2008). The pattern of Ca2+ oscillation inputs can induce cellular phenotype selection processes and the Ca2+ oscillations plays an important role in governing phenotype selection and tissue architecture (Debir et al., 2021). The EMF exposure was found to enhance calcium inward flux through voltage-dependent Ca2+ channels and the inhibition of this calcium inward flux using Ca2+ channel blockers also halted the EMF-induced suppression of cell proliferation (Buckner et al., 2015; Jimenez et al., 2018). The increase in cytosolic Ca2+ concentration impacts on the cAMP and extracellular signal-regulated kinase 1/2 (ERK) signaling pathways that have a significant effect on the cell proliferation induced by EMF (Buckner et al., 2015). cAMP regulates cell signaling. In glioblastoma xenograft models, for instance, cAMP activators also induce tumor growth inhibition and differentiation, and mitochondrial biogenesis and metabolic switch to oxidative phosphorylation (anti-Warburg effect) driving the differentiation of tumor cells (Xing et al., 2017).

In cancer cells, the relationship between intracellular pH, cAMP levels, and membrane depolarization has complex dynamics that can significantly impact cancer cell biology (White, Grillo-Hill, and Barber, 2017). The altered intracellular pH (higher than in normal cells – more basic) and extracellular pH (lower than in normal cells – more acidic) in cancer tissue, the result of the Warburg effect, can affect cAMP levels (Liberti and Locasale, 2016). Enzymes involved in cAMP synthesis and degradation, like adenylyl cyclases and phosphodiesterases, can be sensitive to pH changes, thus potentially altering cAMP levels in response to pH fluctuations. Therefore, cAMP levels again can alter signaling activation and influence ion channels (e.g., voltage-gated sodium, calcium, and potassium channels) and transporter activity, which in turn can affect membrane depolarization (Liberti and Locasale, 2016). Thus, membrane depolarization can also impact intracellular pH regulation by altering the activity of ion transporters, including proton pumps and exchangers, affecting the intracellular (and extracellular) pH.

## 3 Clinical modalities for EMF exposure

Several applications, such as pulsed or alternating electric, magnetic, and electromagnetic fields, have been effectively tested in humans for both healthy and pathological conditions. Pulsed or alternating electric fields have been authorized as a standalone cancer treatment or as an adjunct to standard care. Two methods, namely Tumor Treating Fields (TTFields) and Electroporation, are commercially available in many parts of the world. These techniques employ high energy and offer localized or regional treatment options, as further explained below.

*TTFields:* marketed at Optune (NovoCure, Switzerland), is one of the physical approaches for cancer treatment approved and tested in several different cancer types [for recent review see reference (Tanzhu et al., 2022)]. TTFields employs alternating electric fields of a medium frequency (100–300 kHz) and low intensity (100–300 V/m). Research supporting this technology includes *in vitro*/*in vivo* studies and clinical evaluations that have demonstrated its efficacy in suppressing the growth of a range of tumors such as glioblastoma multiforme, lung cancer, malignant pleural mesothelioma, liver cancer, ovarian cancer, and pancreatic cancer, while also extending patient survival in randomized phase III trials (Stupp et al., 2017; Leal et al., 2023; Kutuk et al., 2023). Enhanced therapeutic outcomes are observed when TTFields technology is combined with combination treatments such as radiotherapy, chemotherapy, and other modalities (Leal et al., 2023; Stupp et al., 2017; Ceresoli et al., 2019). As a locoregional and non-invasive method, TTFields uses skin electrodes to emit the signal with TTFields, and is associated with minimal side effects, limited to mild to moderate skin reactions under the treatment electrodes, such as rashes, erythema, dermatitis, itching, and skin erosions, with rare instances of even severe skin conditions. However, the application of TTFields requires the patient to carry a cumbersome apparatus for 18 h a day (Tanzhu et al., 2022).

*Electroporation:* is a locoregional technique that uses needle(s) to emit the signal and utilizes pulsed electric fields (or EMF) to temporarily increase the permeability of the cell membrane. Electroporation occurs when cells are subjected to an external electric field, inducing a voltage field across the cell membrane that is directly related to the intensity of the applied field and persists for the duration of the field’s presence. Different from TTFields, this method involves applying high-voltage (100–130 kV/m) pulses of short duration (100 µs) at a frequency of 1 Hz (Gong et al., 2022). The Aliya System (Galvanize Therapeutics, Redwood City, CA) is one example of this technology. There are two main types of electroporation: reversible and irreversible. In reversible electroporation, the cells can repair the induced artificial membrane pores, allowing them to regain their normal functions and remain viable. This facilitates, for example, the enhanced delivery of drugs, ions, genetic materials, and proteins into the cells without significantly compromising their survival (Frandsen, Vissing, and Gehl, 2020). On the other hand, irreversible electroporation occurs when the induced voltage exceeds a certain threshold, resulting in permanent membrane damage and ultimately leading to cell death (Davalos, Mir, and Rubinsky, 2005). The effects of electroporation extend beyond the creation of membrane pores; it also involves the activation of various cellular signaling pathways, the release of calcium from the endoplasmic reticulum, disruption of mitochondrial membrane potential, destruction of the cytoskeleton, and cellular processes such as swelling, blebbing, and apoptosis (Gong et al., 2022).

**FIGURE 4:**
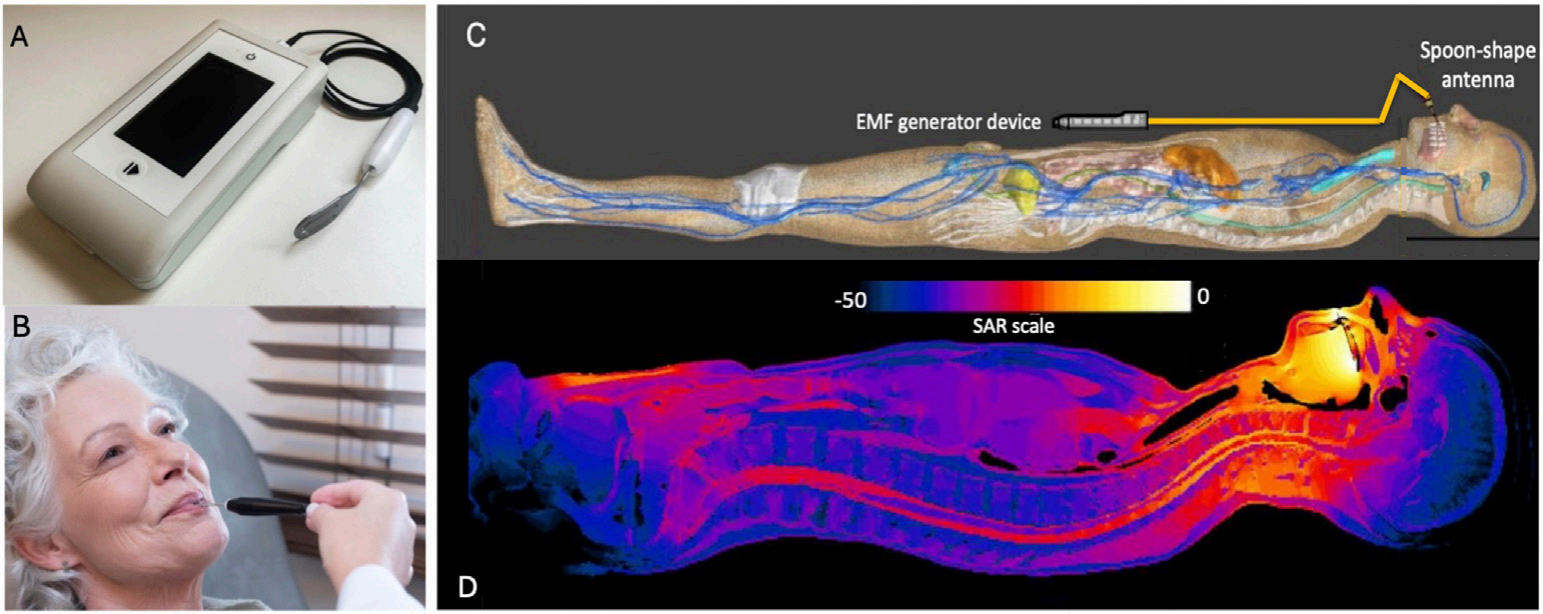
**(A)** Medical device from Autem Therapeutics with spoon-shaped antenna with cable. **(B)** The spoon shaped antenna is positioned comfortably within the oral cavity, situated between the tongue and the hard palate. To maintain its proper placement through the exposure procedure, a plastic holder is employed. **(C)** Distribution of SAR in the Duke Model. The model is presented in supine position near the medical device. The computer virtual model represents different organs with their corresponding dielectric properties when device is positioned in the abdomen. The SAR distributions is sensitive to the position of the medical device. **(D)** The EMF signal propagates systemically throughout the body originated from the antenna placed in the patient’s mouth. The simulation shows SAR results at different body locations using a color scale being the higher SAR in yellow and lower SAR in blue.

**FIGURE 5:**
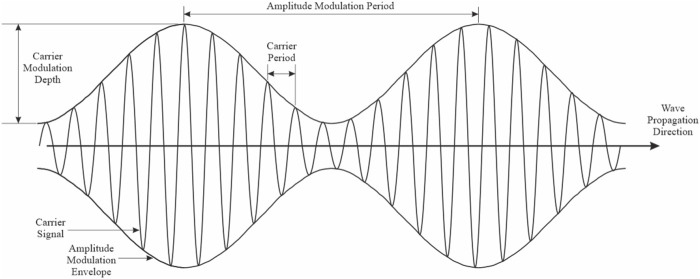
Schematic description of time-variant EMF. The carrier frequency is sinusoidally amplitude modulated (envelope frequency).

Unlike TTFields and Electroporation, which are locoregional treatments, time-variant EMF provides a systemic treatment approach. This modality uses lower energy and is considered safe as a standalone cancer treatment or in combination with standard care. Time-variant EMF is still experimental and is based on the principles of oscillatory coupling and resonance, that are discussed in the present manuscript. Time-variant EMF represents a new technique that was tested in humans in health and disease [for reviews and references see (Zimmerman et al., 2013; Blackman, 2012; Wust et al., 2022; Jimenez et al., 2018)]. Time-variant EMF uses radiofrequency (RF) emission with a fixed carrier wave but at a lower power rating, i.e. 100–150 mW, that generates an electric field of only 3–5 V/m (at the site of action) as compared to 100–300 V/m in the case of TTFields. Furthermore, due to the low energy exposure, time-variant EMF is emitted not transdermally but intrabuccally by an oral antenna applicator (Figure 4). The body becomes an antenna, thereby the emitting energy is transmitted systemically with distribution throughout the entire human body (Robert, 2022; Jimenez et al., 2018; Capareli et al., 2023). Thus, time-variant EMF technique is a systemic therapeutic approach, not locoregional as the other EF applications. Time-variant EMF produces a sinusoidal alternating current like TTFields. It is similar to a wavy pattern of electric flow that changes over time. Similarly, for a sinusoidal alternating voltage, at a certain time t, V is the maximum voltage (amplitude) it reaches. Time-variant EMF is safe, has no limiting toxicity and has a quite different treatment profile as described by the high energy electric field techniques. The time-variant component of the signal is due to sinusoidal shape of the carrier wave and the AM wave, both at different frequencies (Figure 5) (Tuszynski and Costa, 2022). Thus, time-variant EMF sends two simultaneous signals: the carrier wave at high frequency (27–30 MHz) and the AM signal at a very low and extremely low frequencies (10 Hz–20 kHz). Both waves are oscillatory signals that carry “therapeutic” specificity. For this reason, this technology is known as low-energy radiofrequency amplitude modulated electromagnetic fields (RF AM EMF). The specific oscillatory signal frequency at very low frequency/extremely low frequency range allows EMF to couple with the other oscillatory bioelectric patterns generated in the human body, tissues, living cells and cancer. If RF AM signals are applied at a much higher frequency range (e.g., those used by mobile phones), no coupling effects are observed due to large frequency ratio between the two oscillators (external and internal) (Heltberg et al., 2021). Based on the bioelectrical characteristics of the human body, time-variant EMF carrier wave has a wavelength of approximately 1.3 m in the human body (data from IT’Is Foundation, Zurich Swiss) (Gosselin et al., 2014). Wavelength is the distance between two consecutive peaks of carrier wave. Shorter wavelengths have higher energy, while longer wavelengths have lower energy. The energy absorption is wavelength specific depending on the dielectric properties of the object. Thus, a 27–30 MHz carrier wavelength matches the target size area of the human body, and hence facilitates resonance to occur (Funk, Monsees, and Ozkucur, 2009).

## 4 Direct EMF effects

Numerous medical applications of EMF have been proven to cause direct effects in the human tissues (Tuszynski et al., 2020; Priel, Tuszynski, and Woolf, 2005; Gordon, 2007). For example, the effects of EMF in magnetic resonance imaging (MRI) are a well-known diagnostic application. However, there are also several therapeutic uses for EMF. These include stimulation of the vagus nerve (Cimpianu et al., 2017; Wheless, Gienapp, and Ryvlin, 2018), treatment of bone fractures (Bassett, Pawluk, and Pilla, 1974; Patruno et al., 2010; Ross et al., 2017), wound healing (Gualdi et al.), pain management (Gibson, Wand, and O'Connell, 2017), and conditions affecting the nervous system like refractory epilepsy (Wheless, Gienapp, and Ryvlin, 2018), depression (Cimpianu et al., 2017; Larsen et al., 2020), nerve regeneration (Ross et al., 2017), and dementia (Cimpianu et al., 2017). Direct effects by exposure to low-energy EMF are measurable in the human body as measured through alteration in the heart rate variability (Wallace et al., 2020; Misek et al., 2020) and changes in brain electrical activity during transition from wakefulness to sleep (Loughran et al., 2012; Huber et al., 2003). These examples highlight how EMF can produce a wide range of effects in the human body and its biological functions, which are controlled by electrical potentials and currents, by altering the electrochemical balance in cells.

### 4.1 Cellular effects

It is now well-understood that all cells, particularly cancer cells, exhibit distinct electrical characteristics which are critical in determining their physiological behavior (Di Gregorio et al., 2022; Nasir and Mahmoud, 2020; Ahmad et al., 2018). EMF propagation in biologically active environments yields important computational and theoretical insights (Razek, 2022). The complex interactions between EMF energy and cellular entities involve a range of physicochemical processes and it is influenced by the specific geometry and dielectric characteristics of the medium. The variability in biological tissues and cells, due to their diverse dielectric properties and ionic conductivities, adds an additional layer of complexity to these interactions, which are crucial for a full understanding of EMF effects on biological systems. This has been substantiated by an extensive body of research focused on the electrostatic attributes of various cell types highlighting significant differences between cancer cells and their normal counterparts [for reviews see references (Di Gregorio et al., 2022; McCaig et al., 2005; Robinson and Messerli, 2003; Pullar, 2016)]. Among the noted differences, the variation in pH levels inside and outside the cells stands out. Specifically, cancer cells tend to create an acidic environment outside the cell (lower extracellular pH) and a more alkaline environment inside (higher intracellular pH), a phenomenon often referred to as “proton gradient reversal,” observed in cancer cell (Zeng and Hu, 2023; Warburg, 1956; Chiche, Brahimi-Horn, and Pouyssegur, 2010). Additionally, when comparing the plasma membrane resting potentials, cancer cells typically exhibit less negative values than those of normal cells (Yang and Brackenbury, 2013; Marino et al., 1994; Quicke et al., 2021). Both aspects are attributed to the Warburg effect, a hallmark of cancer (Warburg, 1956; Hanahan and Weinberg, 2011). Moreover, Ca2+-dependent signaling mechanisms are often remodeled or deregulated in cancer cells, indicating that the alterations in Ca2+ signaling may be due to epigenetic changes in gene expression and/or post-translational modifications of existing signaling components [for review see reference (Roderick and Cook, 2008)]. Those differences also make cancer cells more susceptible to EMF interference. The Warburg effect and Ca2+-dependent signaling pathway may explain (besides other causes) why cancer cells and non-normal cells can undergo apoptosis induced by EMF (Buckner et al., 2015; Zimmerman et al., 2012; Patergnani et al., 2020). The systemic administration of modulated EMF, notably within the range of 30 kHz–300 GHz, is pivotal in numerous applications in different areas, including telecommunications, medical diagnostics, and therapy (Mattsson and Simkó, 2019). Given that biological materials can absorb this energy, there is increasing focus on how humans, exposed to these fields, are affected (Taki and Watanabe, 2001; Saliev et al., 2019). EMF exposure has been shown to induce sustained objective radiological tumor responses, improve quality-of-life and may prolong survival in oncological patients without significant adverse effects in phase 1 and 2 prospective clinical trials (Buckner et al., 2015; Barbault et al., 2009; Costa et al., 2011; Capareli et al., 2023). The molecular and organellar targets for EMF in cancer cells have been elucidated by *in-vitro* and *in-vivo* investigations in a variety of different immortalized cell cultures and xenograft animal tumor models (Moreddu, 2024; Tuszynski and Costa, 2022; Zimmerman et al., 2012; Filipovic et al., 2014; Jimenez et al., 2018) demonstrating the following:

1. An alteration of the cellular membrane impedance contributing to altered cellular dynamics such as enhanced proliferative activity, diminished apoptotic processes, increased migratory directionality, and an augmented invasive potential;
2. An alteration of voltage-gated ion channels behavior leading to fluctuations in membrane depolarization and transduction pathways;
3. An alteration in metabolism known as the Warburg effect, i.e. leading to an abnormal aerobic glycolysis with consecutive lactate anion secretion that leads to a decreased extracellular pH value (acidity) and increase intracellular pH value (basicity); and
4. An alteration of the microtubule polymerization, mitotic spindle formation and mitochondrial trafficking.

### 4.2 Subcellular effects

**FIGURE 6:**
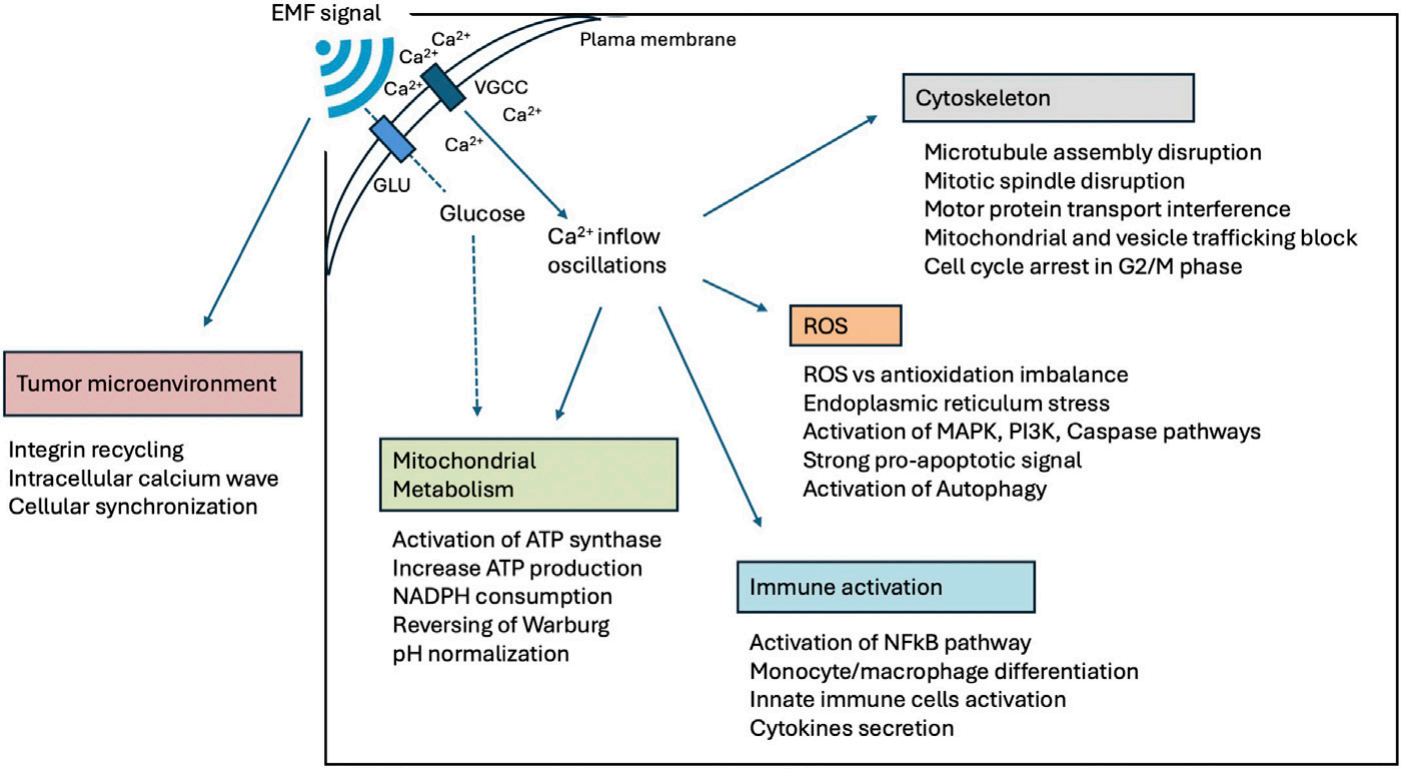
Epigenetic effects of extremely low-frequency EMF on cell response reported by *in-vitro* and *in vivo* experiments.

Pinpointing, isolating, and quantifying the specific effects of EMF on the tissues of a complex living organism remains a formidable challenge that demands sophisticated technology. Conversely, numerous studies have consistently shown that EMF exposure can commonly alter various intracellular processes in both normal and cancerous cells. Most effects have been produced and described through translational studies conducted *in-vitro* and *in-vivo* using EMF showed a significant inhibitory effect on tumor cells and did not affect the growth of normal cells (Zimmerman et al., 2012; Cui et al., 2014; Delle Monache et al., 2008; Feng et al., 2016; Mousavi Maleki et al., 2022; Schuermann and Mevissen, 2021; Sun et al., 2023). Despite significant variability among experimental techniques (e.g., EMF exposure device, time of exposure, and field intensity), several epigenetic results consistently demonstrate the effects of EMF in cancer cells (Figure 6). Of note, some experiments used identical setup tested in prospective clinical trials (Zimmerman et al., 2012; Jimenez et al., 2016; Jimenez et al., 2019; Sharma et al., 2019). A number of these *in-vitro* and *in-vivo* experiments using extremely low-frequency EMF exposure and low energy fields (below 2 mT) are summarized in a supplement tables (see Supplementary Material with 78 references). The fundamental extremely low-frequency EMF effects are discussed in more detail below.

*Voltage gated ion channels:* EMFs exceeding 1 MHz in carrier frequency deliver energy to tissues primarily through ionic and dielectric losses. Initial theories postulated that stimulating cell membranes would require EMF intensities up to the very high levels of 200 kV/m. By contrast, practical studies have demonstrated that even EMF levels even below 10 V/m can disrupt cellular bioelectric functions (Tuszynski and Costa, 2022). Molecules with electric charges or dipoles, integral to the plasma membrane’s bilayer structure, are sensitive to electric disturbances and they are candidates for non-linear behavior since they cannot rotate within the membrane and dissipate energy (Nawarathna et al., 2005). This combination of protein conformational changes and ion translocation creates a non-linear response manifested by the generation of harmonics (Bezanilla, 2008). The EMF impact on these molecules is significantly magnified, with an amplification factor around 1,000 due to the application of Gauss’ law (Tsong, 1988; Nawarathna et al., 2005). Under EMF, membrane proteins can shift structurally, altering their ionic affinity. EMF can externally prompt the transport of potassium and calcium ions (Tsong, 1988). Alternating fields (e.g., sinusoidal EMF) affect cellular membrane permeability by imposing an EMF gradient that forces voltage-gated ion channels to adapt to an “open” state and thus cause increased ionic and molecular permeability (Aguilar et al., 2021). Furthermore, ion channels act as half-waves for the membrane’s electric field (voltage), transforming the EMF’s sinusoidal waveform perpendicular to the membrane into singular positive half-waves (Wust, Kortum, et al., 2020; Wust, Stein, and Ghadjar, 2021). In other words, EM sinusoidal waveform produces AC oscillation curve that describes a smooth, periodic shape of a sine wave. A rectifier converts AC, that periodically reverses direction, to direct current (DC), which flows in only one direction. A half-wave rectifier specifically allows only one-half of the AC sinusoidal waveform to pass through, blocking the other half. The result is that the output waveform has positive “half-waves,” with the negative part of the sine wave being clipped off or suppressed.

Computational modeling also points out that sensitivity to EMF stimuli varies widely, between an organ or cellular level with electric potential gradients ranging between 0.1 mV and 100 mV. EMF induction of electric fields further illustrates how external electric fields, when penetrating cellular membranes, can intensify locally, leading to significant impacts on membrane potentials even at relatively low field amplitudes applied externally (Aguilar et al., 2021). That is, even low energy fields can significantly alter the plasma membrane potential. On a microscale, the external electric fields traverse the cell membrane locally and penetrate the cytoplasm through membrane channels. The amplitude of the applied electric field across the membrane can vary between 2 kV/m and 75 kV/m with E_applied_ = 10 V/m. In accordance with Gauss’ law, localized cell focusing occurs, leading to a concentration of field lines in high conduction areas and separation in insulating spaces (Aguilar et al., 2021; Tsong, 1988). Therefore, ion channels and cleavage furrows in dividing cells may serve as specific regions where EMF intensities penetrate the cell, potentially influencing membrane potentials, even with relatively low amplitudes of EMF.

Cellular reactions to EMF vary significantly between two key setups: cells that are freely floating in an electrolyte solution and those directly adhering to a surface. Epithelial cells, for instance, can have part of the plasma membrane anchored to the basement membrane and part embedded with the extracellular matrix. In the case of a plasma membrane that lacks surrounding electrolyte, even low-frequency EMF can penetrate the cell membrane where the floating part remains unaffected by low-frequency EMF. Thus, plasma membrane of epithelial cells shows spatially varied EMF effects (Taghian, Narmoneva, and Kogan, 2015).

**FIGURE 7:**
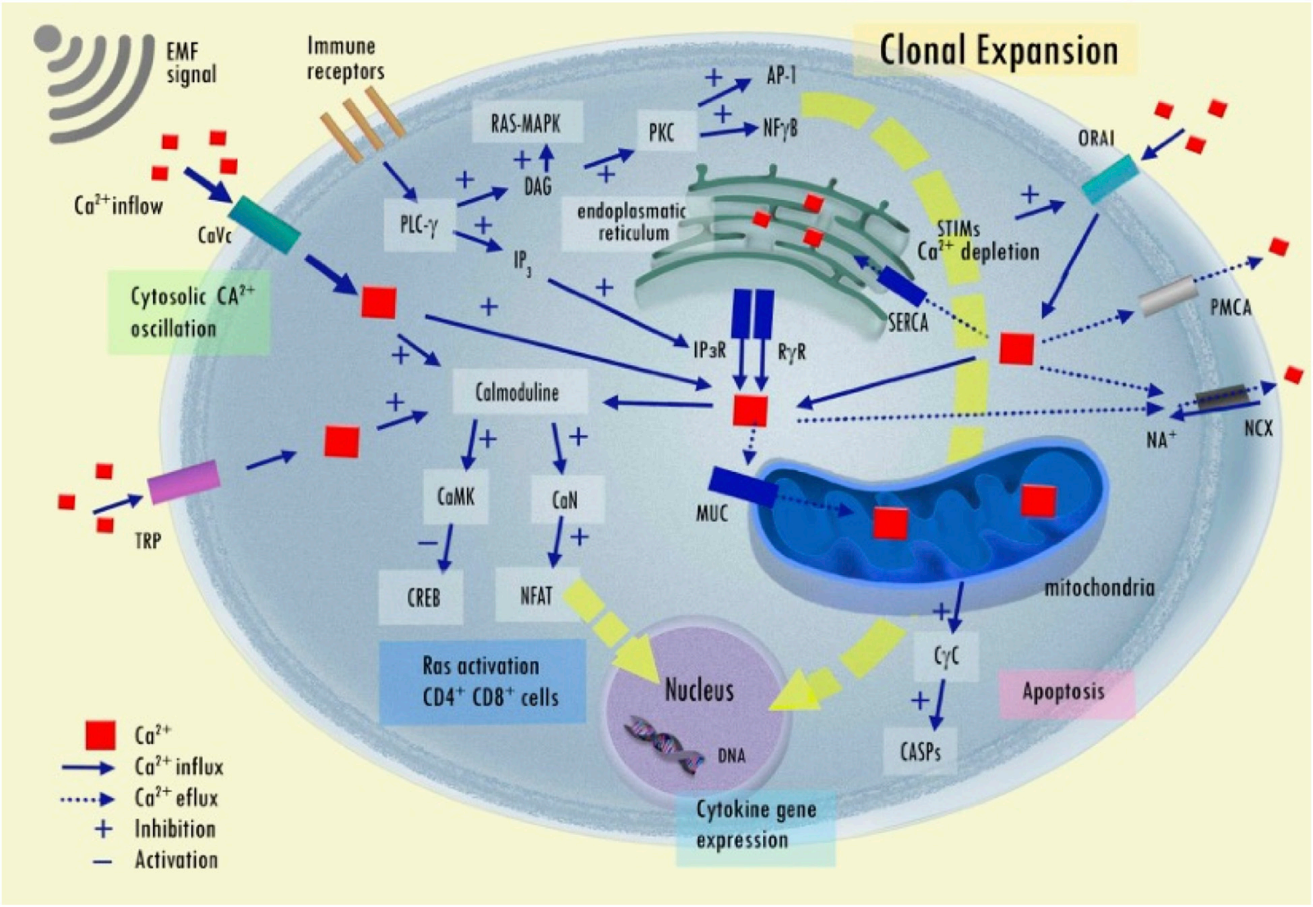
The cytosolic free Ca2+ concentration is maintained at approximately 100 nM regulated by intracellular stores (endoplasmic reticulum, Golgi or lysosomes). Calcium Influx (entry across the plasma membrane) can increase to >1 μM. EMF can trigger Ca2+ influx and produce an intracellular Ca2+ wave. This figure exemplifies long-term effects of Ca^2^⁺ signaling the encompass a range of critical cellular functions, including the proliferation of lymphocytes and the expression of genes associated with activation. It also influences effector functions, such as cytokine and chemokine production, and guides the differentiation of naive T cells into various effector or memory T cells. However, the Ca2+ signaling is often remodeled in cancer cells to sustain proliferation and avoid cell death.

Several studies suggested that voltage-gated calcium channels (VGCC) are one of the direct targets of extremely low frequency-EMF (for review see references (Cui et al., 2014; Pall, 2013)). The result is an increase in cytosolic levels of Ca2+ (Jimenez et al., 2019; Nezamtaheri M. S. et al., 2022; Buckner et al., 2015). EMF can trigger calcium influx, leading to the spread of calcium waves across cells nearby. These intercellular calcium waves (ICWs) are characterized by increased cytoplasmic calcium ion concentrations that propagate from a trigger cell, affecting numerous surrounding cells and enabling them to synchronize and coordinate cellular functions, as described in astrocytes and hepatocytes (Gaspers and Thomas, 2005; Cornell-Bell et al., 1990; Charles, 1998; Giaume and Venance, 1998). Oscillations of cytosolic calcium are efficiently transmitted to the mitochondria as mitochondrial calcium oscillations. Mitochondrial calcium spike is sufficient to activate and sustain mitochondrial metabolism (Hajnóczky et al., 1995; McCormack and Denton, 1993). Moreover, these functions include protein kinase C activation and vesicle secretion coordinated by Ca2+-calmoduline pathway (Figure 7) (Hogan et al., 2003; Roderick and Cook, 2008). ICWs are crucial in various biological processes like the enhancement of ciliary activity in airway cells, cellular proliferation, differentiation, and vascular tone regulation through signaling pathways involving different cell types. Additionally, ICWs facilitate critical interactions within the body’s different systems, including neuronal and vascular communication (Charles, 1998; Cornell-Bell et al., 1990; Dora, 2001; Gaspers and Thomas, 2005; Giaume and Venance, 1998; Leybaert and Sanderson, 2012; Nathanson et al., 1995; Scemes and Giaume, 2006). The ICW would have different effects on cancer cells that are under oxidative stress and proliferation (Görlach et al., 2015; De Nicolo et al., 2023).

**FIGURE 8:**
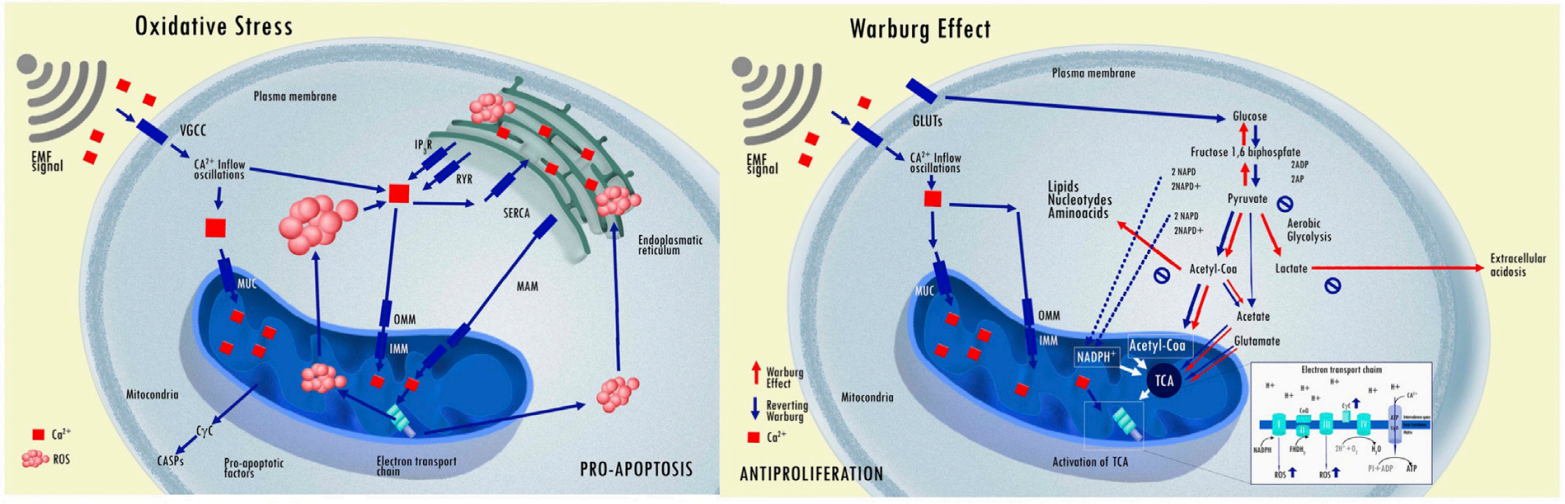
Intracellular Ca2+ ions serve as second messengers that control gene transcription, cell growth, movement, and death. In cancer cells, the balance of intracellular Ca2+ is disrupted. Electromagnetic fields (EMF) inducing influx of Ca2+ into the cytosol and mitochondria can trigger a potent pro-apoptotic signal in cancer cells under oxidative stress, and potentially halting cancer growth by reversing the Warburg Effect.

*Mitochondrial function:* Extremely low frequency-EMF signals result in an increase in the Ca2+ levels in the cytosol and mitochondria (Jimenez et al., 2019). Moreover, Ca2+/Calmodulin-dependent binding is the principal transduction pathway representing a dynamic, highly versatile, and crucial pathway involved in life-and-death decisions that determine a cell’s fate (Roderick and Cook, 2008). Ca2+/Calmodulin is activated by non-thermal EMF (Pilla et al., 2011; Roderick and Cook, 2008). Mitochondria are pivotal organelles in energy generation and cellular regulation, notably through the Electron Transport Chain (ETC) fostering ATP production alongside the generation of mitochondrial Reactive Oxygen Species (mROS), that are tightly regulated by efficient antioxidant systems. EMF can induce moderate increases in mROS. Cellular ROS include free radicals such as superoxide (O_2_-) and hydroxyl radical (-OH), and non-radical species such as hydrogen peroxide (H_2_O_2_). ROS are generated at the plasma membrane, through NADPH-dependent oxidases, and at the mitochondria by the incomplete one-electron reduction of oxygen at respiratory chain complexes I and III. mROS production is important in humans. It is estimated that, in the mitochondrial respiratory chain, about 2% of the O_2_ produces superoxide radicals (D'Autreaux and Toledano, 2007; Schuermann and Mevissen, 2021; Murphy, 2009). Cancer cells respond to elevated ROS levels by activating antioxidant pathways, enhancing ROS elimination (Nakamura and Takada, 2021; D'Autreaux and Toledano, 2007; Schuermann and Mevissen, 2021). This adaptation renders cancer cells more vulnerable to therapies that induce ROS production. Elevated ROS within cancer cells can impede cell growth and trigger apoptosis. Excessive ROS levels damage various cellular components, including proteins, nucleic acids, lipids, membranes, and organelles, prompting cell death pathways like apoptosis. Ca2+ pathways and mitochondria serve as key players in initiating apoptosis, acting as both generators and recipients of ROS (Nakamura and Takada, 2021; Dejos, Gkika, and Cantelmo, 2020; Sohn and Cooper, 2023) (Figure 8).

It is important to realize that the Warburg effect is vital for cancer cells. According to Warburg hypothesis, that suggests that aerobic glycolysis plays a significant role in tumor growth, altered mitochondrial function and increased glucose utilization for energy are characteristic features of many proliferating tumors (Warburg, 1956). The Warburg effects is associated with identified malfunctions in mitochondrial ETC complexes in human cancers, and it only happens in normal proliferating cells during mitosis (Sun et al., 2019). Once normal cell cycle starts, the cells begin to rely on glycolysis for ATP generation followed by ATP hydrolysis and lactic acid release, to maintain the elevated intracellular pH as needed by cell division since together the three processes are pH neutral (Sun et al., 2019). That would be one of the explanations why EMF only affect cancer cells and spare normal cells from apoptosis. Epidemiological research has indicated that a defective Complex I may serve as a biomarker for aggressive forms of thyroid, breast, colon, and other cancers. Additionally, mutations in Complex III and Complex IV (cytochrome c oxidase) have been frequently observed across multiple cancer types (Watson and McStay, 2020). In most of these instances, point mutations and deletions in mitochondrial DNA (mtDNA) have been linked to the impaired assembly and function of these ETC complexes (Watson and McStay, 2020).

Cytochrome c oxidase (CcO) is the final enzyme in the electron transport chain of mitochondrial oxidative phosphorylation, essential for energy production in eukaryotic cells. This enzyme facilitates the transfer of electrons from electron carriers to molecular oxygen, producing water. It also helps move protons from the mitochondrial matrix to the inter-membrane space, creating an electrochemical gradient. This gradient drives the synthesis of ATP, powering critical cellular functions (Watson and McStay, 2020). Dysfunction in this enzyme, particularly CcO, is linked to the Warburg effect in cancer cells, which contributes to cancer progression by altering energy metabolism (Sun et al., 2019). The activity of CcO can be affected by EMF, with the reaction rate constants displaying a dependence on frequency that ranges from 10 to 2,500 Hz. This behavior is consistent with known responses of other membrane enzymes to electric and magnetic fields, where the optimal frequencies correlate with the natural turnover rates of the enzymatic reactions (Blank and Soo, 1998).

EMF induces mitochondrial permeability transition (MPT) by opening mitochondrial permeability transition pores (Feng et al., 2016; Endlicher et al., 2023). MPT is known by changes in the permeability of the inner mitochondrial membrane, that becomes highly permeable to solutes and low molecular weight substances. It is a physiological event that can occur under certain pathological conditions such as calcium overload, oxidative stress, or damage to the mitochondrial matrix, affecting ATP production (Hunter, Haworth, and Southard, 1976). Mitochondria operate as a buffer to regulate cytosolic Ca2+. The uptake of Ca2+ into mitochondria plays a crucial role in molding cellular Ca2+ signals and serves as a pivotal factor for ATP production (Delierneux et al., 2020). Moreover, mROS that is generated during ATP synthesis at the ETC regulate cellular signaling pathways that influence various cellular processes (Delierneux et al., 2020). Cancer cells manipulate the homeostasis of mitochondrial Ca2+ by modifying the expression and activity of mitochondrial Ca2+ channels and transporters responsible for the uptake and removal of mitochondrial Ca2+. Mitochondrial Ca2+ modulation influences tricarboxylic acid (TCA) enzyme activity, potentially driving mROS production and altering NAD+/NADH ratios (Delierneux et al., 2020; Nezamtaheri M. S. et al., 2022; Keating and El-Osta, 2023). In summary, we hypothesize that EMF exposure leads to reversal of the Warburg effect arresting cancer proliferation. EMF also leads to increased levels of calcium in the cytosol and in the mitochondria that can induce MPT and, together with high levels of mitochondrial ROS that disrupt ROS equilibrium, create a harmful feedback loop that severely impairs mitochondrial function. This can result in decreased ATP production, increased oxidative damage, and may eventually lead to cell death via necrosis or apoptosis, contributing to the pathology of various diseases. Moreover, mitochondria exhibit spontaneous fluctuations in the mitochondrial inner membrane potential, that can be significantly diminished by treatments altering mitochondrial activity (Buckman and Reynolds, 2001; Vergun, Votyakova, and Reynolds, 2003). The inner membrane potential serves as a crucial indicator of mitochondrial function, crucially involved in proton pumping across the inner mitochondrial membrane alongside electron transport. These processes drive various cellular functions including ATP synthesis, calcium accumulation, and the maintenance of ion gradients necessary for substrate influx and metabolic product efflux within the cell (Vergun, Votyakova, and Reynolds, 2003; Buckman and Reynolds, 2001; Lai and Levitt, 2023; Kivrak et al., 2017) (Figure 8).

Finally, the precise subcellular positioning of mitochondria is closely linked to their bioenergetic and cell signaling functions. Mitochondria dynamically adjust their location within cells by traveling along microtubules, being propelled by motor proteins, and they may also form a network that facilitates energy and signal exchange between cells through membrane nanotubes. Disruption of microtubules by EMF can interfere with mitochondrial trafficking, potentially affecting multiple mitochondrial functions. While cytoplasmic Ca2+ has been identified as crucial for mitochondrial motility in neurons, the relationship between Ca2+ levels and mitochondrial motility remain unclear in cancer cells. However, Ca2+ levels are known to play a crucial role in mitochondrial movement and that there exists a critical Ca2+ threshold necessary for pausing mitochondrial mobility (Chang, Niescier, and Min, 2011; Tuszynski and Costa, 2022).

*Integrins:* Integrins are among the most abundant cell surface receptors. Integrins are transmembrane cell surface proteins that play a crucial role in sensing stress and facilitating bidirectional signaling during cell interactions with other cells or the extracellular matrix (ECM) (De Franceschi et al., 2015). The ECM is a complex structure that is carefully shaped and controlled by the molecules that make it up and the power of the cells that live in it. ECM differs based on the type of cell and tissue, helping cells work properly and giving tissues their characteristics. The ECM has a network of molecules crucial for growth, healing, and recovery from injuries. It serves as a hub for signaling molecules, including those that cause inflammation, sending messages to cells that are growing, changing, or moving.

Integrins are one of the most important groups of molecules that can effectively regulate cell migration linking the extracellular matrix to the cell’s interior (Caswell, Vadrevu, and Norman, 2009; Takada et al., 2007). Integrins, influencing signal transduction pathways and the organization of the cell cytoskeleton (De Franceschi et al., 2015; Cefalas et al., 2021). Sinusoidal EMF (e.g., oscillatory force) generates forces of comparable magnitude on integrins. These forces mechanically couple to the actin cortex, resulting in corresponding internal force and torque. Integrins are depicted as cylindrical structures extending through the cell membrane and connected to a network of springs (cytoskeleton), without external support from the extracellular matrix, enabling them to oscillate freely in a surrounding medium (Hart, 2006). When an oscillating force is applied, it interacts on the integrins, and their coupling to the cytoskeleton follows a similar pattern. With thousands of integrins on each cell’s surface oscillating in phase, and mechanical coupling between cytoskeletal elements within and between cells, the entire system behaves as a network of damped oscillators, driven in phase. This suggests that the fundamental transduction mechanism for certain electric field effects may ultimately be mechanical in nature (Hart, 2006). EMF interaction with integrins modulates migration speed, persistence, and direction of cells, important in many biologic processes, such as wound healing and immune surveillance (Zhu et al., 2019; Isakovic et al., 2018).

Recently, it has been demonstrated that the molecular pathway facilitating integrin recycling within cells also aids in mitochondrial localization (O'Sullivan and Lindsay, 2020). The accumulation of integrin prompts the formation of an adhesion complex at the cell membrane, consequently directing mitochondria towards the cell peripheries. These discoveries indicate that pathways promoting mitochondrial dispersion might confer resistance to therapies targeting cell death through increased reactive oxygen species production. Another example are vesicles carrying various proteins. They all have similar characteristics: they move in both directions and switch directions frequently, but overall, they mostly move forward at speed around 1 μm/s (Nakata, Terada, and Hirokawa, 1998). This newfound association between cell movements, mitochondrial and protein dynamics could elucidate the invasive nature of certain cancers and their resistance to ROS. These findings could pave the way for innovative strategies to enhance the effectiveness of ROS-based cancer treatments, such as EMF exposure (O'Sullivan and Lindsay, 2020).

*Microtubules:* microtubules are hollow cylindrical structures whose building blocks are αβ tubulin dimers, which are electrically charged and act as biological ionic conduction “wires”. Recent computational and theoretical analyses indicate that electrostatic forces generated by clinically significant EF amplitudes (100–300 V/m) are insufficient to notably influence the rotational dynamics of tubulin or septin (Tuszynski et al., 2016; Li and Wang, 2014). However, microtubules exposed to AC electromagnetic frequencies may become up to 1,000 times more conductive than the cytoplasm, that can lead to the disruption of cellular processes. Resonance peaks for microtubules have been reported to fall in the range of frequencies of 12, 20, 22, and 30 MHz (Sahu et al., 2014). Of note is the so-called ballistic conductivity of microtubules in the 12–30 MHz range reported in reference (Tuszynski et al., 2020). Moreover, electric fields with amplitude values of 100 V/m are sufficiently strong to exert dielectrophoretic effects on tubulin and microtubules. The longer the microtubules, the more pronounced the dielectrophoretic effect is predicted to occur. Moreover, microtubule’s C-termini oscillate dynamically in the MHz range frequencies (Priel, Tuszynski, and Woolf, 2005). Importantly, the length of microtubules determines the value of the resonant frequency, which can be cell- and patient specific. Other factors, such as cytoplasmic viscosity, pH, ionic concentrations (especially calcium, which depolymerizes microtubules) and the post-translational modifications of tubulin, may play a major role in determining the response of microtubules to AC EF (and EMF) in various frequency ranges. These frequencies cause electromagnetic interference that appears to disrupt motor protein traffic in a living cell, with important downstream effects on cellular dynamics. Microtubules may also be influenced by the low-frequency effects of the envelope wave (e.g., AM coupling). The structural wall of the microtubule’s cylinder is interspersed with nanopores formed by the lateral arrangement of its monomers. Applying the patch clamp technique to two-dimensional microtubule sheets, researchers were able to characterize their electrical properties and generated cation-selective oscillatory electrical currents whose magnitude depended on both the potential and the ionic strength and composition of the solution (Cantero et al., 2018). The oscillations showed a fundamental frequency at 29 Hz. Under physiological K+ concentration (140 mM), oscillations caused, on average, a 640% change in conductance (a measure of how easily electricity can flow through a material) that was also affected by the prevalent anion concentration. Current injection induced voltage oscillations, thus showing excitability akin to action potentials. These findings suggest a functional role of the nanopores in the microtubule’s wall in the genesis of electrical oscillations. Periodic changes in the amplitude of oscillations showed fractal envelopes with a fundamental frequency of 39 Hz (Cantero et al., 2018). Those amplitude of oscillations can be altered by EMF.

Cell Cycle Regulation: cell cycle arrest is a specific process related to the regulation and progression of the cell cycle, which involves multiple mechanisms and pathways that can be altered by EMF. Depending on the cell type and the intensity and nature of the stress, cells may respond by attempting repair or initiating cell death if DNA damage remains unresolved. The cell cycle, a series of coordinated events leading to cell division, is regulated by checkpoints at the G1/S boundary, during S-phase, and in the G2/M phases. During early G1, Cyclin D associates with Cdk4 and Cdk6, targeting the pRb family of proteins. Phosphorylation of pRb proteins allows the transcription of genes necessary for S-phase entry. Cyclin D/Cdk complexes are crucial for linking extracellular signals to the cell cycle; upon mitogenic stimulation, these complexes are activated, prompting cells to progress from G0 into G1. Once the restriction point (R-point) in late G1 is passed, the cell commits to entering S-phase, and cyclin D/Cdk activity is no longer needed for cell cycle progression. Cyclin E activates Cdk2 during the G1-to-S-phase transition. During S-phase, Cyclin A binds to Cdk2, and during the G2-to-M-phase transition, it binds to Cdc2. The Cyclin B/Cdc2 complex also functions during the G2-to-M-phase transition, ensuring proper progression through the cell cycle. Flow cytometry experiments showed that ELF-EMF exposure lead to a significant increase in the accumulation of cells at the G2/M phase (Mehdizadeh et al., 2023; Liu et al., 2016; Nezamtaheri MaryamSadat et al., 2022). Those studies identify several gene expression that were impacted by EMF exposure that may alter cell cycle progression, however calcium influx disrupts intracellular calcium homeostasis and induces stress-responsive protein kinases, such as p38 MAPK and JNK, which play critical roles in cell cycle arrest and growth inhibition. A previously collaborating group reported that EMF activated CAMKII, increased phospho-p38 levels associated with calcium influx in breast cancer cell line. p38 MAP kinase is known to be activated by cellular stress finally leading to cell cycle arrest or apoptosis. CAMKII is an upstream kinase of p38 that is activated by calcium influx. The authors also demonstrated that p38 MAPK pathway is activated by calcium influx induced by EMF (Sharma et al., 2019).

### 4.3 Tumor microenvironment effects

The beneficial effects of EMF on tissue regeneration and wound healing highlight their influence in all phases of the normal tissue healing process. Specifically, extremely low frequency-EMFs had demonstrated positive results for treating chronic wounds by regulating immune responses, modulate inflammatory response and promote tissue repair (Vianale et al., 2008; Patruno et al., 2010; Delle Monache et al., 2008; Gualdi et al., 2021). In normal tissue, inflammatory response functions to detect and respond to potential threats such as stress, tissue damage, infections, metabolic changes, and other disruptions to homeostasis, with the goal of restoring balance and preserving tissue function. Tissue-associated macrophages respond to changes by removing dying cells and regulating immune responses, maintaining barrier functions, and supporting stem cell growth and differentiation. Tissue inflammatory responses, initiated by local tissue macrophages and dendritic cells, can trigger the recruitment of immune cells from the bone marrow (monocytes, neutrophils, and monocyte-derived cells) and secondary lymphoid tissues (lymphoid cells). Finally, these recruited or locally expanded inflammatory cells undergo further activation, differentiation, and polarization. Those effects of EMF may explain the positive effects on mucositis observed in patients using oral agent tyrosine kinase inhibitor sorafenib in addition to EMF (Costa et al., 2011; Capareli et al., 2023).

The tumor microenvironment (TME) is a complex setting composed primarily of tumor cells, surrounding immune cells, cancer-associated fibroblasts, and vascular endothelial cells. These cellular interactions allow tumor cells to escape immune detection, driving the processes of tumor progression and metastasis. Growing evidence highlights the crucial roles of both innate immune cells (such as macrophages, neutrophils, natural killer cells, dendritic cells, and myeloid-derived suppressor cells) and adaptive immune cells (T cells and B cells) within the TME. Notably, innate immune cells actively shape their environment by releasing a variety of cytokines, chemokines, and other molecules, which influence tumor survival and growth (Kamińska et al., 2015). These cells contribute to cancer development and progression, besides their response to anticancer therapy (Hirata and Sahai, 2017).

There is limited research demonstrating the EMF effects on the TME (Bergandi et al., 2022; Sun et al., 2023). However, recent findings suggest that TTFields may also provoke an inflammatory response from TME. The mechanism behind this effect evolves focal disruptions in the nuclear envelope, resulting in the cytosolic release of large clusters of micronuclei. These micronuclei strongly recruit and activate two major DNA sensors. The activation of these sensors subsequently engages the cGAS/stimulator of interferon genes (STING) and AIM2/caspase 1 inflammasomes, leading to the production of proinflammatory cytokines, type 1 interferons (T1IFNs), and T1IFN-responsive genes. Furthermore, single-cell and bulk RNA sequencing of peripheral blood mononuclear cells from cancer patients revealed significant post-TTFields activation of adaptive immunity through a T1IFN-based pathway producing T cell activation and clonal expansion (Chen D. et al., 2022).

### 4.4 Cancer cell specificity

As previously discussed, the EMF effects on cancer growth are through mechanisms involving a number different transduction pathways (Sun et al., 2023). Research has shown that EMF exposure produces epigenetic cellular interactions and causes the release of specific substrates, thereby modifying signaling pathways and reinstating contact inhibition among tumor cells (for review see reference (Barati et al., 2021; Dieper et al., 2024)). However, relevant gene/translational activation pathway have not been consistently identified to date. The initial cellular response to extremely low-frequency EMFs exposure is ROS generation and critical alteration in intracellular Ca2+ levels, which significantly contributes to the diverse anti-proliferative and pro-apoptotic signaling observed in cells (Patergnani et al., 2020). Investigations also reveal the role of electrical signals and calcium ions, acting as secondary messengers in cell division, with fluorescent probe studies showing that membrane potentials in adherent cells tend to hyperpolarize under EMF exposure, unlike in suspended cells (Quicke et al., 2021; Catacuzzeno, Fioretti, and Franciolini, 2012; Sun et al., 2023).

In studies using conditioned media from adherent tumor cells, these cells exhibited greater vulnerability to EMF effects, indicating that particular components within the media are crucial to this susceptibility (Nezamtaheri MaryamSadat et al., 2022; Cox, Bavi, and Martinac, 2019; Alexandrova et al., 2006). Further investigations with cell cultures and xenogeneic models, involving the swap between normal and tumor cells, indicated that tumor cells experienced inhibition after exposure while normal cells remained largely unaffected (Jimenez et al., 2019; Buckner et al., 2015). These results support the idea that specific substrates responsive to EMF predominantly exist in tumor-sensitive cells explained by differences in bioelectrical properties. Furthermore, findings suggest that EMF exposure does not directly destroy cells but instead promotes antiproliferation and apoptosis over time, likely through mechanisms related to contact inhibition and epigenetic alterations.

The common sense is that although cells generally maintain physiological resilience against the solitary impacts of extremely low frequency-EMF, under stressful conditions such as hypoxic, metabolic, and oxidative stress, even a weak stressor like extremely low frequency-EMF can exert significant effects (Barati et al., 2021). Additionally, concurrent administration of chemotherapy or target agents with extremely low frequency-EMF exposure can amplify ROS levels, enhancing oxidative stress caused by chemotherapeutic drugs due to overly rapid ROS accumulation, leaving cells with insufficient adaptation time (Chen M. Y. et al., 2022; Ruiz-Gómez et al., 2002; Capareli et al., 2023). As such, extremely low frequency-EMF exposure can also trigger autophagy as a response (Roderick and Cook, 2008). Contrasting with chemotherapeutic agents, there is no consensus among investigators that extremely low frequency-EMF exposure does exhibit a clear dose-response relationship, meaning that increased EMF intensity does necessarily enhance apoptosis or other biological effects (Tuszynski and Costa, 2022). Moreover, no clear threshold exists for the induction of extremely low frequency-EMF biological effects, yet even very low electromagnetic flux densities and brief exposure durations can trigger biological responses in many experiments. Due to the limitations of existing experiments, the long-term effects at various timescales, such as on circadian rhythms, cannot be determined from the current clinical data. Further research using a larger dataset in prospective clinical trials is required to explore these long-lasting effects. However, given that the study population consists of patients with advanced incurable cancer, long-term safety is a less critical ethical consideration.

## 5 Clinical data

The clinical experience with time-variable EMF began with an early version of an EMF generator device. Initially, compassionate treatment was offered to 28 patients with various advanced cancer types (Barbault et al., 2009). Patients were exposed to a series of modulated frequencies, ranging from lowest to highest, and were cycled through every 3 s during each 60-min session. The home treatment regimen consisted of three daily sessions lasting 60 min each, continuing until tumor progression or the patient’s death, and was selected empirically. One notable case involved a patient with hormone-refractory breast cancer that had metastasized to the liver and bones; this patient achieved a partial response lasting 13.5 months.

Subsequently, a feasibility study was conducted involving 41 patients with advanced hepatocellular carcinoma (HCC) (Costa et al., 2011). These patients were exposed to the same 194 amplitude-modulated EMF frequencies ranging from 300 Hz to 20 kHz. The treatment was well-tolerated, with no recorded toxicities were reported. Notably, 14 patients (34.1%) had stable disease for more than 6 months, the median progression-free survival was 4.4 months, and the median overall survival was 6.7 months. One patient, previously part of the SHARP trial (sorafenib arm) and showing disease progression at enrollment, maintained nearly complete response for 58 months. Six patients survived longer than 24 months, with four exceeding 36 months; importantly, five of these long-term survivors had radiological evidence of disease progression at study start.

The next-generation of the EMF generator was evaluated in another feasibility study involving both healthy volunteers and patients with advanced HCC (Capareli et al., 2023). This study exposed 47 patients with HCC and 51 healthy volunteers to patient-specific, personalized active frequencies identified based on each individual’s hemodynamic response to EMF. These frequencies, including their harmonics up to 20 kHz, were administered during outpatient treatment sessions scheduled every two to 4 weeks for 90 min, until tumor progression or patient death. Among the patients, 36 underwent repeated exposures, totaling 481 sessions. EMF was used both as monotherapy (28 patients) and in combination with other therapies (19 patients). Mild somnolence was the only reported side effect during the exposures. Notably, 23% of the patients experienced tumor reduction, including four complete responses observed primarily in those undergoing monotherapy. Some patients showed tumor changes confimed by histological findings as early as 3 hours post-exposure.

After concluding the feasibility studies, a single-center compassionate access program commenced for patients with HCC, glioma, colorectal adenocarcinoma, and pancreatic carcinoma. From April 2019 to 24 April 2023, 200 patients were enrolled, including 30 with advanced HCC. A peer-reviewed manuscript detailing findings in 66 advanced HCC patients was recently published (Capareli et al., 2023). The program also included 75 patients with high-grade gliomas (e.g., glioblastoma) with 67 undergoing 613 EMF exposure procedures reported in abstract only (Costa, personal communication). Of these, 39 patients with glioblastoma received EMF alongside first-line therapies, such as radiation and temozolomide, with a median overall survival of 26.0 months. For patients with unmethylated MGMT, the median overall survival was 25.0 months, and for those with IDH wild-type status, it was 26.0 months. In the second-line treatment setting, 28 patients with glioblastoma initiated EMF in combination with salvage therapies, achieving a median overall survival of 7.9 months.

## 6 Experimental medical devices

Devices designed to expose patients to a non-thermal low-energy time-variant EMF are experimental and they are described as shortwave therapy devices with similar technological characteristics. There are two time-variant EMF devices with these characteristics in clinical testing focus on patients with advanced cancer: TheraBionic P1 (TheraBionic, Bloomfield Hills, MI) and AutEMsys (Autem Therapeutics, Hanover, NH). Those systems apply EMF centered at 27.12 MHz ± 50 ppm carrier frequency to a patient using an intraoral application by a spoon-shaped antenna (RF applicator) placed inside the mouth with the concave portion of the spoon on the tongue and the convex surface of the spoon resting on the hard palette. Both devices are capable of administering to AM at specific frequencies in the RF range from 10 Hz to 21 kHz. The AM signals (known as the envelope) can interact with biological rhythms (cyclic attractor). Those interactions are easier to perceive if the target tissue produces oscillatory electric signals such as those from the heart and brain. Those excitable cells operate at oscillatory frequencies in similar frequency range produced by the AM signals (Capareli et al., 2023). However, because of the different bioelectrical characteristics between normal and cancer cells, window of opportunity, AM signals appear to be harmless to normal cells and have detrimental effects in cancer cells.

Both devices apply a similar range of AM frequencies (10 Hz–21 KHz). Both devices operate below a safe energy level for the human body (IARC Working Group on the Evaluation of Carcinogenic Risks to Humans, 2013). TheraBionic P1 uses a fixed set of AM frequencies according to a given tumor type. AutEMsys uses a distinct set of AM frequencies for each patient based on reproducible hemodynamic variability changes using proprietary algorithms. Hemodynamic variability changes are used as a surrogate for AM frequencies with demonstrated effect in the patient’s oscillatory system. TheraBionic P1 has been tested in a home-based daily treatment schedule. AutEMsys, on the other hand, uses an out-patient clinic once every 2-week administration schedule.

The effects of exposure to the carrier wave and the envelope waves are distributed throughout the entire body thereby exerting a systemic therapeutic effect. The AutEMsys system is uniquely capable of measuring both the output energy and the energy reflected during each frequency delivered and it can estimate the levels (e.g., strength) of EMF on human tissue. The effect of EMF depends on several factors: the frequency, strength, and the tissue’s composition, including its water, fat, protein, and salt content, representing different dielectric properties of the tissues. Because the output of the AutEMsys was fine-tuned using the Computable Virtual Population V4.0 and V3.x (cViP) models, the system can estimate the levels of energy absorbed by any target region in the human body (e.g., accurate simulations and planning of energy delivery and SAR levels) (Hogan et al., 2003; Gosselin et al., 2014).

## 7 Future directions

Documenting the complex behaviors of cancer cells poses significant challenges due to the limitations of EMF signal propagation in laboratory settings, such as cell cultures and animal models. These settings often fail to fully replicate the intricate interactions and environmental conditions present in human bodies. Consequently, leveraging data from actual cancer patients is essential to gaining a comprehensive understanding of the diverse ways that these cells respond to EMF, as discussed in this manuscript. Early clinical research into the bioelectrical properties of cancer cells suggests that time-variant EMF could be a groundbreaking new modality in the systemic treatment toolbox for cancer patients. The potential advantages of this new therapeutic modality include its notable safety profile, lack of interactions with other drugs, and user-friendly application. These attributes make it an appealing option both as a single-agent anti-cancer treatment and in combination with existing therapies. We are currently developing dynamic models and equations to simulate and explain how time-varying EMFs interact at the cellular level, disrupting cancer cell processes while sparing healthy cells.

Time-variant EMF therapy has progressed to multi-site clinical trials, representing a significant step forward in its development yet in its infancy. For this reason, there is no comparative data available yet. The forthcoming research from these trials is expected to validate and expand upon the preliminary clinical findings including prospective randomized trials that will either compare this therapy to conventional treatments or combine it with them. These studies are meticulously designed to collect high-quality data that will be crucial in determining the efficacy and safety of EMF therapy in a real-world setting. The data gathered from these clinical trials will be pivotal in deciding whether this new therapeutic approach will become a standard practice in cancer treatment. If the trials confirm the preliminary positive results, time-variant EMF therapy could offer a novel, non-invasive treatment option for cancer patients. This would mark a significant shift in how we approach cancer treatment, providing a complementary method to existing therapies that could enhance patient outcomes. Future research should also explore the synergistic effects of combining time-variant EMF therapy with other treatment modalities, such as chemotherapy, immunotherapy, and radiation therapy. Understanding how EMF interacts with these treatments could lead to optimized protocols that maximize therapeutic efficacy while minimizing side effects.

To fully harness the potential of time-variant EMF therapy, further studies are needed to further elucidate the underlying mechanisms by which EMF affects cancer cells. Investigating how EMF influences cellular processes, such as signal transduction, gene expression, and metabolic pathways, will provide deeper insights into its therapeutic action and help refine treatment parameters. Assessing the long-term safety and efficacy of time-variant EMF therapy is another critical area for future research. Longitudinal studies tracking patient outcomes over extended periods will help determine the durability of treatment responses and identify any potential late-onset effects. Finally, integrating time-variant EMF therapy into the broader framework of personalized medicine could enhance its effectiveness. By tailoring EMF treatment protocols to the specific bioelectrical properties, identification of patient-specific frequencies and genetic profiles of individual patients’ tumors, clinicians can potentially achieve better therapeutic outcomes.

In summary, while the current findings are promising, extensive and rigorous clinical research is necessary to fully realize the potential of time-variant EMF therapy in cancer treatment. The insights gained from these future studies will be crucial in determining whether this innovative approach will become a mainstay in the fight against cancer. Adopting the viewpoint of network physiology, regulatory mechanisms for the coordination of interactions between organs and maintaining health can be revealed.

## Glossary

-OH: Hydroxyl radical
AC: Alternating current
AM: Amplitude modulation
ATP: Adenosine Triphosphate
AutEMsys: Autem system
Ca2+: Calcium
CAMKII: Ca 2+/calmodulin-dependent protein kinase II
cAMP: Cyclic adenosine monophosphate
CcO: Cytochrome c oxidase
Cdk: Cyclin-dependent protein kinases
cVIP: Computable Virtual Population
DC: Direct current
DNA: Deoxyribonucleic acid
ECG: Electrocardiogram
ECM: Extracellular matrix
ECS: Extracellular space
EEG: Electroencephalogram
EF: Electric fields
ELF: Extremely low-frequency
EMF: Electromagnetic fields
ERK: Extracellular signal-regulated kinase 1/2
ETC: Electron Transport Chain
FCC: USA Federal Communications Commission
GHz: Gigahertz
**H2O**_**2**_: Hydrogen peroxide
HRV: Heart rate variability
HCC: Hepatocellular carcinoma
Hz: Hertz
ICS: Intracellular space
ICW: Intercellular calcium waves
IDH: Isocitrate dehydrogenase
IR: Infrared
JNK: c-Jun N-terminal kinases
K+: Potassium
KHz: Kilohertz
kV/m: Kilovolts per meter
LF: Low-frequency
MAPK: Mitogen-activated protein kinase
MGMT: Methylated-DNA--protein-cysteine methyltransferase
MHz: Megahertz
MPT: Mitochondrial permeability transition
MRI: Magnetic resonance imaging
mROS: Mitochondrial Reactive Oxygen Species
ms: Millisecond
mT: Millitesla
mtDNA: Mitochondrial DNA
NAD+/NADH: Nicotinamide adenine dinucleotide + hydrogen
NADPH: Nicotinamide adenine dinucleotide phosphate
**O**_**2**_ **-**: Superoxide
pA: Picoampere
pRb: Retinoblastoma protein
RF: Radiofrequency
RF AM EMF: Low-energy radiofrequency amplitude modulated electromagnetic fields
RLC: Resistance (R), inductance (L), and capacitance (C)
ROS: Reactive oxygen species
RRI: R–R intervals
SAR: Specific Absorption Rate
STING: Stimulator of interferon genes
T1IFNs: Type 1 interferons
TCA: Tricarboxylic acid
THz: Terahertz
TME: Tumor microenvironment
TTFieds: Tumor Treating Fields
V: Volt or voltage
V/m: Volts per meter
VGCC: Voltage-gated calcium channels
VGSC: Voltage gated sodium channels
VLF: Very low-frequency
W/kg: Watts per kilogram
µs: Microsecond

## References

[B1] Abdul Kadir L. Stacey M. Barrett-Jolley R. (2018). Emerging roles of the membrane potential: action beyond the action potential. Front. Physiol. 9, 1661. 10.3389/fphys.2018.01661 30519193 PMC6258788

[B2] Accardi A. (2015). CELL SIGNALING. Lipids link ion channels and cancer. Science 349, 789–790. 10.1126/science.aad0874 26293939 PMC8189574

[B3] Adrian E. D. (1914). The all-or-none principle in nerve. J. Physiol. 47, 460–474. 10.1113/jphysiol.1914.sp001637 16993222 PMC1420489

[B4] Aguilar A. A. Ho M. C. Chang E. Carlson K. W. Natarajan A. Marciano T. (2021). Permeabilizing cell membranes with electric fields. Cancers (Basel) 13, 2283. 10.3390/cancers13092283 34068775 PMC8126200

[B5] Ahmad M.Al Al Natour Z. Mustafa F. Rizvi T. A. (2018). Electrical characterization of normal and cancer cells. IEEE Access 6, 25979–25986. 10.1109/access.2018.2830883

[B6] Alexandrova A. Y. Kopnin P. B. Vasiliev J. M. Kopnin B. P. (2006). ROS up-regulation mediates Ras-induced changes of cell morphology and motility. Exp. Cell Res. 312, 2066–2073. 10.1016/j.yexcr.2006.03.004 16624288

[B7] Anastassiou C. A. Montgomery S. M. Barahona M. Buzsaki G. Koch C. (2010). The effect of spatially inhomogeneous extracellular electric fields on neurons. J. Neurosci. 30, 1925–1936. 10.1523/JNEUROSCI.3635-09.2010 20130201 PMC6633973

[B8] Asim M. Amin F. El-Menyar A. (2020). Multiple organ dysfunction syndrome: contemporary insights on the clinicopathological spectrum. Qatar Med. J. 2020, 22. 10.5339/qmj.2020.22 33628712 PMC7884906

[B9] Baker S. A. Rutter J. (2023). 'Metabolites as signalling molecules. Nat. Rev. Mol. Cell Biol. 24, 355–374. 10.1038/s41580-022-00572-w 36635456

[B10] Ballini M. Müller J. Livi P. Chen Y. Frey U. Stettler A. (2014). A 1024-channel CMOS microelectrode array with 26,400 electrodes for recording and stimulation of electrogenic cells *in vitro* . IEEE J. Solid-State Circuits 49, 2705–2719. 10.1109/JSSC.2014.2359219 28502989 PMC5424881

[B11] Barati M. Darvishi B. Javidi M. A. Mohammadian A. Shariatpanahi S. P. Eisavand M. R. (2021). Cellular stress response to extremely low-frequency electromagnetic fields (ELF-EMF): an explanation for controversial effects of ELF-EMF on apoptosis. Cell Prolif. 54, e13154. 10.1111/cpr.13154 34741480 PMC8666288

[B12] Barbault A. Costa F. P. Bottger B. Munden R. F. Bomholt F. Kuster N. (2009). 'Amplitude-modulated electromagnetic fields for the treatment of cancer: discovery of tumor-specific frequencies and assessment of a novel therapeutic approach. J. Exp. Clin. Cancer Res. 28, 51. 10.1186/1756-9966-28-51 19366446 PMC2672058

[B13] Bartsch R. Ivanov P. (2014). Coexisting forms of coupling and phase-transitions in physiological networks. Nonlinear Dynamics of Electronic Systems, 270, 287. 10.1007/978-3-319-08672-9_33

[B14] Bartsch R. P. Liu K. K. Bashan A. Ivanov P.Ch (2015). Network physiology: how organ systems dynamically interact. PLoS One 10, e0142143. 10.1371/journal.pone.0142143 26555073 PMC4640580

[B15] Bashan A. Bartsch R. P. Kantelhardt J. W. Havlin S. Ivanov P.Ch (2012). Network physiology reveals relations between network topology and physiological function. Nat. Commun. 3, 702. 10.1038/ncomms1705 22426223 PMC3518900

[B16] Bassett C. A. Pawluk R. J. Pilla A. A. (1974). Augmentation of bone repair by inductively coupled electromagnetic fields. Science 184, 575–577. 10.1126/science.184.4136.575 4821958

[B17] Bergandi L. Lucia U. Grisolia G. Salaroglio I. C. Gesmundo I. Granata R. (2022). Thermomagnetic resonance effect of the extremely low frequency electromagnetic field on three-dimensional cancer models. Int. J. Mol. Sci. 23, 7955. 10.3390/ijms23147955 35887313 PMC9318636

[B18] Berner R. Sawicki J. Thiele M. Löser T. Schöll E. (2022). Critical parameters in dynamic network modeling of sepsis. Front. Netw. Physiology 2, 904480. 10.3389/fnetp.2022.904480 PMC10012967 36926088

[B19] Bertagna F. Lewis R. Silva S. R. P. McFadden J. Jeevaratnam K. (2021). Effects of electromagnetic fields on neuronal ion channels: a systematic review. Ann. N. Y. Acad. Sci. 1499, 82–103. 10.1111/nyas.14597 33945157

[B20] Bezanilla F. (2008). How membrane proteins sense voltage. Nat. Rev. Mol. Cell Biol. 9, 323–332. 10.1038/nrm2376 18354422

[B21] Binggeli R. Weinstein R. C. (1986). Membrane potentials and sodium channels: hypotheses for growth regulation and cancer formation based on changes in sodium channels and gap junctions. J. Theor. Biol. 123, 377–401. 10.1016/s0022-5193(86)80209-0 2443763

[B22] Blackman C. F. (2012). Treating cancer with amplitude-modulated electromagnetic fields: a potential paradigm shift, again? Br. J. Cancer 106, 241–242. 10.1038/bjc.2011.576 22251967 PMC3261673

[B23] Blank M. Soo L. (1998). 'Frequency dependence of cytochrome oxidase activity in magnetic fields. Bioelectrochemistry Bioenergetics 46, 139–143. 10.1016/s0302-4598(98)00126-3

[B24] Bonzanni M. Payne S. L. Adelfio M. Kaplan D. L. Levin M. Oudin M. J. (2020). Defined extracellular ionic solutions to study and manipulate the cellular resting membrane potential. Biol. Open 9, bio048553. 10.1242/bio.048553 31852666 PMC6994931

[B25] Boulware M. J. Marchant J. S. (2008). Timing in cellular Ca2+ signaling. Curr. Biol. 18, R769–R776. 10.1016/j.cub.2008.07.018 18786382 PMC3236564

[B26] Buchman T. G. (1996). Physiologic stability and physiologic state. J. Trauma 41, 599–605. 10.1097/00005373-199610000-00002 8858016

[B27] Buckman J. F. Reynolds I. J. (2001). Spontaneous changes in mitochondrial membrane potential in cultured neurons. J. Neurosci. 21, 5054–5065. 10.1523/JNEUROSCI.21-14-05054.2001 11438581 PMC6762873

[B28] Buckner C. A. Buckner A. L. Koren S. A. Persinger M. A. Lafrenie R. M. (2015). 'Inhibition of cancer cell growth by exposure to a specific time-varying electromagnetic field involves T-type calcium channels. PLoS One 10, e0124136. 10.1371/journal.pone.0124136 25875081 PMC4397079

[B29] Canolty R. T. Knight R. T. (2010). The functional role of cross-frequency coupling. Trends Cogn. Sci. 14, 506–515. 10.1016/j.tics.2010.09.001 20932795 PMC3359652

[B30] Cantero M. D. R. Villa Etchegoyen C. Perez P. L. Scarinci N. Cantiello H. F. (2018). Bundles of brain microtubules generate electrical oscillations. Sci. Rep. 8, 11899. 10.1038/s41598-018-30453-2 30093720 PMC6085364

[B31] Capareli F. Costa F. Tuszynski J. A. Sousa M. C. Setogute Y. C. Lima P. D. (2023). 'Low-energy amplitude-modulated electromagnetic field exposure: feasibility study in patients with hepatocellular carcinoma. Cancer Med. 12, 12402–12412. 10.1002/cam4.5944 37184216 PMC10278519

[B32] Caswell P. T. Vadrevu S. Norman J. C. (2009). Integrins: masters and slaves of endocytic transport. Nat. Rev. Mol. Cell Biol. 10, 843–853. 10.1038/nrm2799 19904298

[B33] Catacuzzeno L. Fioretti B. Franciolini F. (2012). A theoretical study on the role of Ca(2+)-activated K+ channels in the regulation of hormone-induced Ca2+ oscillations and their synchronization in adjacent cells. J. Theor. Biol. 309, 103–112. 10.1016/j.jtbi.2012.05.009 22659037

[B34] Cefalas A. C. Gavriil V. Ferraro A. Kollia Z. Sarantopoulou E. (2021). 'Dynamics and physics of integrin activation in tumor cells by nano-sized extracellular ligands and electromagnetic fields. Methods Mol. Biol. 2217, 197–233. 10.1007/978-1-0716-0962-0_12 33215383

[B35] Ceresoli G. L. Aerts J. G. Dziadziuszko R. Ramlau R. Cedres S. van Meerbeeck J. P. (2019). 'Tumour Treating Fields in combination with pemetrexed and cisplatin or carboplatin as first-line treatment for unresectable malignant pleural mesothelioma (STELLAR): a multicentre, single-arm phase 2 trial. Lancet Oncol. 20, 1702–1709. 10.1016/S1470-2045(19)30532-7 31628016

[B36] Cervera J. Alcaraz A. Mafe S. (2016). Bioelectrical signals and ion channels in the modeling of multicellular patterns and cancer biophysics. Sci. Rep. 6, 20403. 10.1038/srep20403 26841954 PMC4740742

[B37] Chang K. T. Niescier R. F. Min K. T. (2011). Mitochondrial matrix Ca2+ as an intrinsic signal regulating mitochondrial motility in axons. Proc. Natl. Acad. Sci. U. S. A. 108, 15456–15461. 10.1073/pnas.1106862108 21876166 PMC3174631

[B38] Charles A. (1998). Intercellular calcium waves in glia. Glia 24, 39–49. 10.1002/(sici)1098-1136(199809)24:1<39::aid-glia5>3.0.co;2-w 9700488

[B39] Chen B. Ciria L. F. Hu C. Ivanov P.Ch (2022). Ensemble of coupling forms and networks among brain rhythms as function of states and cognition. Commun. Biol. 5, 82. 10.1038/s42003-022-03017-4 35064204 PMC8782865

[B40] Chen D. Le S. B. Hutchinson T. E. Calinescu A. A. Sebastian M. Jin D. (2022). Tumor Treating Fields dually activate STING and AIM2 inflammasomes to induce adjuvant immunity in glioblastoma. J. Clin. Invest 132, e149258. 10.1172/JCI149258 35199647 PMC9012294

[B41] Chen M. Y M. Y. Li J. Zhang N. Waldorff E. I. Ryaby J. T. Fedor P. (2022). '*in vitro* and *in vivo* study of the effect of osteogenic pulsed electromagnetic fields on breast and lung cancer cells. Technol. Cancer Res. Treat. 21, 15330338221124658. 10.1177/15330338221124658 36172744 PMC9523832

[B42] Cheong R. Levchenko A. (2010). Oscillatory signaling processes: the how, the why and the where. Curr. Opin. Genet. Dev. 20, 665–669. 10.1016/j.gde.2010.08.007 20971631 PMC3895451

[B43] Chiche J. Brahimi-Horn M. C. Pouyssegur J. (2010). Tumour hypoxia induces a metabolic shift causing acidosis: a common feature in cancer. J. Cell Mol. Med. 14, 771–794. 10.1111/j.1582-4934.2009.00994.x 20015196 PMC3823111

[B44] Cimpianu C. L. Strube W. Falkai P. Palm U. Hasan A. (2017). 'Vagus nerve stimulation in psychiatry: a systematic review of the available evidence. J. Neural Transm. (Vienna) 124, 145–158. 10.1007/s00702-016-1642-2 27848034

[B45] Cone C. D. Jr. (1971). Unified theory on the basic mechanism of normal mitotic control and oncogenesis. J. Theor. Biol. 30, 151–181. 10.1016/0022-5193(71)90042-7 5555269

[B46] Cone C. D. Jr. Tongier M. Jr. (1973). Contact inhibition of division: involvement of the electrical transmembrane potential. J. Cell Physiol. 82, 373–386. 10.1002/jcp.1040820307 4590237

[B47] Conte F. Fiscon G. Licursi V. Bizzarri D. D'Antò T. Farina L. (2019). A paradigm shift in medicine: a comprehensive review of network-based approaches. Biochimica Biophysica Acta (BBA) - Gene Regul. Mech. 1863, 194416. 10.1016/j.bbagrm.2019.194416 31382052

[B48] Cornell-Bell A. H. Finkbeiner S. M. Cooper M. S. Smith S. J. (1990). Glutamate induces calcium waves in cultured astrocytes: long-range glial signaling. Science 247, 470–473. 10.1126/science.1967852 1967852

[B49] Costa F. P. de Oliveira A. C. Meirelles R. Machado M. C. Zanesco T. Surjan R. (2011). Treatment of advanced hepatocellular carcinoma with very low levels of amplitude-modulated electromagnetic fields. Br. J. Cancer 105, 640–648. 10.1038/bjc.2011.292 21829195 PMC3188936

[B50] Cox C. D. Bavi N. Martinac B. (2019). Biophysical principles of ion-channel-mediated mechanosensory transduction. Cell Rep. 29, 1–12. 10.1016/j.celrep.2019.08.075 31577940

[B51] Cui Y. Liu X. Yang T. Mei Y. A. Hu C. (2014). Exposure to extremely low-frequency electromagnetic fields inhibits T-type calcium channels via AA/LTE4 signaling pathway. Cell Calcium 55, 48–58. 10.1016/j.ceca.2013.11.002 24360572

[B268] Dahms T. Lehnert J. Schöll E. (2012). Cluster and group synchronization in delay-coupled networks. Phys. Rev. E86, 016202. 10.1103/PhysRevE.86.016202 23005502

[B52] D'Angelo J. Ritchie S. D. Oddson B. Gagnon D. D. Mrozewski T. Little J. (2023). 'Using heart rate variability methods for health-related outcomes in outdoor contexts: a scoping review of empirical studies. Int. J. Environ. Res. Public Health 20, 1330. 10.3390/ijerph20021330 36674086 PMC9858817

[B53] D'Autreaux B. Toledano M. B. (2007). ROS as signalling molecules: mechanisms that generate specificity in ROS homeostasis. Nat. Rev. Mol. Cell Biol. 8, 813–824. 10.1038/nrm2256 17848967

[B54] Davalos R. V. Mir I. L. Rubinsky B. (2005). Tissue ablation with irreversible electroporation. Ann. Biomed. Eng. 33, 223–231. 10.1007/s10439-005-8981-8 15771276

[B55] Davies P. C. Demetrius L. Tuszynski J. A. (2011). Cancer as a dynamical phase transition. Theor. Biol. Med. Model 8, 30. 10.1186/1742-4682-8-30 21867509 PMC3177875

[B56] Debir B. Meaney C. Kohandel M. Burcin Unlu M. (2021). The role of calcium oscillations in the phenotype selection in endothelial cells. Sci. Rep. 11, 23781. 10.1038/s41598-021-02720-2 34893636 PMC8664853

[B57] De Franceschi N. Hamidi H. Alanko J. Sahgal P. Ivaska J. (2015). 'Integrin traffic - the update. J. Cell Sci. 128, 839–852. 10.1242/jcs.161653 25663697 PMC4342575

[B58] Dejos C. Gkika D. Cantelmo A. R. (2020). The two-way relationship between calcium and metabolism in cancer. Front. Cell Dev. Biol. 8, 573747. 10.3389/fcell.2020.573747 33282859 PMC7691323

[B59] Delierneux C. Kouba S. Shanmughapriya S. Potier-Cartereau M. Trebak M. Hempel N. (2020). Mitochondrial calcium regulation of redox signaling in cancer. Cells 9, 432. 10.3390/cells9020432 32059571 PMC7072435

[B60] Delle Monache S. Alessandro R. Iorio R. Gualtieri G. Colonna R. (2008). 'Extremely low frequency electromagnetic fields (ELF-EMFs) induce *in vitro* angiogenesis process in human endothelial cells. Bioelectromagnetics 29, 640–648. 10.1002/bem.20430 18512694

[B61] Del Olmo M. Schmal C. Mizaikoff C. Grabe S. Gabriel C. Kramer A. (2023). Are circadian amplitudes and periods correlated? A new twist in the story. F1000Res 12, 1077. 10.12688/f1000research.135533.1 37771612 PMC10526121

[B62] De Nicolo B. Cataldi-Stagetti E. Diquigiovanni C. Bonora E. (2023). Calcium and reactive oxygen species signaling interplays in cardiac physiology and pathologies. Antioxidants (Basel) 12, 353. 10.3390/antiox12020353 36829912 PMC9952851

[B63] De Ninno A. Pregnolato M. (2017). Electromagnetic homeostasis and the role of low-amplitude electromagnetic fields on life organization. Electromagn. Biol. Med. 36, 115–122. 10.1080/15368378.2016.1194293 27399207

[B65] Di Biasio A. Cametti C. (2007). Effect of shape on the dielectric properties of biological cell suspensions. Bioelectrochemistry 71, 149–156. 10.1016/j.bioelechem.2007.03.002 17428746

[B66] Dieper A. Scheidegger S. Füchslin R. M. Veltsista P. D. Stein U. Weyland M. (2024). Literature review: potential non-thermal molecular effects of external radiofrequency electromagnetic fields on cancer. Int. J. Hyperth. 41, 2379992. 10.1080/02656736.2024.2379992 39019469

[B67] DiFrancesco D. (1986). Characterization of single pacemaker channels in cardiac sino-atrial node cells. Nature 324, 470–473. 10.1038/324470a0 2431323

[B68] Di Gregorio E. Israel S. Staelens M. Tankel G. Shankar K. Tuszynski J. A. (2022). The distinguishing electrical properties of cancer cells. Phys. Life Rev. 43, 139–188. 10.1016/j.plrev.2022.09.003 36265200

[B69] Dilger J. P. McLaughlin S. G. McIntosh T. J. Simon S. A. (1979). The dielectric constant of phospholipid bilayers and the permeability of membranes to ions. Science 206, 1196–1198. 10.1126/science.228394 228394

[B70] Dora K. A. (2001). Intercellular Ca2+ signalling: the artery wall. Semin. Cell Dev. Biol. 12, 27–35. 10.1006/scdb.2000.0214 11162744

[B71] Emmenegger V. Obien M. E. J. Franke F. Hierlemann A. (2019). 'Technologies to study action potential propagation with a focus on HD-MEAs. Front. Cell Neurosci. 13, 159. 10.3389/fncel.2019.00159 31118887 PMC6504789

[B72] Endlicher R. Drahota Z. Štefková K. Červinková Z. Kučera O. (2023). The mitochondrial permeability transition pore-current knowledge of its structure, function, and regulation, and optimized methods for evaluating its functional state. Cells 12, 1273. 10.3390/cells12091273 37174672 PMC10177258

[B269] Erneux T. (2024). Strong delayed negative feedback. Front. Netw. Physiol. 4, 1399272. 10.3389/fnetp.2024.1399272 38903729 PMC11188390

[B73] Feng B. Qiu L. Ye C. Chen L. Fu Y. Sun W. (2016). Exposure to a 50-Hz magnetic field induced mitochondrial permeability transition through the ROS/GSK-3β signaling pathway. Int. J. Radiat. Biol. 92, 148–155. 10.3109/09553002.2016.1135261 26850078

[B74] Fenwick R. B. Esteban-Martín S. Salvatella X. (2011). Understanding biomolecular motion, recognition, and allostery by use of conformational ensembles. Eur. Biophys. J. 40, 1339–1355. 10.1007/s00249-011-0754-8 22089251 PMC3222826

[B75] Ferrell J. E. Jr. (2013). Feedback loops and reciprocal regulation: recurring motifs in the systems biology of the cell cycle. Curr. Opin. Cell Biol. 25, 676–686. 10.1016/j.ceb.2013.07.007 23927869 PMC3836843

[B76] Filipovic N. Djukic T. Radovic M. Cvetkovic D. Curcic M. Markovic S. (2014). Electromagnetic field investigation on different cancer cell lines. Cancer Cell Int. 14, 84. 10.1186/s12935-014-0084-x

[B77] Force E. S. C. T. Bigger J. T. Camm A. J. Kleiger R. E. Malliani A. Moss A. J. (1996). Heart rate variability: standards of measurement, physiological interpretation, and clinical use. Eur. Heart J. 17, 354–381. 10.1093/oxfordjournals.eurheartj.a014868 8737210

[B78] Frandsen S. K. Vissing M. Gehl J. (2020). A comprehensive review of calcium electroporation -A novel cancer treatment modality. Cancers (Basel) 12, 290. 10.3390/cancers12020290 31991784 PMC7073222

[B79] Freddolino P. L. Tavazoie S. (2012). Beyond homeostasis: a predictive-dynamic framework for understanding cellular behavior. Annu. Rev. Cell Dev. Biol. 28, 363–384. 10.1146/annurev-cellbio-092910-154129 22559263

[B80] Freire M. J. Bernal-Méndez J. Pérez A. T. (2020a). The Lorentz force on ions in membrane channels of neurons as a mechanism for transcranial static magnetic stimulation. Electromagn. Biol. Med. 39, 310–315. 10.1080/15368378.2020.1793172 32666841

[B81] Freire M. J. Bernal-Méndez J. Pérez A. T. (2020b). The Lorentz force on ions in membrane channels of neurons as a mechanism for transcranial static magnetic stimulation. Electromagn. Biol. Med. 39, 310–315. 10.1080/15368378.2020.1793172 32666841

[B82] Friesen D. E. Baracos V. E. Tuszynski J. A. (2015). Modeling the energetic cost of cancer as a result of altered energy metabolism: implications for cachexia. Theor. Biol. Med. Model. 12, 17. 10.1186/s12976-015-0015-0 26370269 PMC4570294

[B83] Fujiwara K. Oogane M. Kanno A. Imada M. Jono J. Terauchi T. (2018). Magnetocardiography and magnetoencephalography measurements at room temperature using tunnel magneto-resistance sensors. Appl. Phys. Express 11, 023001. 10.7567/apex.11.023001

[B84] Funk R. H. Monsees T. Ozkucur N. (2009). Electromagnetic effects - from cell biology to medicine. Prog. Histochem Cytochem 43, 177–264. 10.1016/j.proghi.2008.07.001 19167986

[B85] Gaspers L. D. Thomas A. P. (2005). Calcium signaling in liver. Cell Calcium 38, 329–342. 10.1016/j.ceca.2005.06.009 16139354

[B86] Gérard C. Goldbeter A. (2012). The cell cycle is a limit cycle. Math. Model. Nat. Phenom. 7, 126–166. 10.1051/mmnp/20127607

[B87] Gerster M. Berner R. Sawicki J. Zakharova A. Škoch A. Hlinka J. (2020). FitzHugh-Nagumo oscillators on complex networks mimic epileptic-seizure-related synchronization phenomena. Chaos 30, 123130. 10.1063/5.0021420 33380049

[B88] Gherardini L. Ciuti G. Tognarelli S. Cinti C. (2014). Searching for the perfect wave: the effect of radiofrequency electromagnetic fields on cells. Int. J. Mol. Sci. 15, 5366–5387. 10.3390/ijms15045366 24681584 PMC4013569

[B89] Ghomsi P. G. Kakmeni F. M. Tchawoua C. Kofane T. C. (2015). Synchronization of cells with activator-inhibitor pathways through adaptive environment-mediated coupling. Phys. Rev. E Stat. Nonlin Soft Matter Phys. 92, 052911. 10.1103/PhysRevE.92.052911 26651766

[B90] Giaume C. Venance L. (1998). 'Intercellular calcium signaling and gap junctional communication in astrocytes. Glia 24, 50–64. 10.1002/(sici)1098-1136(199809)24:1<50::aid-glia6>3.3.co;2-z 9700489

[B91] Gibson W. Wand B. M. O'Connell N. E. (2017). Transcutaneous electrical nerve stimulation (TENS) for neuropathic pain in adults. Cochrane Database Syst. Rev. 9, Cd011976. 10.1002/14651858.CD011976.pub2 28905362 PMC6426434

[B92] Glass L. (2001). Synchronization and rhythmic processes in physiology. Nature 410, 277–284. 10.1038/35065745 11258383

[B93] Goldberger J. J. Challapalli S. Tung R. Parker M. A. Kadish A. H. (2001). 'Relationship of heart rate variability to parasympathetic effect. Circulation 103, 1977–1983. 10.1161/01.cir.103.15.1977 11306527

[B94] Gong X. Chen Z. Hu J. J. Liu C. (2022). Advances of electroporation-related therapies and the synergy with immunotherapy in cancer treatment. Vaccines (Basel) 10, 1942. 10.3390/vaccines10111942 36423037 PMC9692484

[B95] Gordon G. A. (2007). Designed electromagnetic pulsed therapy: clinical applications. J. Cell Physiol. 212, 579–582. 10.1002/jcp.21025 17577213

[B96] Görlach A. Bertram K. Hudecova S. Krizanova O. (2015). Calcium and ROS: a mutual interplay. Redox Biol. 6, 260–271. 10.1016/j.redox.2015.08.010 26296072 PMC4556774

[B97] Gosselin M. C. Neufeld E. Moser H. Huber E. Farcito S. Gerber L. (2014). 'Development of a new generation of high-resolution anatomical models for medical device evaluation: the Virtual Population 3.0. Phys. Med. Biol. 59, 5287–5303. 10.1088/0031-9155/59/18/5287 25144615

[B98] Gualdi G. Costantini E. Reale M. Amerio P. (2021). Wound repair and extremely low frequency-electromagnetic field: insight from *in vitro* study and potential clinical application. Int. J. Mol. Sci. 22, 5037. 10.3390/ijms22095037 34068809 PMC8126245

[B99] Hajnóczky G. Robb-Gaspers L. D. Seitz M. B. Thomas A. P. (1995). Decoding of cytosolic calcium oscillations in the mitochondria. Cell 82, 415–424. 10.1016/0092-8674(95)90430-1 7634331

[B100] Hales C. G. (2014). The origins of the brain's endogenous electromagnetic field and its relationship to provision of consciousness. J. Integr. Neurosci. 13, 313–361. 10.1142/S0219635214400056 25012714

[B101] Han K. S. Guo C. Chen C. H. Witter L. Osorno T. Regehr W. G. (2018). Ephaptic coupling promotes synchronous firing of cerebellar purkinje cells. Neuron 100, 564–578.e3. 10.1016/j.neuron.2018.09.018 30293822 PMC7513896

[B102] Hanahan D. Weinberg R. A. (2011). 'Hallmarks of cancer: the next generation. Cell 144, 646–674. 10.1016/j.cell.2011.02.013 21376230

[B103] Hart F. X. (2006). Integrins may serve as mechanical transducers for low-frequency electric fields. Bioelectromagnetics 27, 505–508. 10.1002/bem.20236 16715526

[B104] Hausdorff J. M. Ashkenazy Y. Peng C. K. Ivanov P. C. Stanley H. E. Goldberger A. L. (2001). When human walking becomes random walking: fractal analysis and modeling of gait rhythm fluctuations. Phys. A 302, 138–147. 10.1016/s0378-4371(01)00460-5 12033228

[B105] Heltberg M. L. Krishna S. Kadanoff L. P. Jensen M. H. (2021). A tale of two rhythms: locked clocks and chaos in biology. Cell Syst. 12, 291–303. 10.1016/j.cels.2021.03.003 33887201

[B106] Hirata E. Sahai E. (2017). Tumor microenvironment and differential responses to therapy. Cold Spring Harb. Perspect. Med. 7, a026781. 10.1101/cshperspect.a026781 28213438 PMC5495051

[B107] Hogan P. G. Chen L. Nardone J. Rao A. (2003). Transcriptional regulation by calcium, calcineurin, and NFAT. Genes Dev. 17, 2205–2232. 10.1101/gad.1102703 12975316

[B108] Horio T. Murata T. (2014). The role of dynamic instability in microtubule organization. Front. Plant Sci. 5, 511. 10.3389/fpls.2014.00511 25339962 PMC4188131

[B109] Hrabetova S. Cognet L. Rusakov D. A. Nagerl U. V. (2018). Unveiling the extracellular space of the brain: from super-resolved microstructure to *in vivo* function. J. Neurosci. 38, 9355–9363. 10.1523/JNEUROSCI.1664-18.2018 30381427 PMC6706003

[B110] Huber R. Schuderer J. Graf T. Jutz K. Borbely A. A. Kuster N. (2003). 'Radio frequency electromagnetic field exposure in humans: estimation of SAR distribution in the brain, effects on sleep and heart rate. Bioelectromagnetics 24, 262–276. 10.1002/bem.10103 12696086

[B111] Hunt T. Schooler J. W. (2019). The easy part of the hard problem: a resonance theory of consciousness. Front. Hum. Neurosci. 13, 378. 10.3389/fnhum.2019.00378 31736728 PMC6834646

[B112] Hunter D. R. Haworth R. A. Southard J. H. (1976). 'Relationship between configuration, function, and permeability in calcium-treated mitochondria. J. Biol. Chem. 251, 5069–5077. 10.1016/s0021-9258(17)33220-9 134035

[B113] IARC Working Group on the Evaluation of Carcinogenic Risks to Humans (2013). Non-ionizing radiation, Part 2: radiofrequency electromagnetic fields. IARC Monogr. Eval. Carcinog. Risks Hum. 102, 1–460. 24772662 PMC4780878

[B114] Isakovic J. Dobbs-Dixon I. Chaudhury D. Mitrecic D. (2018). Modeling of inhomogeneous electromagnetic fields in the nervous system: a novel paradigm in understanding cell interactions, disease etiology and therapy. Sci. Rep. 8, 12909. 10.1038/s41598-018-31054-9 30150694 PMC6110729

[B115] Ivanov P. C. (2021). The new field of network physiology: building the human physiolome. Front. Netw. Physiol. 1, 711778. 10.3389/fnetp.2021.711778 36925582 PMC10013018

[B116] Ivanov P.Ch. Nunes Amaral L. A. Goldberger A. L. Stanley H. E. (1998). Stochastic feedback and the regulation of biological rhythms. Europhys. Lett. 43, 363–368. 10.1209/epl/i1998-00366-3 11542723

[B117] Ivanov P. C. Rosenblum M. G. Peng C. K. Mietus J. Havlin S. Stanley H. E. (1996). 'Scaling behaviour of heartbeat intervals obtained by wavelet-based time-series analysis. Nature 383, 323–327. 10.1038/383323a0 8848043

[B118] Jensen M. H. Krishna S. (2012). 'Inducing phase-locking and chaos in cellular oscillators by modulating the driving stimuli. FEBS Lett. 586, 1664–1668. 10.1016/j.febslet.2012.04.044 22673576

[B119] Jiménez A. Lu Y. Jambhekar A. Lahav G. (2022). 'Principles, mechanisms and functions of entrainment in biological oscillators. Interface Focus 12, 20210088. 10.1098/rsfs.2021.0088 35450280 PMC9010850

[B120] Jimenez H. Blackman C. Lesser G. Debinski W. Chan M. Sharma S. (2018). 'Use of non-ionizing electromagnetic fields for the treatment of cancer. Front. Biosci. Landmark Ed. 23, 284–297. 10.2741/4591 28930547

[B121] Jimenez H. Wang M. Sharma S. Herpai D. Lesser G. Debinski W. (2016). Exth-41. The anti-proliferative effects of rf emf amplitude-modulated (am rf emf) at tumor specific frequencies on glioblastoma cells. Jpn. Soc. Neuro-Oncology 18, vi68. 10.1093/neuonc/now212.285

[B122] Jimenez H. Wang M. Zimmerman J. W. Pennison M. J. Sharma S. Surratt T. (2019). 'Tumour-specific amplitude-modulated radiofrequency electromagnetic fields induce differentiation of hepatocellular carcinoma via targeting Ca(v)3.2 T-type voltage-gated calcium channels and Ca(2+) influx. EBioMedicine 44, 209–224. 10.1016/j.ebiom.2019.05.034 31160272 PMC6604666

[B123] Kadji H. G. E. Orou J. B. C. Yamapi R. Woafo P. (2007). Nonlinear dynamics and strange attractors in the biological system. Chaos, Solit. and Fractals 32, 862–882. 10.1016/j.chaos.2005.11.063

[B124] Kaestner L. Wang X. Hertz L. Bernhardt I. (2018). Voltage-activated ion channels in non-excitable cells-A viewpoint regarding their physiological justification. Front. Physiol. 9, 450. 10.3389/fphys.2018.00450 29755371 PMC5934782

[B125] Kamińska K. Szczylik C. Bielecka Z. F. Bartnik E. Porta C. Lian F. (2015). The role of the cell-cell interactions in cancer progression. J. Cell Mol. Med. 19, 283–296. 10.1111/jcmm.12408 25598217 PMC4407603

[B126] Keating S. T. El-Osta A. (2023). Metaboloepigenetics in cancer, immunity, and cardiovascular disease. Cardiovasc Res. 119, 357–370. 10.1093/cvr/cvac058 35389425 PMC10064843

[B127] Kivrak E. G. Yurt K. K. Kaplan A. A. Alkan I. Altun G. (2017). Effects of electromagnetic fields exposure on the antioxidant defense system. J. Microsc. Ultrastruct. 5, 167–176. 10.1016/j.jmau.2017.07.003 30023251 PMC6025786

[B128] Kochen M. A. Andrews S. S. Wiley H. S. Feng S. Sauro H. M. (2022). 'Dynamics and sensitivity of signaling pathways. Curr. Pathobiol. Rep. 10, 11–22. 10.1007/s40139-022-00230-y 36969954 PMC10035447

[B129] Kumai T. (2017). Isn't there an inductance factor in the plasma membrane of nerves? Biophys. Physicobiol 14, 147–152. 10.2142/biophysico.14.0_147 28989835 PMC5627987

[B130] Kutuk T. Atak E. La Rosa A. Kotecha R. Mehta M. P. Chuong M. D. (2023). 'Tumor treating fields: narrative review of a promising treatment modality for cancer. Chin. Clin. Oncol. 12, 64. 10.21037/cco-23-82 37953242

[B131] Lai H. Levitt B. B. (2023). Cellular and molecular effects of non-ionizing electromagnetic fields. Rev. Environ. Health 39, 519–529. 10.1515/reveh-2023-0023 37021652

[B132] Langthaler S. Šajić J. L. Rienmüller T. Weinberg S. H. Christian B. (2022). Ion Channel modeling beyond state of the art: a comparison with a system theory-based model of the shaker-related voltage-gated potassium channel Kv1.1. Cells 11, 239. 10.3390/cells11020239 35053355 PMC8773569

[B133] Larsen E. R. Licht R. W. Nielsen R. E. Lolk A. Borck B. Sørensen C. (2020). Transcranial pulsed electromagnetic fields for treatment-resistant depression: a multicenter 8-week single-arm cohort study. Eur. Psychiatry 63, e18. 10.1192/j.eurpsy.2020.3 32093804 PMC7315871

[B134] Laszlo A. Ladányi M. Boda K. Csicsman J. Bari F. Serester A. (2017). Effects of extremely low frequency electromagnetic fields on turkeys. Poult. Sci. 97, 634–642. 10.3382/ps/pex304 29077912

[B135] Leal T. Kotecha R. Ramlau R. Zhang L. Milanowski J. Cobo M. (2023). Tumor Treating Fields therapy with standard systemic therapy versus standard systemic therapy alone in metastatic non-small-cell lung cancer following progression on or after platinum-based therapy (LUNAR): a randomised, open-label, pivotal phase 3 study. Lancet Oncol. 24, 1002–1017. 10.1016/S1470-2045(23)00344-3 37657460

[B136] Lehnertz K. Ansmann G. Bialonski S. Dickten H. Geier C. Porz S. (2013). Evolving networks in the human epileptic brain. Phys. D. Nonlinear Phenom. 267, 7–15. 10.1016/j.physd.2013.06.009

[B137] Levic D. S. Bagnat M. (2022). 'Self-organization of apical membrane protein sorting in epithelial cells. FEBS J. 289, 659–670. 10.1111/febs.15882 33864720 PMC8522177

[B138] Levin M. (2021). Bioelectrical approaches to cancer as a problem of the scaling of the cellular self. Prog. Biophys. Mol. Biol. 165, 102–113. 10.1016/j.pbiomolbio.2021.04.007 33961843

[B139] Lewczuk B. Redlarski G. Zak A. Ziółkowska N. Przybylska-Gornowicz B. Krawczuk M. (2014). Influence of electric, magnetic, and electromagnetic fields on the circadian system: current stage of knowledge. Biomed. Res. Int. 2014, 169459. 10.1155/2014/169459 25136557 PMC4130204

[B140] Leybaert L. Sanderson M. J. (2012). 'Intercellular Ca(2+) waves: mechanisms and function. Physiol. Rev. 92, 1359–1392. 10.1152/physrev.00029.2011 22811430 PMC4496049

[B141] Li C. Wang J. (2014). Quantifying the underlying landscape and paths of cancer. J. R. Soc. Interface 11, 20140774. 10.1098/rsif.2014.0774 25232051 PMC4191109

[B142] Liberti M. V. Locasale J. W. (2016). The Warburg effect: how does it benefit cancer cells? Trends Biochem. Sci. 41, 211–218. 10.1016/j.tibs.2015.12.001 26778478 PMC4783224

[B143] Limansky J. Samosyuk I. Chukhraev N. Zukow W. (2014). Concept of electromagnetic homeostasis and theoretical rationale for the use of low-intensity electromagnetic fields in clinical practice. Acta. Balneol. tom. LIV N. R.

[B144] Lin A. Liu K. K. L. Bartsch R. P. Ivanov P.Ch (2020). Dynamic network interactions among distinct brain rhythms as a hallmark of physiologic state and function. Commun. Biol. 3, 197. 10.1038/s42003-020-0878-4 32341420 PMC7184753

[B270] Lindner B. García-Ojalvo J. Neiman A. B. Schimansky-Geier L. (2004). Effects of noise in excitable systems. Phys. Rep. 392, 321.

[B145] Liu K. K. L. Bartsch R. P. Lin A. Mantegna R. N. Ivanov P.Ch. (2015). Plasticity of brain wave network interactions and evolution across physiologic states. Front. Neural Circuits 9, 62. 10.3389/fncir.2015.00062 26578891 PMC4620446

[B146] Liu Y. Liu W. B. Liu K. J. Ao L. Cao J. Zhong J. L. (2016). Overexpression of miR-26b-5p regulates the cell cycle by targeting CCND2 in GC-2 cells under exposure to extremely low frequency electromagnetic fields. Cell Cycle 15, 357–367. 10.1080/15384101.2015.1120924 26637059 PMC4943694

[B147] Loughran S. P. McKenzie R. J. Jackson M. L. Howard M. E. Croft R. J. (2012). Individual differences in the effects of mobile phone exposure on human sleep: rethinking the problem. Bioelectromagnetics 33, 86–93. 10.1002/bem.20691 21812009

[B148] Mackey M. C. Glass L. (1977). 'Oscillation and chaos in physiological control systems. Science 197, 287–289. 10.1126/science.267326 267326

[B149] Marino A. A. Morris D. M. Schwalke M. A. Iliev I. G. Rogers S. (1994). Electrical potential measurements in human breast cancer and benign lesions. Tumour Biol. 15, 147–152. 10.1159/000217885 8073228

[B150] Mattsson M. O. Simkó M. (2019). 'Emerging medical applications based on non-ionizing electromagnetic fields from 0 Hz to 10 THz. Med. Devices (Auckl) 12, 347–368. 10.2147/MDER.S214152 31565000 PMC6746309

[B151] McCaig C. D. Rajnicek A. M. Song B. Zhao M. (2005). 'Controlling cell behavior electrically: current views and future potential. Physiol. Rev. 85, 943–978. 10.1152/physrev.00020.2004 15987799

[B152] McCormack J. G. Denton R. M. (1993). Mitochondrial Ca2+ transport and the role of intramitochondrial Ca2+ in the regulation of energy metabolism. Dev. Neurosci. 15, 165–173. 10.1159/000111332 7805568

[B153] McEwen B. S. Wingfield J. C. (2010). 'What is in a name? Integrating homeostasis, allostasis and stress. Horm. Behav. 57, 105–111. 10.1016/j.yhbeh.2009.09.011 19786032 PMC2815096

[B154] Mehdizadeh R. Madjid Ansari A. Forouzesh F. Shahriari F. Shariatpanahi S. P. Salaritabar A. (2023). 'P53 status, and G2/M cell cycle arrest, are determining factors in cell-death induction mediated by ELF-EMF in glioblastoma. Sci. Rep. 13, 10845. 10.1038/s41598-023-38021-z 37407632 PMC10323118

[B155] Misek J. Veterník M. Tonhajzerova I. Jakusova V. Janousek L. Jakus J. (2020). Radiofrequency electromagnetic field affects heart rate variability in rabbits. Physiol. Res. 69, 633–643. 10.33549/physiolres.934425 32672045 PMC8549896

[B156] Moraveji M. Haghighipour N. Keshvari H. Abbariki T. N. Ali Shokrgozar M. Amanzadeh A. (2016). Effect of extremely low frequency electromagnetic field on MAP2 and nestin gene expression of hair follicle dermal papilla cells. Int. J. Artif. Organs 39, 294–299. 10.5301/ijao.5000512 27515859

[B157] Moreddu R. (2024). Nanotechnology and cancer bioelectricity: bridging the gap between biology and translational medicine. Adv. Sci. (Weinh) 11, e2304110. 10.1002/advs.202304110 37984883 PMC10767462

[B158] Mousavi Maleki N. S. Entezari M. Abdi S. Tekiyehmaroof N. (2022). Electromagnetic fields change the expression of suppressor of cytokine signaling 3 (SOCS3) and cathepsin L2 (CTSL2) genes in adenocarcinoma gastric (AGS) cell line. Int. J. Cancer Manag. 15, e117270. 10.5812/ijcm-117270

[B159] Murphy M. P. (2009). How mitochondria produce reactive oxygen species. Biochem. J. 417, 1–13. 10.1042/BJ20081386 19061483 PMC2605959

[B160] Nakamura H. Takada K. (2021). Reactive oxygen species in cancer: current findings and future directions. Cancer Sci. 112, 3945–3952. 10.1111/cas.15068 34286881 PMC8486193

[B161] Nakata T. Terada S. Hirokawa N. (1998). 'Visualization of the dynamics of synaptic vesicle and plasma membrane proteins in living axons. J. Cell Biol. 140, 659–674. 10.1083/jcb.140.3.659 9456325 PMC2140163

[B162] Nasir N. Mahmoud Al A. (2020). Cells electrical characterization: dielectric properties, mixture, and modeling theories. J. Eng. 2020, 9475490. 10.1155/2020/9475490

[B163] Nath M. Maity S. Avlani S. Weigand S. Sen S. (2021). 'Inter-body coupling in electro-quasistatic human body communication: theory and analysis of security and interference properties. Sci. Rep. 11, 4378. 10.1038/s41598-020-79788-9 33623092 PMC7902665

[B164] Nathanson M. H. Burgstahler A. D. Mennone A. Fallon M. B. Gonzalez C. B. Saez J. C. (1995). 'Ca2+ waves are organized among hepatocytes in the intact organ. Am. J. Physiol. 269, G167–G171. 10.1152/ajpgi.1995.269.1.G167 7631796

[B165] Nawarathna D. Miller J. H. Jr. Claycomb J. R. Cardenas G. Warmflash D. (2005). Harmonic response of cellular membrane pumps to low frequency electric fields. Phys. Rev. Lett. 95, 158103. 10.1103/PhysRevLett.95.158103 16241766

[B166] Nezamtaheri M. S. Goliaei B. Shariatpanahi S. P. Ansari A. M. (2022a). Differential biological responses of adherent and non-adherent (cancer and non-cancerous) cells to variable extremely low frequency magnetic fields. Sci. Rep. 12, 14225. 10.1038/s41598-022-18210-y 35987807 PMC9392794

[B167] Nezamtaheri M. S. Goliaei B. Shariatpanahi S. P. Ansari A. M. (2022b). Differential biological responses of adherent and non-adherent (cancer and non-cancerous) cells to variable extremely low frequency magnetic fields. Sci. Rep. 12, 14225. 10.1038/s41598-022-18210-y 35987807 PMC9392794

[B168] Nicola W. Hellyer P. J. Campbell S. A. Clopath C. (2018). Chaos in homeostatically regulated neural systems. Chaos 28, 083104. 10.1063/1.5026489 30180641 PMC6684369

[B169] Nijhout H. F. Reed M. C. (2014). 'Homeostasis and dynamic stability of the phenotype link robustness and plasticity. Integr. Comp. Biol. 54, 264–275. 10.1093/icb/icu010 24729131

[B170] Oizumi M. Albantakis L. Tononi G. (2014). From the phenomenology to the mechanisms of consciousness: integrated Information Theory 3.0. PLoS Comput. Biol. 10, e1003588. 10.1371/journal.pcbi.1003588 24811198 PMC4014402

[B171] Olaya-Castro A. Nazir A. Fleming G. R. (2012). Quantum-coherent energy transfer: implications for biology and new energy technologies. Philos. Trans. A Math. Phys. Eng. Sci. 370, 3613–3617. 10.1098/rsta.2012.0192 22753815 PMC3385675

[B172] O'Sullivan M. J. Lindsay A. J. (2020). The endosomal recycling pathway-at the crossroads of the cell. Int. J. Mol. Sci. 21, 6074. 10.3390/ijms21176074 32842549 PMC7503921

[B173] Pall M. L. (2013). Electromagnetic fields act via activation of voltage-gated calcium channels to produce beneficial or adverse effects. J. Cell Mol. Med. 17, 958–965. 10.1111/jcmm.12088 23802593 PMC3780531

[B174] Panagopoulos D. J. Johansson O. Carlo G. L. (2015). Polarization: a key difference between man-made and natural electromagnetic fields, in regard to biological activity. Sci. Rep. 5, 14914. 10.1038/srep14914 26456585 PMC4601073

[B175] Patergnani S. Danese A. Bouhamida E. Aguiari G. Previati M. Pinton P. (2020). 'Various aspects of calcium signaling in the regulation of apoptosis, autophagy, cell proliferation, and cancer. Int. J. Mol. Sci. 21, 8323. 10.3390/ijms21218323 33171939 PMC7664196

[B176] Patke A. Young M. W. Axelrod S. (2020). Molecular mechanisms and physiological importance of circadian rhythms. Nat. Rev. Mol. Cell Biol. 21, 67–84. 10.1038/s41580-019-0179-2 31768006

[B177] Patruno A. Amerio P. Pesce M. Vianale G. Di Luzio S. Tulli A. (2010). Extremely low frequency electromagnetic fields modulate expression of inducible nitric oxide synthase, endothelial nitric oxide synthase and cyclooxygenase‐2 in the human keratinocyte cell line HaCat: potential therapeutic effects in wound healing. Br. J. Dermatology 162, 258–266. 10.1111/j.1365-2133.2009.09527.x 19799606

[B178] Phillips J. L. (1993). Effects of electromagnetic field exposure on gene transcription. J. Cell Biochem. 51, 381–386. 10.1002/jcb.2400510401 8496241

[B179] Pilla A. Fitzsimmons R. Muehsam D. Wu J. Rohde C. Casper D. (2011). Electromagnetic fields as first messenger in biological signaling: application to calmodulin-dependent signaling in tissue repair. Biochim. Biophys. Acta 1810, 1236–1245. 10.1016/j.bbagen.2011.10.001 22005645

[B180] Pincus S. M. (1994). 'Greater signal regularity may indicate increased system isolation. Math. Biosci. 122, 161–181. 10.1016/0025-5564(94)90056-6 7919665

[B181] Piszczek P. Wójcik-Piotrowicz K. Guzdek P. Gil K. Kaszuba-Zwoińska J. (2022). Protein expression changes during phagocytosis influenced by low-frequency electromagnetic field exposure. Int. J. Biol. Macromol. 217, 481–491. 10.1016/j.ijbiomac.2022.07.080 35841960

[B182] Pokorný J. Pokorný J. Kobilková J. Jandová A. Holaj R. (2020). Cancer development and damped electromagnetic activity. Appl. Sci. 10, 1826. 10.3390/app10051826

[B183] Priel A. Tuszynski J. A. Woolf N. J. (2005). 'Transitions in microtubule C-termini conformations as a possible dendritic signaling phenomenon. Eur. Biophys. J. 35, 40–52. 10.1007/s00249-005-0003-0 16184388

[B184] Pullar C. E. (2016). The physiology of bioelectricity in development, tissue regeneration, and cancer.

[B185] Quicke P. Sun Y. Arias-Garcia M. Acker C. D. Djamgoz M. B. A. Bakal C. (2021). Membrane voltage fluctuations in human breast cancer cells. bioRxiv 2021. 10.1101/2021.12.20.473148

[B186] Quicke P. Sun Y. Arias-Garcia M. Beykou M. Acker C. D. Djamgoz M. B. A. (2022). Voltage imaging reveals the dynamic electrical signatures of human breast cancer cells. Commun. Biol. 5, 1178. 10.1038/s42003-022-04077-2 36369329 PMC9652252

[B187] Rafat M. Wheatland M. S. Bedding T. R. (2008). Dynamics of a double pendulum with distributed mass. arXiv Comput. Phys. 10.1119/1.3052072

[B188] Rao V. R. Perez-Neut M. Kaja S. Gentile S. (2015). 'Voltage-gated ion channels in cancer cell proliferation. Cancers (Basel) 7, 849–875. 10.3390/cancers7020813 26010603 PMC4491688

[B189] Rassi E. Dorffner G. Gruber W. Schabus M. Klimesch W. (2019). Coupling and decoupling between brain and body oscillations. Neurosci. Lett. 711, 134401. 10.1016/j.neulet.2019.134401 31349018

[B190] Ravin R. Cai T. X. Pursley R. H. Garmendia-Cedillos M. Pohida T. Freidlin R. Z. (2020). A novel *in vitro* device to deliver induced electromagnetic fields to cell and tissue cultures. Biophysical J. 119, 2378–2390. 10.1016/j.bpj.2020.11.002 PMC7822734 33189686

[B191] Razek A. (2022). Biological and medical disturbances due to exposure to fields emitted by electromagnetic energy devices—a review. Energies 15, 4455. 10.3390/en15124455

[B192] Ribeiro M. Elghajiji A. Fraser S. P. Burke Z. D. Tosh D. Djamgoz M. B. A. (2020). Human breast cancer cells demonstrate electrical excitability. Front. Neurosci. 14, 404. 10.3389/fnins.2020.00404 32425751 PMC7204841

[B193] Rietman E. A. Friesen D. E. Hahnfeldt P. Gatenby R. Hlatky L. Tuszynski J. A. (2013). An integrated multidisciplinary model describing initiation of cancer and the Warburg hypothesis. Theor. Biol. Med. Model 10, 39. 10.1186/1742-4682-10-39 23758735 PMC3689044

[B194] Robert W. (2022). Advanced classical electromagnetism. Princeton University Press.

[B195] Robinson K. R. Messerli M. A. (2003). 'Left/right, up/down: the role of endogenous electrical fields as directional signals in development, repair and invasion. Bioessays 25, 759–766. 10.1002/bies.10307 12879446

[B196] Roderick H. L. Cook S. J. (2008). 'Ca2+ signalling checkpoints in cancer: remodelling Ca2+ for cancer cell proliferation and survival. Nat. Rev. Cancer 8, 361–375. 10.1038/nrc2374 18432251

[B197] Roger S. Gillet L. Le Guennec J. Y. Besson P. (2015). Voltage-gated sodium channels and cancer: is excitability their primary role? Front. Pharmacol. 6, 152. 10.3389/fphar.2015.00152 26283962 PMC4518325

[B272] Rosenblum M. (2024). . Feedback control of collective dynamics in an oscillator population with time-dependent connectivity. Front. Netw. Physiol. 4, 1358146. 10.3389/fnetp.2024.135814 38371453 PMC10869593

[B271] Rosenblum M. Pikovsky A. Kurths J. (2001). Synchronization—a universal concept in nonlinear sciences. Cambridge University Press.

[B198] Ross C. L. Syed I. Smith T. L. Harrison B. S. (2017). The regenerative effects of electromagnetic field on spinal cord injury. Electromagn. Biol. Med. 36, 74–87. 10.3109/15368378.2016.1160408 27398987

[B199] Ruiz-Gómez M. J. de la Peña L. Prieto-Barcia M. I. Pastor J. M. Gil L. Martínez-Morillo M. (2002). Influence of 1 and 25 Hz, 1.5 mT magnetic fields on antitumor drug potency in a human adenocarcinoma cell line. Bioelectromagnetics 23, 578–585. 10.1002/bem.10054 12395412

[B200] Sahu S. Ghosh S. Fujita D. Bandyopadhyay A. (2014). Live visualizations of single isolated tubulin protein self-assembly via tunneling current: effect of electromagnetic pumping during spontaneous growth of microtubule. Sci. Rep. 4, 7303. 10.1038/srep07303 25466883 PMC4252892

[B201] Salazar C. Politi A. Z. Höfer T. (2008). Decoding of calcium oscillations by phosphorylation cycles: analytic results. Biophysical J. 94, 1203–1215. 10.1529/biophysj.107.113084 PMC2212700 17921221

[B202] Saliev T. Begimbetova D. Masoud A.-R. Matkarimov B. (2019). 'Biological effects of non-ionizing electromagnetic fields: two sides of a coin. Prog. Biophysics Mol. Biol. 141, 25–36. 10.1016/j.pbiomolbio.2018.07.009 30030071

[B203] Sandblom J. Galvanovskis J. (2000). Electromagnetic field absorption in stochastic cellular systems: enhanced signal detection in ion channels and calcium oscillators. Chaos, Solit. and Fractals 11, 1905–1911. 10.1016/s0960-0779(99)00127-7

[B204] Sawicki J. Berner R. Löser T. Schöll E. (2022). Modeling tumor disease and sepsis by networks of adaptively coupled phase oscillators. Front. Netw. Physiology 1, 730385. 10.3389/fnetp.2021.730385 PMC10013027 36925568

[B205] Sawicki J. Schöll E. (2024). Interplay of synchronization and cortical input in models of brain networks. Europhys. Lett. 146, 41001. 10.1209/0295-5075/ad438e

[B206] Scemes E. Giaume C. (2006). 'Astrocyte calcium waves: what they are and what they do. Glia 54, 716–725. 10.1002/glia.20374 17006900 PMC2605018

[B207] Schmal C. Hong S. Tokuda I. T. Myung J. (2022). Editorial: coupling in biological systems: definitions, mechanisms, and implications. Front. Netw. Physiol. 2, 1076702. 10.3389/fnetp.2022.1076702 36926102 PMC10012964

[B208] Scholkmann F. (2015). Two emerging topics regarding long-range physical signaling in neurosystems: membrane nanotubes and electromagnetic fields. J. Integr. Neurosci. 14, 135–153. 10.1142/S0219635215300115 25962399

[B209] Schöll E. (2022). Editorial: Network physiology, insights in dynamical systems: 2021. Front. Netw. Physiology 2, 961339. 10.3389/fnetp.2022.961339 PMC10013025 36926056

[B264] Schöll E. Hiller G. Hövel P. Dahlem M. A. (2009). Time-delayed feedback in neurosystems. Phil. Trans. R. Soc. 367, 1079 10.1098/rsta.2008.0258 19218152

[B265] Schöll E. Klapp S. H. L. Hövel P. (2016). Control of self-organizing nonlinear systems, Springer, Berlin.

[B266] Schöll E. (2020). Chimeras in physics and biology: synchronization and desynchronization of rhythms. Nova Acta Leopoldina 425, 67.

[B267] Schöll E. (2021). Partial synchronization patterns in brain networks. Europhys. Lett. 136, 18001.

[B263] Schroeder C. E. Lakatos P. (2009). The gamma oscillation: master or slave?. Brain Topogr. 22 (1), 24–26. 10.1007/s10548-009-0080-y 19205863 PMC2989849

[B210] Schuermann D. Mevissen M. (2021). Manmade electromagnetic fields and oxidative stress-biological effects and consequences for health. Int. J. Mol. Sci. 22, 3772. 10.3390/ijms22073772 33917298 PMC8038719

[B211] Schwartz L. Supuran C. T. Alfarouk K. O. (2017). The Warburg effect and the hallmarks of cancer. Anticancer Agents Med. Chem. 17, 164–170. 10.2174/1871520616666161031143301 27804847

[B212] Sepulchre R. Drion G. Franci A. (2019). Control across scales by positive and negative feedback. Annu. Rev. Control, Robotics, Aut. Syst. 2, 89–113. 10.1146/annurev-control-053018-023708

[B213] Sharma S. Wu S. Y. Jimenez H. Xing F. Zhu D. Liu Y. (2019). Ca2+ and CACNA1H mediate targeted suppression of breast cancer brain metastasis by AM RF EMF. EBioMedicine 44, 194–208. 10.1016/j.ebiom.2019.05.038 31129098 PMC6604768

[B214] Shen P. Larter R. (1995). Chaos in intracellular Ca2+ oscillations in a new model for non-excitable cells. Cell Calcium 17, 225–232. 10.1016/0143-4160(95)90037-3 7621534

[B215] Shinbrot T. (1995). Progress in the control of chaos. Adv. Phys. 44, 73–111. 10.1080/00018739500101506

[B216] Smedler E. Uhlén P. (2014). 'Frequency decoding of calcium oscillations. Biochim. Biophys. Acta 1840, 964–969. 10.1016/j.bbagen.2013.11.015 24269537

[B217] Sohn H. Cooper M. A. (2023). Metabolic regulation of NK cell function: implications for immunotherapy. Immunometabolism (Cobham) 5, e00020. 10.1097/IN9.0000000000000020 36710923 PMC9869966

[B218] Stangherlin A. (2023). Ion dynamics and the regulation of circadian cellular physiology. Am. J. Physiol. Cell Physiol. 324, C632–C643. 10.1152/ajpcell.00378.2022 36689675

[B219] Stupp R. Taillibert S. Kanner A. Read W. Steinberg D. Lhermitte B. (2017). Effect of tumor-treating fields plus maintenance temozolomide vs maintenance temozolomide alone on survival in patients with glioblastoma: a randomized clinical trial. JAMA 318, 2306–2316. 10.1001/jama.2017.18718 29260225 PMC5820703

[B220] Sun G. Li J. Zhou W. Hoyle R. G. Yue Z. (2022). Electromagnetic interactions in regulations of cell behaviors and morphogenesis. Front. Cell Dev. Biol. 10, 1014030. 10.3389/fcell.2022.1014030 36340041 PMC9627210

[B221] Sun H. Chen L. Cao S. Liang Y. Xu Y. (2019). Warburg effects in cancer and normal proliferating cells: two tales of the same name. Genomics, Proteomics and Bioinforma. 17, 273–286. 10.1016/j.gpb.2018.12.006 PMC6818181 31071451

[B222] Sun J. Tong Y. Jia Yu Jia Xu Wang H. Chen Y. (2023). Effects of extremely low frequency electromagnetic fields on the tumor cell inhibition and the possible mechanism. Sci. Rep. 13, 6989. 10.1038/s41598-023-34144-5 37117238 PMC10147919

[B223] Suzuki Y. Yamamura H. Imaizumi Y. Clark R. B. Giles W. R. (2020). K+ and Ca2+ channels regulate Ca2+ signaling in chondrocytes: an illustrated review. Cells 9, 1577. 10.3390/cells9071577 32610485 PMC7408816

[B224] Taghian T. Narmoneva D. A. Kogan A. B. (2015). Modulation of cell function by electric field: a high-resolution analysis. J. R. Soc. Interface 12, 20150153. 10.1098/rsif.2015.0153 25994294 PMC4590499

[B225] Tagne Nkounga I. B. Marwan N. Moukam Kakmeni F. M. Yamapi R. Kurths J. (2023). 'Adaptive resonance and control of chaos in a new memristive generalized FitzHugh-Nagumo bursting model. Chaos 33, 103106. 10.1063/5.0166691 37782829

[B226] Takada Y. Ye X. Simon S. (2007). The integrins. Genome Biol. 8, 215. 10.1186/gb-2007-8-5-215 17543136 PMC1929136

[B227] Taki M. Watanabe S. (2001). Biological and health effects of exposure to electromagnetic field from mobile communications systems. IATSS Res. 25, 40–50. 10.1016/s0386-1112(14)60069-8

[B228] Tanzhu G. Chen L. Xiao G. Shi W. Peng H. Chen D. (2022). The schemes, mechanisms and molecular pathway changes of Tumor Treating Fields (TTFields) alone or in combination with radiotherapy and chemotherapy. Cell Death Discov. 8, 416. 10.1038/s41420-022-01206-y 36220835 PMC9553876

[B229] Tiwari R. Kumar R. Malik S. Raj T. Kumar P. (2021). Analysis of heart rate variability and implication of different factors on heart rate variability. Curr. Cardiol. Rev. 17, e160721189770. 10.2174/1573403X16999201231203854 33390146 PMC8950456

[B230] Totland M. Z. Rasmussen N. L. Knudsen L. M. Leithe E. (2020). Regulation of gap junction intercellular communication by connexin ubiquitination: physiological and pathophysiological implications. Cell Mol. Life Sci. 77, 573–591. 10.1007/s00018-019-03285-0 31501970 PMC7040059

[B231] Tsong T. Y. (1988). Active cation pumping of Na+,K+-ATPase and sarcoplasmic reticulum Ca2+-ATPase induced by an electric field. Methods Enzymol. 157, 240–251. 10.1016/0076-6879(88)57080-5 2852752

[B232] Tuszynski J. A. (2019). “The bioelectric circuitry of the cell,” in Brain and human body modeling: computational human modeling at EMBC 2018. Editors Makarov S. Horner M. Noetscher G. (Cham (CH)). 31725207

[B233] Tuszynski J. A. Costa F. (2022). 'Low-energy amplitude-modulated radiofrequency electromagnetic fields as a systemic treatment for cancer: review and proposed mechanisms of action. Front. Med. Technol. 4, 869155. 10.3389/fmedt.2022.869155 36157082 PMC9498185

[B234] Tuszynski J. A. Friesen D. Freedman H. Sbitnev V. I. Kim H. Santelices I. (2020). Microtubules as sub-cellular memristors. Sci. Rep. 10, 2108. 10.1038/s41598-020-58820-y 32034179 PMC7005844

[B235] Tuszynski J. A. Wenger C. Friesen D. E. Preto J. (2016). An overview of sub-cellular mechanisms involved in the action of TTFields. Int. J. Environ. Res. Public Health 13, 1128. 10.3390/ijerph13111128 27845746 PMC5129338

[B236] Uthamacumaran A. (2021). A review of dynamical systems approaches for the detection of chaotic attractors in cancer networks. Patterns (N Y) 2, 100226. 10.1016/j.patter.2021.100226 33982021 PMC8085613

[B237] Uthamacumaran A. Zenil H. (2022). A review of mathematical and computational methods in cancer dynamics. Front. Oncol. 12, 850731. 10.3389/fonc.2022.850731 35957879 PMC9359441

[B238] Vaiciuleviciute R. Bironaite D. Uzieliene I. Ali M. Bernotiene E. (2021). Cardiovascular drugs and osteoarthritis: effects of targeting ion channels. Cells 10, 2572. 10.3390/cells10102572 34685552 PMC8534048

[B64] Van der Pol B. Van der Mark J. (1927). 'Frequency demultiplication. Nature 120, 363–364. 10.1038/120363a0

[B239] Vander Heiden M. G. Cantley L. C. Thompson C. B. (2009). Understanding the Warburg effect: the metabolic requirements of cell proliferation. Science 324, 1029–1033. 10.1126/science.1160809 19460998 PMC2849637

[B240] Vergun O. Votyakova T. V. Reynolds I. J. (2003). Spontaneous changes in mitochondrial membrane potential in single isolated brain mitochondria. Biophys. J. 85, 3358–3366. 10.1016/S0006-3495(03)74755-9 14581237 PMC1303613

[B241] Vianale G. Reale M. Amerio P. Stefanachi M. Di Luzio S. Muraro R. (2008). Extremely low frequency electromagnetic field enhances human keratinocyte cell growth and decreases proinflammatory chemokine production. Br. J. Dermatology 158, 1189–1196. 10.1111/j.1365-2133.2008.08540.x 18410412

[B262] von Stein A. Sarnthein J. (2000). Different frequencies for different scales of cortical integration: from local gamma to long range alpha/theta synchronization. Int. J. Psychophysiol. 38 (3), 301–313. 11102669 10.1016/s0167-8760(00)00172-0

[B242] Wallace J. Andrianome S. Ghosn R. Blanchard E. S. Telliez F. Selmaoui B. (2020). Heart rate variability in healthy young adults exposed to global system for mobile communication (GSM) 900-MHz radiofrequency signal from mobile phones. Environ. Res. 191, 110097. 10.1016/j.envres.2020.110097 32846174

[B243] Wang Y. Shi X. Si B. Cheng B. Chen J. (2023). Synchronization and oscillation behaviors of excitatory and inhibitory populations with spike-timing-dependent plasticity. Cogn. Neurodyn 17, 715–727. 10.1007/s11571-022-09840-z 37265649 PMC10229527

[B244] Warburg O. (1956). On the origin of cancer cells. Science 123, 309–314. 10.1126/science.123.3191.309 13298683

[B245] Watson S. A. McStay G. P. (2020). Functions of cytochrome c oxidase assembly factors. Int. J. Mol. Sci. 21, 7254. 10.3390/ijms21197254 33008142 PMC7582755

[B246] West G. B. Brown J. H. Enquist B. J. (1997). A general model for the origin of allometric scaling laws in biology. Science 276, 122–126. 10.1126/science.276.5309.122 9082983

[B247] Wheless J. W. Gienapp A. J. Ryvlin P. (2018). Vagus nerve stimulation (VNS) therapy update. Epilepsy Behav. 88s, 2–10. 10.1016/j.yebeh.2018.06.032 30017839

[B248] White K. A. Grillo-Hill B. K. Barber D. L. (2017). 'Cancer cell behaviors mediated by dysregulated pH dynamics at a glance. J. Cell Sci. 130, 663–669. 10.1242/jcs.195297 28202602 PMC5339414

[B249] Wilson D. F. Matschinsky F. M. (2021). 'Metabolic homeostasis in life as we know it: its origin and thermodynamic basis. Front. Physiol. 12, 658997. 10.3389/fphys.2021.658997 33967829 PMC8104125

[B250] Wonderlin W. F. Strobl J. S. (1996). 'Potassium channels, proliferation and G1 progression. J. Membr. Biol. 154, 91–107. 10.1007/s002329900135 8929284

[B251] Wust P. Kortum B. Strauss U. Nadobny J. Zschaeck S. Beck M. (2020). Non-thermal effects of radiofrequency electromagnetic fields. Sci. Rep. 10, 13488. 10.1038/s41598-020-69561-3 32778682 PMC7417565

[B252] Wust P. Stein U. Ghadjar P. (2021). Non-thermal membrane effects of electromagnetic fields and therapeutic applications in oncology. Int. J. Hyperth. 38, 715–731. 10.1080/02656736.2021.1914354 33910472

[B253] Wust P. Veltsista P. D. Oberacker E. Yavvari P. Walther W. Bengtsson O. (2022). Radiofrequency electromagnetic fields cause non-temperature-induced physical and biological effects in cancer cells. Cancers (Basel) 14, 5349. 10.3390/cancers14215349 36358768 PMC9655505

[B254] Xing F. Luan Y. Cai J. Wu S. Mai J. Gu J. (2017). The anti-warburg effect elicited by the cAMP-pgc1α pathway drives differentiation of glioblastoma cells into astrocytes. Cell Rep. 18, 468–481. 10.1016/j.celrep.2016.12.037 28076790 PMC5926788

[B255] Yang M. Brackenbury W. J. (2013). 'Membrane potential and cancer progression. Front. Physiol. 4, 185. 10.3389/fphys.2013.00185 23882223 PMC3713347

[B256] Young A. Hunt T. Ericson M. (2021). The slowest shared resonance: a review of electromagnetic field oscillations between central and peripheral nervous systems. Front. Hum. Neurosci. 15, 796455. 10.3389/fnhum.2021.796455 35250508 PMC8888685

[B257] Zeng S. Hu X. (2023). Lactic acidosis switches cancer cells from dependence on glycolysis to OXPHOS and renders them highly sensitive to OXPHOS inhibitors. Biochem. Biophysical Res. Commun. 671, 46–57. 10.1016/j.bbrc.2023.05.097 37295355

[B258] Zhou Y. Wong C. O. Cho K. J. van der Hoeven D. Liang H. Thakur D. P. (2015). Signal transduction. Membrane potential modulates plasma membrane phospholipid dynamics and K-Ras signaling. Science 349, 873–876. 10.1126/science.aaa5619 26293964 PMC4687752

[B259] Zhu K. Takada Y. Nakajima K. Sun Y. Jiang J. Zhang Y. (2019). Expression of integrins to control migration direction of electrotaxis. FASEB J. 33, 9131–9141. 10.1096/fj.201802657R 31116572 PMC6662972

[B260] Zimmerman J. W. Jimenez H. Pennison M. J. Brezovich I. Morgan D. Mudry A. (2013). Targeted treatment of cancer with radiofrequency electromagnetic fields amplitude-modulated at tumor-specific frequencies. Chin. J. Cancer 32, 573–581. 10.5732/cjc.013.10177 24206915 PMC3845545

[B261] Zimmerman J. W. Pennison M. J. Brezovich I. Yi N. Yang C. T. Ramaker R. (2012). Cancer cell proliferation is inhibited by specific modulation frequencies. Br. J. Cancer 106, 307–313. 10.1038/bjc.2011.523 22134506 PMC3261663

