# Supplementary material for: Emerging cancer therapies: targeting physiological networks and cellular bioelectrical differences with non-thermal systemic electromagnetic fields in the human body – a comprehensive review
Source: Front Netw Physiol. 2024 Dec 10;4:1483401. doi: 10.3389/fnetp.2024.1483401 (PMC11666389; doi:10.3389/fnetp.2024.1483401)
Supplement: Supplementary file 1 [file DataSheet1.PDF]

Supplemental Information

We included only experimental studies conducted in vitro and in vivo that examine the relationship between controlled exposures to extremely low frequency radiofrequency EMF and low energy (up to 10 mT) in normal and cancerous mammalian cells. Only peer-reviewed articles reporting results from primary studies will be considered eligible. We searched the scientific literature databases NCBI PubMed and EMF-Portal for articles from the last 15 years as conducted by Schuermann et al (Schuermann and Mevissen 2021). There will be no restrictions on publication dates. Studies must be published in English to be included. The reference lists of the experimental studies were compiled in the table below to compare common key characteristics.

Results were presented using the folowing symbols:
↑ - associated measure was significantly increased ($p<0.05$) in exposed animals/cells
↓ - associated measure was significantly decreased ($p<0.05$) in exposed animals/cells
◆ - associated measure was not significantly changed ($p\geq0.05$) in exposed animals/cells

Table included the folowing annotations:
3-NT – 3- nitrotyrosine
8-OHdG – 8-hydroxydeoxyguanosine
AOPP – advanced oxidative protein product
CAT – catalase
FapyAde – 4,6-diamino-5-formamidopyrimidine
FapyGua – 2,6-diamino-4-hydroxy-5-formamidopyrimidine

GPx – glutathione peroxidase

GSH – GSH level, GSH/GSSG

GSHre – GST reductase, GR activity

GST – glutathione S transferase

LP – lipid peroxidation

MDA – malondialdehyde

NO – nitrogen oxide

NOS – nitric oxide synthetase

ORAC – oxygen radical absorbance capacity

OSI – oxidative stress index

oxDNA – oxidative DNA damage

PCG – protein carbonyl groups

ROS – reactive oxygen species

SOD – superoxide dismutase

T-AOC – total antioxidant capacity

TAL – total antioxidant level

TOL – total oxidant level

TT – tissue toxicity as defined by pathological changes

In-vitro experiments

Authors	Study type	Objective	Cell Line	Source	Organ/tissue	Freq. (Hz)	Time	Dose (mT)	Antioxidant/ co-treatment	Metodology	Exposure system	Oxidative stress markers	Other results
(Akan, Aksu et al. 2010)	*in vitro*	immune response	THP-1	Human	leukemic monocytes	50	1-6 hr	1	*Staphylococcus aureus,* IFg/LPS	*nitric oxide analyzer (NOA280i, Sievers Instruments, Boulder, CO) was used for the determination of NO metabolite nitrite (NOx) in the samples; cGMP ELISA test; Caspase-9 FLICA kit FAM-LEHD-FMK*	*ELF-EMF was generated by a custom-made Helmholtz coil system having a 25 cm diameter*	↑NO, ◆iNOS	↑HSP, ↑cGMP, ↓Caspase-9 activation, ↑HSP70 altered pathogene response
(Ayşe, Zafer et al. 2010)	*in vitro*	time schedule	K562	Human	leukemic myelocytes	50	1 hr or 1 hr daily for 4 days	5	None	p-nitro blue tetrazolium (NBT, Molecular Probes)	Eight solenoid bobbins made of copper, which had 860 turns, and an iron core were serially connected	↑ROS	immediate after and but no increase after 2 h of exposure (transient effect)

(Benassi, Filomeni et al. 2016)	*in vitro*	Redox Homeostasis	SH-SY5Y	Human	brain/neuroblastoma	50	1-3 days	1	N-acetyl-L-cysteine, MPTP	stained with DHE and H2−DCFDA in order to detect superoxide; CellEvent™ Caspase-3/7 Green Detection kit for caspase; Protein Carbonylation Carbonylated proteins detection using the Oxyblot Kit	two couples of square coils (two coils for each system) designed in Helmholtz configuration	↑ROS, ↑PCG	◆apoptosis, ◆cell proliferation ↑MPTP toxicity, altered redox homeostasis
(Bergandi, Grisolia et al. 2019)	*in vitro*		MSTO-211H MDA-MB-231 MCF-10A Beas-2B	Human	breast cancer mesothelioma breast epithelial vell bronchial epithelial cell			0.1	none		two independent couples of coaxial coils made of 200 loops. The outer coils were supplied with a direct current (DC) that provided a constant magnetic field of 45 µT		

(Brisdelli, Bennato et al. 2014)	*in vitro*	apoptosis induction protection	K562	Human	leukemic myelocytes	50	1-3 days	1	quercetin	double acridine orange and ethidium bromide staining for apoptosis; Annexin-V-FITC assay, Ac-DEVD-AMC for caspase activity, DCF fluorescence for ROS, Cell cycle analysis by flow cytometry	Helmholtz coils system	◆ROS	◆ apoptosis, ◆Cell proliferation decreased querceptin toxicity, protective effect of ELF-MF on quercetin-induced apoptosis, ↓ G2/M treated with quercetin, ◆Bcl-2, Bcl-xL, and Mcl-1
(Bułdak, Polaniak et al. 2012)	*in-vitro*	cisplatin synergy	AT478	Mouse	squamous cell carcinoma	50	16 min (assessed 24, 48, 72 hr) 6, 24, 72 hr (ROS)	1	Cisplatin	(H2DCF-DA)-loaded cells (Molecular Probes, Leiden, Netherlands) using a fluorescent measurement system, SOD concentration by GSH-Px assay, protein concentration was measured using a Bio-Rad protein reagent, MDA was determined by the thiobarbituric acid (TBA) reaction	20 cm diameter 40 cm length solenoid derived from a medical device (MRS-2000, Vita-Life, Balzers, Liechtenstein)	↑ROS, ↑SOD, ↓MDA, ↑GPx	↑DNA damage (synergy w cisplatin)

(Buckner, Buckner et al. 2015)	*in-vitro and in-vivo male C57b mice*	Cell viability, calcium influx	B16-BL6 HSG MDA-MB-231 MCF-7 HeLa HBL-100 HEK293	Human Mouse	mouse melanoma salivary gland breast cancer breast epithelial kidney cell	Thomas frequencies (6-25Hz)	1h/day, 4h/day (cells) 3h/day (mice)	2–10 (μT)	none	Cell viability, DNA fragmentation assay, calcium influx by fluorescence, flow cytometry	12 cm on each side and fitted with 6 solenoids, one on each face of the cube. Plates are placed inside the cube for exposure. Invivo exposure device is 30 cm on each side andfitted with 6 solenoids, one on each face of the cube.		↓cell proliferation in cancer, ◆cell proliferation in normal cells, ◆apoptosis in cells ↓cell proliferation in cancer in mice ↑Calcium influx in cancer but not in normal cells
(Calcabrini, Mancini et al. 2017)	*in vitro*	ROS production	NCTC-2544	Human	normal keratinocytes	50	1-4 hr	0.025, 0.05, 0.1, 0.15, 0.2	None	ROS production using the oxidation-sensitive probe dihydrorhodamine (DHR), Trypan Blue Exclusion Assay, disappearance of NADH during LDH-catalysed conversion of pyruvate to lactate, SOD activity	three parallel copper plates (40x40 cm), with the center plate carrying a total current of I, and the outer two carrying a current of -I/2 in the opposite direction	↑ROS (0.05/0.1 mT, 1/2 hr) fluctuating OS Markers	effects vary w density and duration of cell exposure, increased (↑ROS after 30 min)

(Chen, Hong et al. 2014)	*in-vitro*	authophagy apoptosis	MEF	Mouse	normal embryonic fibroblasts	50 (MF)	6 hr (assessed 0.5, 2, 6, 12, 24 hr post exposure)	2	Rapamycin	autophagosomes using TEM, Intracellular ROS levels were measured using a ROS Assay Kit, flow cytometry for apoptosis, TUNEL assays	sXc-ELF, IT'IS Corporation	↑ROS (2, 6 hr post exposure)	◆ apoptosis, ↑autophagy
(Chen, Hong et al. 2014)	*in-vitro*	Oxative stress, Cell viability and apoptosis	HEK293 786-O 769-P Caki1	Human	clear cell renal carcinoma	50	1 h	4.5	None	ROS determination by CellROx green reagent probes, Cell viability assay, migration and invasion assays, cell cycle analysis, apoptosis	Helmholtz coil that generated a field and the magnetic induction	↑ROS	◆TT, ↑apoptosis, ↓cell viability, ↑ G1 phase cell arrest, ↓migration, ↓invasion

(Consales, Cirotti et al. 2018)	*in-vitro*	gene expression mediated by miRNAs	SH-SY5Y	Human	brain/neuroblastoma (human) primary cortical neurons (mouse)	50	up to 72 hr	1	N-acetyl-L-cysteine	RNA was evaluated by fiber optic spectrophotometer, pri-miR-34b/c p53, Btg4, and Snca expression, RNA extracted with the miRcury RNA isolation kit, FACScan Flow cytometer, DNA methylation analysis by PCR, Fluorescence Microscopy for Quantification of α-Synuclein Aggregates	two coils for each sub-system, arranged coaxially in Helmholtz configuration	↑ROS	↑miR-34b/c, ↓ pri-miR-34b/c, ◆p53, ↑DNA methylation
(Consales, Panatta et al. 2019)	*in-vitro*	gene expression (iron-regulated genes	SOD1 G93A (mut) SH-SY5Y	Human	brain/neuroblastoma	50	up to 72 hr	1	None	FACScan Flow cytometer for cell cycle distribution, oxidative stress by DHE or H2-DCF-DA, Total RNA by Trizol, cDNA using SYBR Green master mix,	two coils for each sub-system, arranged coaxially in Helmholtz configuration	◆ROS	deregulation IRP1, MFRN1 and TfR1

(Costantini, Sinjari et al. 2019)	*in-vitro*	modulation of inflammatory cytokines, cell metabolism, gene expression	primary	Human	normal gingival fibroblast	50 (pulsed 12 Hz)	6, 18 hr	1	None	two EMF signals: sinusoidal electromagnetic field (SEMF), and a pulsed electromagnetic field (PEMF), MTT assay for cell metabolism	device composed of a 160-turn solenoid (22 cm length, 6 cm radius, 1.25 10□5 cm copper wire diameter)	↑iNOS (6 hr) ↑HO-1 (18 hr)	↑Cell proliferation (SEMF), ↑cell metabolic activity, ↑ IL-6 and TGF-B, ↓MMP-2
(de Groot, Kock et al. 2014)	*in-vitro*	membrane integrity, oxidative stress, calcium-pathway	PC12	Rat	pheochromocytoma	50	up to 48 hr	0.001, 0.01, 0.1, 1	Dexamethasone, L-Dopamin, $FeSO_4$	Ca2+-sensitive fluorescent ratio dye Fura-2 AM, CFDA-AM assay for membrane integrity, ROS by fluorescent dye H2-DCFDA	two custom-made devices (Immunent BV, Velthoven)	◆ROS	◆ Calcium influx ◆membrane alterations
(Destefanis, Viano et al. 2015)	*in-vitro*	Mintochondrial activity	SKBR3 GTL16 HT29 A375P	Human	breast cancer cell line, gastric cancer cell line, human colon cancer cell line and melanoma	50	1 h	4.5	None	Mitochondrial membrane potential, ATP levels, LDH determination by Western blot, flow cytometry and RNA by RT-PCR	two coaxial coils made of 200 loops (formed of copper wire, 0.3 mm in diameter) each loop being 2.5 cm in length and wound into plastic frame. The frame had a cylindrical shape with an outer radius of 8 cm, and the distance between the two coaxial coil couples was 8 cm		↓cell proliferation, mitochondrial membrane potential variation, ◆cytochrome c oxidase subunit II and subunit IV, ◆ proein leves for phospho-ERK, p53, and cytochrome c

(Duan, Liu et al. 2015)	*in-vitro*	cell viability	GC-2	Mouse	spermatogenic cells	50 (or 1800 MHz)	24 hr	1, 2, 3	None	Cell Counting Kit-8 for viability, alkaline comet assay, FPG-sensitive sites, Immunofluorescence detection of c-H2AX,	sXc-ELF, IT'IS Corporation	↑oxDNA (3 mT)	◆cell viability, ↑DNA damage, ↑nuclear c-H2AX Foci Formation
(Duong and Kim 2016)	*in-vitro*	oxidative stress and calcium levels	HMO6	Human	normal microglia	50 (10/100)	4 hr	0.01, 0.1, 1	Oxygen and glucose deprivation	DCFH-DA fluorescent for ROS and calcium	two identical coils to generate the uniform vertical magnetic fields with following parameters: axial symmetry, 2D; diameter of two coils, inner 15 cm, outer 26 cm; distance between coils, 18 cm; coil diameter, 18 AWG; number of loops, 1000	↓ROS	↓ intracellular calcium
(Falone, Mirabilio et al. 2008)	*in-vitro and in-vivo male Sprague–Dawley rats*	Oxidative stress and aging	neurons	Rat	Brain cortices were homogenized	50	5, 10 days	1 0.1	None	SOD, glutamine redutase, catalase and gene expression determination	a pair of Helmholtz coils (r = 630 mm, distance between coils = 700 mm) (Oersted Technology Corp., Oregon, USA).	↓GST, ↑SOD2	↓DNA, ↑NRF2, ↑ redox protection
(Feng, Dai et al. 2016)	*in-vitro*	oxidative stress and EGFR clustering	FL	Human	amniotic epithelial cells	50	5, 15, 30, 60 min	0.1, 0.2, 0.4	None	clustering of EGFR with confocal laser scanning microscope	sXc-ELF, IT'IS Corporation	↑ROS (5-30 min, 0.2/0.4 mT)	↑ EGFR clustering

(Feng, Qiu et al. 2016)	*in-vitro*	mitochondrial permiability and ROS levels	FL	Human	amniotic epithelial cells	50	5, 15, 30, 60 min	0,4	N-acetyl-L-cysteine	MPTP Fluorescent Assay Kit for mitochondrial permeability, mitochondrial membrane potential assay kit,	sXc-ELF, IT'IS Corporation	↑ROS (5-30 min)	↑mitochondrial permibility (60 min), ↑phosphorylation of GSK-3b, ◆Bax and Bcl-2
(Feng, Ye et al. 2016)	*in-vitro*	mitochondrial ROS levels and cell viability	FL	Human	amniotic epithelial cells	50	30, 60, 120 min	0,4	None	Apoptosis Detection Kit, CCK-8 kit for cell viability, MitoSOX Red kit for mitochondrial ROS	sXc-ELF, IT'IS Corporation	↑ROS (mitochondrial)	↓apopotosis, ↑MAPK signalling
(Frahm, Mattsson et al. 2010)	*in-vitro*	Oxidative stress and signal transduction	primary	Mouse	macrophages	50	5, 15, 30, 45 min	1	TPA, LPS	DHR dye for ROS and flow cytometry	horizontally polarized sine-wave 50 Hz magnetic field with a flux density of 1.0 mT	↑ROS (45 min), ↑NOX	↑NAD(P)H oxidase complex gp91phox, ◆Hsp70 and Hsp110, ◆PI3-kinase, PKB and PP2A
(Hong, Han et al. 2012)	*in-vitro*	Oxidative stress	MCF10A	Human	nomal mammary epithelial cells	60	4 h (assessed 10-48 h after)	1	None	kit-WST for SOD, GSH/GSSG-412 kit for reduced and oxidized, fluorecence for ROS, glutathione	equipment was designed and constructed by Korea Electrotechnology Research Institute	◆ROS, ◆GSH, ◆SOD	◆SOD, ◆GSH/GSSG ratio,◆cellular senescence
(Höytö, Herrala et al. 2017)	*in-vitro*	*mitochondrial ROS levels and DNA damage*	SH-SY5Y	Human	brain/neuroblastoma	50	24 hr	0,1	Menadione, blue light	alamarBlue for cell proliferation, MitoSOX Red for mitochondrial superoxide indicator,Cytoplasmic ROS with DHE, SYTOX green for micronuclei formation	a horizontal MF. Wavetek Waveform Generator model 75 (Wavetek, San Diego, CA, USA) and Peavey M-3000 Power Amplifier	↑ROS (cytosolic) ↓ROS (mitochondrial)	◆cell viability, ◆micronuclei formation

(Huang, Chang et al. 2014)	*in-vitro*	Cell viability and Cell cycle and trasductive pathway	HaCat	Human	keratinocytes	60	96 h, 72 h, 48 h, 12 h, 8 h, and 4 h	1.5	None	RNA isolation with an RNeasy Mini Kit. cDNA purification using Microcon YM-30 column. Realtime PCR, cell viability, cell cycle analysis, western blot and siRNA transfection	Helmholtz coil system consisting of 100 turns of copper wire in each coil, and situated inside a tissue culture incubator. The pair of coil apparatuses each had a diameter of 34 cm and were 17 cm apart		↑CDKN1A, ↓ CDC25B, CDC20, CDC2, CCNA2 and CCNB1, ↓Cell proliferation, G1-phase arrest, ↑ p21, ↑ATM-Chk2-p21 pathway
(Jeong, Kim et al. 2017)	*in-vitro*	oxidative stress and astrocyte differerentation	BM-MSC	Human	bone marrow stem cells	50	12 days	1	None	CCK-8 for proliferation analysis, DCFDA for ROS, RT-PCR forGFAP, Nestin, SIRTI,OCT3/4,GAPDH	a pair of Helmholtz coils NOS	↑ROS	↓proliferation rate, ↑ astrocyte differentiation,↑SIRTI
(Jimenez, Wang et al. 2019)	*in-vitro and In-vivo SCID mice*	Cell viability, cell cycle and CCD2 activation	Huh7 HepG2 Hep3B HCCLM3 & others	Human Mouse	Liver	27MHz (carrier) amplitude modulated envelope 500 Hz to 22Khz	3 h/day	30 and 400 mW/kg	None	Cell viability assay by Thymidine incorporation, flow cytometry, western blot, RT-PCR for RNA a cDNA, gene set enrichment analysis, knockdown and overexpression . Intracranial injection for *in-vivo* experiments	sXc-ELF and sXv27, IT'IS Corporation		↓ cell proliferation, ↓Ki67% and cyclin D1, ↑p21, ↑calcium influx, ↑Voltage-gated calcium channels

(Kesari, Juutilainen et al. 2016)	*in-vitro*	oxidative stress and cell viability	SH-SY5Y	Human Rat	brain/neuroblastoma	50	24 hr	0.01, 0.03	Menadione	flow cytometric micronucleus analysis, Mitochondrial and cytosolic superoxide productions by MitoSOX Red, Fluorometric detection using DHE, cell viability by fluorecence	Horizontal MF was generated by a pair of coils (340 x 460 mm) in a Helmholtz-type configuration (220 mm distance between the coils)	↑ROS (co-treatment) ◆ROS (ELF-MF alone)	◆cell viability, ◆micronuclei formation
(Kesari, Luukkonen et al. 2015)	*in-vitro*	Oxidative stress and DNA damage	SH-SY5Y	Human	brain/neuroblastoma	50	24 hr (assessed 15, 30, 45 days later)	0,1	N-acetyl-L-cysteine, menadione	Lipid peroxidation by DPPP, ROS production by DCFH-DA and relative cell number	Horizontal MF was generated by a pair of coils (340 x 460 mm) in a Helmholtz-type configuration (220 mm distance between the coils)	↑ROS (45 days), ↓LP	↑DNA micronuclei formation
(Kim, Jang et al. 2017)	*in-vitro*	Activation	RAW 264.7	Mouse	macrophage	60	10, 20 hr	0,8	LPS	CFSE for proliferation analysis, FITC for fagocitic analysis, NO assay, calcium-indicator dye fluo-4 acetoxymethyl, ROS assay	Electrotechnology Research Institute (Dankook University, Cheonan, Republic of Korea	↑NO ◆NO (ELF-MF alone)	↑TNF-a, IL-6, and IL-1b, ↑ p-IkB-a, NF-kB-Dependent and NFAT-Dependent Signaling Pathways,◆cell proliferation, ◆phagocytosis

(Lazzarini , Eléxpuru-Zabaleta et al. 2023)	*in-vitro*	oxidative stress, cell morphology, apoptosis, cell viability and gen expression	MDA-MB-231 MCF-10A	Human	breast cancer and normal breast epithelial cell	50	4 h	1		Cell viability by Trypan blue assay, apoptosis Apoptosis detection kit, phalloidin staining, electron microscopy, and ROS by MitoSOX red, Mass Spectrometry analysis, Functional enrichment analyses	Self-designed, the coil was designed with dimensions (inner coil sides, 34 ×34 cm) and the distances between the two solenoids (19.5 cm)	↑ ROS cancer and normal cell	↑ cell viability in cancer, ↑ TT in cell and mitochondrial morphology, ↑ difference in expressed genes in cancer, ↑KLF4, STAT3, BCLAF1 and NR2C2 in cancer
(Lekovic, Drekovic et al. 2020)	*in-vitro*	Oxidative stress	MRC-5	Human	normal lung fibroblast	50	1/2/3/7 days	10	None	cell proliferation by MTT assay, NBT assay for NO and ROS, GSH GSSG by by 5.5-dithio-bis-6.2-nitrobenzoic acid, SOD by autoxidation of pyrogallol, catalase by Beutler method	electromagnetic therapy device EkoMedico-Magnet (Electronic Design, Belgrade, Serbia)	↓NO (day 1-3), ↑NO (day7) ↓↑◆ROS/GSH /CAT, ◆SOD, ↑GST/GSHRe/ GPx	
(Li and Heroux 2014)	*in-vitro*	Cromossome count and cell viability	K562 HEL92.1.7 MCF7 NCIH460 COLO320 DM	Human	erythroleukemia, breast cancer, lung cancer and colon cancer	60	6 days - 3 weeks	0.025–5 (μT)	Metformin Resistin Metformin	ROS determination by CellROx green reagent probes, Cell viability assay, migration and invasion assays, cell cycle analysis, apoptosis	rectangular coils (19 25.6 cm) with 20–50 turns of #25 AWG varnished copper wire wound on 13mm polycarbonate		↓cell proliferation, shift in average chromosome numbers

(Liu, Liu et al. 2016)	*in-vitro*	Cell viability, cell cycle and CCD2 activation	GC-2	Mouse	spermatocyte -derived	50	72 h (5 min on/10 min off)	3	None	RNA straction by Trizol reagent kit for RT-PCR. cDNA obtained by the GoScriptTM Reverse Transcription System Kit, cell viability assay by CCK-8, flow cytometry, western blot, miRNA transfection	sXc-ELF, IT'IS Corporation	↑ROS (older neurons) ◆ROS (younger neurons) ↑Nox2 (8 hr/day)	↑ miR-26b-5p, ↓CCND2
(Luukkonen, Liimatainen et al. 2014)	*in-vitro*	Oxidative stress	SH-SY5Y	Human	brain/neuroblastoma	50	24 hr (assessed 3 hr after exposure or later)	0,1	Menadione	micronucleus analysisby EMA, Mitochondrial and cytosolic superoxide production, ROS production, and reduced glutathione (GSH), DPPP for lipid peroxidation and mitocondrial activity by MPP	Induction of genomic instability, oxidative processes, and mitochondrial activity by 50Hz magnetic fields in human SH-SY5Y neuroblastoma cells	↑ROS (mitochondrial), ↓LP	↑micronuclei formation

(Maiullari, Cicirelli et al. 2023)	*in-vitro*	metabolism, apoptosis, cell viability and stress response	Cat.T0034	Human	skeletal muscle cells	75	4 h	1.5		Cell viability assay by MTT,apoptosis by Annexin V-FITC/7-AAD Kit, stress-related protein by proteom profile	Two copper wire Helmholtz coils placed on opposing sides of a plexiglass container were used to build the generators. Then, 6-well plates or 25 cm flasks containing cells were placed in the plexiglass container in the cell incubator		◆metalobism, ◆apoptosis, ↑cell proliferation,↑MyoD expression,↑ cell repair, ↑ HSP70, HIF-2a, Cytochrome c, PON3 and p21/CIP1, ↓ Phospho-JNK Pan, PON2, SIRT2, SOD2 and thioredoxin-1
(Mousavi Maleki, Entezari et al. 2022)	*in-vitro*	Cell viability and gene expression	AGS-C10071	Human	gastric cancer cell	50-60	18 h, 1.5 h 12h intervals	0.2-2	None	Cell viability assay by MTT, RNA and cDNA analysis by PCR	a solenoid cylinder with a diameter of 12 cm, a height of 30 cm, and 1200 revolutions of copper wire with a diameter of 1 mm in 4 rows		↓cell viability, ↑CTSL2 and SOCS3,
(Mannerling, Simkó et al. 2010)	*in-vitro*	Oxidative stress	K562	Human	leukemic myelocytes	50	1 hr	0.025, 0.05, 0.1	melatonin, 1,10-phenantroline	a flow cytometer for cell proliferation and cell cycle analysis, Western blot analysis for HSP70, NBT assay superoxide radical anion analysis	double Helmholtz coil arrangement (made in-house), with two horizontal and two vertical coils (ø 40 and 42 cm, respectively)	↑ROS	↑HSP70, ◆cell proliferation and cell cycle distribution,

(Martínez , Úbeda et al. 2021)	*in-vitro*	Oxidative stress and cell proliferation pathways	NB69	Human	brain/neuroblastoma	50	24, 42, 62 hr 15-120 min	0,1	N-acetyl-L-cysteine	DCFH-DA for free-radical detection, MAPK-p38, -ERK1 /2 and -JNK by Western blotting and immunofluorescence analysis	a pair of coils set in Helmholtz configuration	↑ROS	↑Cellular proliferation, ↑p67phox expression, ↑free radicals, ↑p-ERK1/2 and JNK overexpression
(Morabito , Guarnieri et al. 2010)	*in-vitro*	neuritogenesis	PC12	Rat	pheochromocytoma	50	30 min or 7 days	0.1, 1	None	MTT for cell growth and viability, H2 DCF-DA, Fluo-4 AM or DIBAC4 for ROS and fluorescences for Calcium signalling	pair of Helmholtz coils (r= 445 mm, distance between coils 400 mm) (Oersted Technology Corp., Troutdale, OR, USA)	↑ROS (1 mT), ↓Catalase ◆ROS (differenting)	◆cellular proliferation, ◆intracellular calcium
(Nakayama, Nakamura et al. 2016)	*in-vitro*	Oxidative stress and DNA damage	RAW 264.7	Mouse	macrophage	50	24 hr	0,5	LPS	alkaline comet assay for DNA single-strand breaks (SSB), trypan blue exclusion method for cell viability, and fluorescent indicator diaminonaphthalene for nitric oxide (NO) production	four square coils constituted one Merritt coil and was constructed with two electric wires wound in parallel around an aluminum frame (Hozen Industries, Kyoto, Kyoto, Japan)	◆NO	↑DNA SSB in LPs stimulated, ↓cellular viability in LPS stimulated

(Nezamtaheri, Goliaei et al. 2022)	*In-vivo*	Oxidative stress , cell viablity, cell cycle arrest	DU145 HUVEC K562 MDA-MB-231	Human	Leukemia cell Breast cancer Normal cells	0.01, 0.1, 1, 10	120 h	100	None	fluorescence labeling and detection of apoptotic and necrotic cells, cell viability, autophagy, cell cycle, ROS production	field produced by by the iron core with max field created in the air gap (2 cm) and the size of the core (square with a 10 cm side). 2 coils connected in series (with wires of 1.5 mm diameter), with a total number of 1000 turns	↑ROS, ↑autophagy	↑apoptosis in adherent cells (cancer and normal), ↓cell proliferation, ↑G2/M phase
(Park, Seo et al. 2013)	*in-vitro*	Oxidative stress and cell differentiation	BM-MSC	Human	bone marrow stem cells	50/100	30-180 min or up to 8 days	1	N-acetyl-L-cysteine	MTT for cell growth and viability, H2DCF-DA for ROS	two Helmholtz coils 109 (15 cm inner diameter) oriented to produce a vertical magnetic 110 field	↑ROS (90 min)	ROS-mediated differentiation, ↑EGF/Akt signaling
(Patruno, Amerio et al. 2010)	*in-vitro*	Oxidative stress and cell proliferation	HaCaT	Human	normal keratinocytes	50	3, 18, 48 hr	1	LPS	spectrophotometrically for ROS and Catalase, PGE2 levels by PGE2 EIA kit; Greiss reagent for NO	a 160-turn solenoid (22 cm length, 6 cm radius, copper wire diameter of 1Æ25 · 10)5 cm) generating a horizontal magnetic field	↑NO, ↑iNOS (1 hr), ↓ROS	↑Cellular proliferation

(Patruno, Costantini et al. 2020)	*in-vitro*	Oxidative stress and signal transduction	THP-1	Human	leukemic monocytes	50	1/6/24 hr	1	LPS	Zymography for matrix metalloprotein ase, liquid scintillation spectrometry for NOS, spectrophoto metrically for Catalase and SOD, TIMP-1 byTIMP-1 Immunopreci pitation assays	160-turn solenoid coil (22 cm length, 6 cm radius, copper wire diameter of 1.25 × 10−5 cm) connected to a power amplifier (NAD electronics Ltd., model 216, London, UK)	↑↓ROS, ↑↓HO-1 ↑↓GSH/GPx/G SHRe	↑Akt/Erk/NRF2 signaling
(Patruno, Pesce et al. 2012)	*in-vitro*	Oxidative stress and matrix metalloprotei nases	THP-1	Human	leukemic monocytes	50	24 hr	1	LPS	western blot for:HO-1, lamin B, SIRT-1, p-p65), p65, Akt, p-Akt , p-ERK, ERK, and β-actin, H2DCF-DA for ROS, Glutathione Assay by fluorecence, spectrophoto metrically for GPX and GR activity	160-turn solenoid coil (22 cm length, 6 cm radius, copper wire diameter of 1.25 × 10−5 cm) connected to a power amplifier (NAD electronics Ltd., model 216, London, UK)	↑iNOS, ↑ROS, ↓SOD/CAT, ↑3-NT	↑TIMP-1
(Patruno, Tabrez et al. 2015)	in-vitro	Oxidative stress	K562	Human	erythro-leukemic cell	50	1-24 hr	1	PMA	spectrophoto metrically for Catalase and SOD, iNOS by conversion of L-(2,3-3H) arginine to L-(2,3-3H) citrulline in cell homogenates	160-turn solenoid coil (22 cm length, 6 cm radius, copper wire diameter of 1.25 × 10−5 cm) connected to a power amplifier (NAD electronics Ltd., model 216, London, UK)	↑CAT (1, 3 hr), ↑iNOS	↑CYP-450 modulation of PMA effect

(Errico Provenzano, Amatori et al. 2018)	*in-vitro*	Oxidative stress and cell differentiation	NB4	Human	acute promyelocytic leukemia	50	up to 96 hr	2	All-trans retinolic acid (ATRA) N-acetyl-L-cysteine	CD11b and CD14 by FACS analysis, ROS by DCFDA, Western blot analysis for MAPK, NFkB p65 and anti-αTubulin	Electromagnetic Fields Laboratory of the Department of Pure and Applied Sciences (University of Urbino)	↑ROS (with ATRA)	◆cell proliferation, ↑ ATRA differentiation with involvement of ROS and ERK pathways
(Ramazi, Salimian et al. 2023)	*in-vitro*	Oxidative stress, cell viability and cell cycle	MCF-7	Human	foreskin fibroblast	50	12, 24, and 48 h	0.5-90	Doxorubicin	Cell viability assay by MTT,apoptosis by AO/PI fluorescence probe, RNA and cDNA analysis by PCR, DCFH-DA kit for ROS, cell cytometry	a locally designed with two coils with direct current (DC) , the coils were made of 180 turns of copper wire. With the current via two parallel horizontal iron blades with 1 m heights and 10 cm2 surface area	↑ROS	↓cell viability, ↑TT, ↑cell arrest at phase G0-G1 and G2/M
(Reale, Kamal et al. 2014)	*in-vitro*	Oxidative stress	SH-SY5Y	Human	brain/neuroblastoma	50	1, 3, 6, 24 hr	1	None	NOS by conversion of L-(2,3-3H)arginine to L-(2,3-3H)citrulline, ROS and CAT by spectrophotometrically, cell viability by MTT	160 turn solenoid (22 cm length, 6 cm radius, copper wire diameter of 1.2561025 cm) that generated a horizontal magnetic field.	↑ROS, ↓CAT (6 hr) ↑nNOS	↑TGFb and IL-18BP, ↑CYP 450 activity

(Sharma, Wu et al. 2019)	*in-vitro and In-vivo SCID mice*	Cell viability, cell cycle and CCD2 activation	T47D BT-474 SKBR3 MDA-MB231 MDA-MB453	Human	breast cancer	27MHz (carrier) amplitude modulated envelope 500 Hz to 22Khz	3 h/day	30 and 400 mW/kg	None	Cell viability assay by Thymidine incorporation, flow cytometry, western blot, RT-PCR for RNA a cDNA, gene set enrichment analysis, knockdown and overexpression . Intracranial injection for *in-vivo* experiments	sXc-ELF and sXv27, IT'IS Corporation		↓ cell proliferation, ↓brain metastasis, ↑calcium influx, ↑p38 pathway, ↓HMGA2 expression
(Siasi and Moniri 2021)	*in-vitro*	Cell viability and network pathway	AGS-C10071	Human	gastric cancer cell	10	18 h	0.25-2.5	None	Cell viability assay by MTT, RNA and cDNA analysis by PCR and network analysis	vertical EMF composed of 1200- windings in 4 coil systems (with 1mm diameter on PVC with 30x12 cm), and placed inside a metal chamber.		↓cell viability, ↑ miR-29 and miR-21,
(Solek, Majchrowicz et al. 2017)	*in-vitro*	oxidative stress, cell viability and cell cycle analysis	GC-1 GC-2	Mouse	spermatogenic cells	50 (2/150 and pulsed)	2 hr (assessed 48 hr later)	2.5 - 8	None	cell metabolism by MTT, cell viability by Fluo Cell Double Staining Kit, Western blot with anti-β-actin , antip21, anti-p53, anti-NF-κB, anti-Bcl-2 and anti-active caspase 3	designed and constructed in Poland (Bielsko-Biala)	↑ROS (GC-1), ↑NO	↓cell proliferation (CEMF) ◆cell proliferation (PEMF),↑NFkB, ↑G2/M (arrest),↓caspase3 and Bcl-2

(Solek, Majchrowicz et al. 2018)	*in-vitro*	oxidative stress, cell viability and cell cycle analysis	GC-1	Mouse	spermatogenic cells	50 (50 Hz pulsed)	2 hr (assessed 48 hr later)	2,5	Aloe arborescens juice	cell metabolism by MTT, cell viability by Fluo Cell Double Staining Kit, Western blot with anti-β-actin , antip21, anti-p53, anti-NF-κB, anti-Bcl-2 and anti-active caspase 4	designed and constructed in Poland (Bielsko-Biala)	↑ROS (GC-1), ↓NO	↑P53, ↑caspase3 and p21, ↑NFkB ◆cell proliferation
(Song, Im et al. 2018)	*in-vitro*	Oxidative stress and cell viability	HeLa IMR-90	Human	cervix carcinoma lung fibroblast	60	10, 30, 60 min repeated or continous for days	1, 3, 6, 10	None	cell proliferation by MTT, ROS by carboxy-H2DCFDA, flow cytometry, Western blot with anti-sera against β-actin, histone H3, phospho-histone H3, CDK4, p-Erk1/2, Erk1/2, p-Akt, Akt and γ-H2AX	Each coil is 0.4 mm in diameter and has 1,000 turns. Magnetic flux density controlled proportional to the coil current.	↓ROS	◆cell proliferation, ◆DNA damage,↑cellular proliferation, ↑Akt/Erk signaling
(Su, Yimaer et al. 2017)	*in-vitro and In-vivo male (Sprague–Dawley rats)*	Cell viability and morphology changes	U251 A172	Human Rat	Glioblastoma and glia cells (rat)	50	1, 6 or 24h	2	None	Cell cycle analysis by flow cytometry, cell viability by CCK-8, TNF-α, IL-6 and IL-1β by ELISA, phagocytosis assay, morphological analysis	sXc-ELF, IT'IS Corporation	↑ROS (older neurons) ◆ROS (younger neurons) ↑Nox2 (8 hr/day)	◆γH2AX, ◆cell proliferation, ◆TNF-α, IL-6 and IL-1β, ◆TT

(Sun, Chen et al. 2018)	*in-vitro*	Oxidative stress and EGFR expression	FL	Human	amniotic epithelial cells	50	5, 15, 30 min	0,4	N-acetyl-L-cysteine pyrrolidine dithiocarbamate	ROS kit, Amplex Red Sphingomyelinase Assay Kit, cofocal microscopy	sXc-ELF, IT'IS Corporation	↑ROS	↑ EGFR clustering
(Villarini, Gambelunghe et al. 2017)	*in-vitro*	Oxidative stress and DNA damage	SH-SY5Y SK-NB-E-2	Human	Brain/neuroblastoma	50	1 hr (continous) 5 hr (intermittent, 15/15 min)	0.01, 0.1, 1	$AlCl_3$	acridine orange (AO) and diaminophenylindole (DAPI) for cell viabilit, cellular redox status by reduced glutathione (GSH) and glutathione disulfide (GSSG) contenty, DNA damage by Comet assay	pair of parallel coils (16.5 cm external diameter and 12 cm distance between the coils) arranged horizontally in a Helmholtz-like configuration	◆GSH	◆HSP70, ◆DNA damage
(Wang, Chen et al. 2021)	*in-vitro*	Cell viability, apoptosis and calcium influx	MCF-7 MDA-MB-231	Human	breast cancer normal breast epithelium	7.83, 23.49, 39.15	12, 24, and 48 h	0.5-1.0		Cell viability assay by MTT, flow cytometry for apoptosis, calcium inflow by fluorescence	ELF-EMF device consited, a DC power supply, a timer and a coil under a 96-well plate. Cells seeded in the 96-well culture plate were exposed to ELF-EMF. The coil was placed at the bottom of the culture plate.		↓cell viability in cancer, ◆cell viability in normal cells, ↑apoptosis in cancer, ↑calcium influx in cancer

(Wang, Liu et al. 2019)	In-vitro and In-vivo male Sprague–Dawley rats	Oxidative stress and DNA damage	AC16	Human Rat	cardiomyocyte	50	1 hr (continous) 1.25 hr (intermittent, 15/15 min)	0,1	None	cell cycle analysis, western blot, comet assays for DNA damage, ROS assay kit, GSH and GSSG Assay Kit	pair of parallel coils (200 cm×70 cm×200 cm, L×W×H), was built by Yite Electric (Wuhan, China)	◆ROS, ◆GSH	◆DNA damage, ◆P53, ◆HSP70
(Wang, Zhang et al. 2018)	*in-vitro*	Oxidative stress and ATP production	HCT116 HEK-293T RPE-1 Gist-T1 & others	Human	colon carcinoma kidney retina epithelial gastrointestinal stromal	50 Hz (static, 120)	2 hr (4, 6 hr)	6	None	ATP Determination Kit, JC-1 dye for matrix metalloproteinase, 2′,7′-dichlorofluorescin diacetate for ROS,	two 126 mm × 85 mm × 22 mm 12-well plates and a 118 mm × 85 mm × 78 mm plastic box on the top center of a 60 mm × 50 mm × 35 mm neodymium N38 permanent magnet (Jiangsu Zhongxin magnetoelectricity, Dafeng, China)	↑ROS (HCT116) ↓ROS (RPE-1) ◆ROS (Gist-T1, HEK-293T)	altered ATP level dependent on cell lines

(Wójcik-Piotrowicz, Kaszuba-Zwoińska et al. 2023)	*in-vitro*	Cell viability and cell cycle	MM6 U937	Human	leukocytic cell	35 - 50	12, 24, and 48 h	6, 13		Cell viability assay and apoptosis by flow cytometry, ROS by cytochrome c reduction and Western blot	AC coil or DC Helmholtz coils arranged in series with an externally placed signal generator with signal amplifier. The coils were placed to preserve the geometry and spatial distribution of MF and air cooling	↑ROS	↓cell viability, ↑CaN and HSP70, ↑calcium influx
(Wust, Veltsista et al. 2022)	*in-vitro and In-vivo female NMRI nu/nu mice*	Cell viability and apoptosis	HT29 SW480 LoVo SW620 HCT116	Human	Colon carcinoma	1, 10, 100 Hz or 1 kHz	1 h	1 , 10 (W)	None	Cell cycle analysis by flow cytometry, cell viability by CCK-8, TNF-α, IL-6 and IL-1β by ELISA, phagocytosis assay, morphological analysis	LabEHY-200 (Oncotherm Kft Budapest, Hungary)		↑apoptosis, ↓cell proliferation,
(Xu, Wang et al. 2020)	*in-vitro*	Oxidative stress and cell viability	MCF-7 ZR-75-1 T47D	Human	breast cancer	50 (125, 200, 275)	6, 12, 24, 36 hr	1	N-acetyl-L-cysteine	cell viability by MTT, apoptpsis by Annexin V-FITC Apoptosis Detection Kit, DCFH-DA for ROS, cell cycle analysis and Western blot	two coils were designed with 64 turns, while the middle one had 50 turns with the same radius of 130 mm	↑ROS (optimal at 200 Hz)	↓Cell proliferation (all frequencies), ↑Apoptosis (optimal at 200 Hz)

(Zeng, Shen et al. 2017)	*in-vitro*	Oxidative stress and cell viability	Cell extration from rats	Rat	hippcampal neurons	50	0.5, 8, 24 hr<br>0.5, 8 hr/day repeated	2	None	CCK8 Cell Count Kit, DCFHDA for ROS, fluorecence for anti-cH2AX, TUNEL analysis	two identical coil systems placed in a 25925925 cm3 l-metal box with a thickness of 1.3 mm	↑ROS (older neurons)<br>◆ROS (younger neurons)<br>↑Nox2 (8 hr/day)	↓Cell proliferation, ◆DNA damage, ◆apoptosis
(Zimmerman, Pennison et al. 2012)	*in-vitro*	Cell viability	HepG2<br>Huh7<br>MCF-7<br>THLE-2	Human	Hepatocellular carcinoma<br>Breast cancer	27MHz (carrier) amplitude modulated envelope 500 Hz to 22Khz	1h/day, 3h/day, 6h/day for 7 days, 3h/day for 3 days	0.4 (Wk g^{-1})	None	Cell viability by luminescence and thymidine incorporation, PCR and RNA seq, kariotype analysis, cofocal microscopy	sXc-ELF, IT'IS Corporation		↓Cell proliferation in cancer, ◆Cell proliferation in normal cells, ↑ expression of PLP2 and XCL2, disruption of mitotic spindle

In-vitro experiments

Reference	Species	Animal number	Strain	Organ	Freq. (Hz)	Regimen	Dose (µT)	Metodology	Exposure system	Oxidative Stress Markers	Tissue Impact
(Akdag, Dasdag et al. 2010)	Rat	30	adult male Sprague–Dawley rats	Brain	50	2 hr/day, 10 months	0,231	immunohistochemical for caspase-3. CAT, MDA, TAC TOS, OSI, MPO levels in tissue samples	500 µT ELF-MF for 2 h/day (7 days in a week) during 10 months in a Plexiglas cage. Helmholtz coils of 25 cm in diameter in a Faraday cage (130×65×80 cm)	↓SOD, ↑MDA, ↑TOS	↑apopotis
(Bediz, Baltaci et al. 2006)	Rat	24	adult male Sprague–Dawley rats	Brain	50	5 minutes / 2nd. Day, 6 months	100	MDA and Zinc plama levels by spectrophotometer TBARS, GSH in brain samples	ELF-MF for 5 min/day for 6 months in the cage, a single isolated copper wire	↑TBARS, ↑MDA	↓GSH
(Cho, Nam et al. 2012)	Rat	20	adult male Sprague–Dawley rats	Brain	50	5 days	2000	NOx in brain samples, cGMP by ELISA, anti-rat NOS-1 (nNOS) antibodies, morphological assessments of nuclear and mitochondrial damage by electrom microscopy	one pair of Helmholtz coils with windings embedded in an open wooden rectangular frame (140x85x 70 cm3). ELF-MF for 5 days in the cage	↑NO, ↑NOS	◆TT
(Chu, Lee et al. 2011)	Mice	20	male Balb/C mice	Brain	40	3 hrs	2300	Brain sample to MDA by TBA, ROS by 2,3-DHBA, SOD by fluorecence, GPx and GSH by chromatography, MDA levels.	ELF-MF for 3 hours in the mouse cage	↑LP (30 min.), ◆LP (60 min), ◆ROS ↑NOS, ↑MDA	◆GSH
(Deng, Zhang et al. 2013)	Mice	60	male Kumming	Brain	50	4 hrs/day, 8 weeks	2000	SOD and MDA levels in brain samples and plasma, Western blot.	ELF-MF for 4 h daily 6 days per week for 8 weeks. Helmholtz coil composed of copper wire	↑MDA,↓SOD	↑TT, ↓pyramidal cells

(Erdal, Gürgül et al. 2008)	Rat	32	adult male and female Wistar-albino	Brain	50	4 hrs/day, 45 days	1000	MDA levels in brain sample,3-NT Chemical assay,	70x65x65cm3 Faraday cage. Helmholtz coil composed of copper wire of 42.75 cm and 21.37 cm diameters	◆MDA,↑3-NT	
(Falone, Mirabilio et al. 2008)	Rat	40	adult female Sprague–Dawley rats	Brain	50	10 days	100	SOD, GPx, Catalase, GR, GST and RNA gene expression analysis in brain samples, and Western blot.	pair of Helmholtz coils (r = 630 mm, distance between coils = 700 mm) (Oersted Technology Corp., Oregon, USA). EMT exposed to the 50 Hz magnetic field of 0.1 mT for 10 days continuously	↑GPX1, GR, GST, SOD2 (aged rats), ◆GPX1, CAT, GST, SOD1 (young rats)	
(Gholami, Riazi et al. 2019)	rat	20	male Wistar rats	Sperm and brain	50	24 hrs/day, 85 days	500	turbidity, fluorecence spectroscopy	Helmholtz coil (The radius of each coil 35mm, 70mm high, copper wire, 1000 turns/m, the diameter of the wire in each coil 1.7mm, self-inductance L=3mH, ohmic resistance = 3 Ω) as electromagnetic field generator	↑microtube polimerization, ↑tubulin polymerization, ↑disruption tubuline structure	
(Jelenković, Janać et al. 2006)	Rat	12	male Wistar-albino	Brain	50	7 days	500	SOD, ROS, LP, NO, MDA levels	solenoid type electromagnet with a regular laminated transformer core and pole dimensions 9.5 cm x 9.5 cm. Exposed to ELF-MF during 7 days	↑ROS, ↑SOD, ↑NO, ↑MDA	

(Lei, Liang et al. 2018)	mice	33	female BALB/c mice	Liver	15 (pulsed burst)	8 h/day, 7 days/ week, for 8 weeks	160	ROS, SOD, CAT, GSHPx and TG.	custom-built, whichwas consisted of four parts: Labviewsoftware,multifunction data acquisition device (NI USB-6211), power amplifier (XP9900S, Huamei, China) and Helmholtz coils	↓TG, ↓CaMKKβ,↑AMPK ↑CaMKKβ/AMPK/SREBP-1c pathway	
(Manikonda, Rajendra et al. 2014)	Rat	18	male Wistar-albino	Brain	50	90 days	50, 100, 500	ROS by DCFDA, TBARS, glutathione, SOD, GPx in brain samples	wooden bobbins (0.5 m × 0.5 m × 0.5 m) with two sets of horizontal coils (each set of 25 turns), separated by a distance of 5 mm. The coils were made of 22 gauge enameled copper wire	↑ROS, ↑LP, ↑TBARS,↓GSH and GSSG, ↑GPx	◆TT
(Martínez-Sámano, Torres-Durán et al. 2010)	Rat	32	male Wistar	Plasma, liver, kidney, heart	60	2 hrs	2500	SOD, Catalase, GSH, NO, total lipid, TBARS analysis from tissue samples and plasma	home cages of 47 x 21 x 25 cm. a pair of Helmholtz coils (30 cm internal diameter) composed of 18 gauge copper wire in parallel w 350 turns	↓GSH (heart, liver), ↓SOD (plasma), ↓CAT, ↓NO, ↑TBARS	effects increased with movement restrainment
(Tekutskaya, Ryabova et al. 2022)	Rat	60	male Wistar	Blood	3 to 60	2 hrs/day, 5 weeks	200	ROS, SOD, CAT, GSHPx and TG.	sinusoidal signal of precision form, inductor coil with 1200 turns placed in a shielded chamber. EMF created by the coil was 30 mT, coil resistivity was 320 Ω, and the voltage across the coil was 14 V. EMF exposure for 15 min in the mode of continuous signal at selected frequency.	↑ROS, ↑SOD	

(Yokus, Cakir et al. 2005)	Rat	48	female Wistar	Blood	50	3 hrs/day, 50 and 100 days	970	DNA analysis, 8OHdG levels and TBARS in plasma and blood	two pairs of Helmholtz coils of 25 cm in diameter. w 225 turns of insulated copper wire with a diameter of 1.0 mm. Coils placed vertically and horizontally as facing one another. The distance between coils was 25 cm. Groups of EMF exposure for 50 days or 100 days, 3 h a day, or sham	↑8-OHdG, ↑TBARS	
(Zhai, Zhang et al. 2023)	mice	24	male C57BL/6 mice	Liver	15 (pulsed burst)	2 hrs/day, 5 weeks	160	ROS, SOD, CAT, GSHPx and TG.	custom-built, whichwas consisted of four parts: Labviewsoftware,multifunction data acquisition device (NI USB-6211), power amplifier (XP9900S, Huamei, China) and Helmholtz coils	↓TG, ↓CaMKKβ,↑AMPK ↑CaMKKβ/AMPK/SREBP-1c pathway	

References for supplement information

Akan, Z., B. Aksu, A. Tulunay, S. Bilsel and A. Inhan-Garip (2010). "Extremely low-frequency electromagnetic fields affect the immune response of monocyte-derived macrophages to pathogens." Bioelectromagnetics **31**(8): 603-612.

Akdag, M. Z., S. Dasdag, E. Ulukaya, A. K. Uzunlar, M. A. Kurt and A. Taşkin (2010). "Effects of extremely low-frequency magnetic field on caspase activities and oxidative stress values in rat brain." Biol Trace Elem Res **138**(1-3): 238-249.

Ayşe, I. G., A. Zafer, O. Sule, I. T. Işil and T. Kalkan (2010). "Differentiation of K562 cells under ELF-EMF applied at different time courses." Electromagn Biol Med **29**(3): 122-130.

Bediz, C. S., A. K. Baltaci, R. Mogulkoc, Ouml and E. ztekin (2006). "Zinc Supplementation Ameliorates Electromagnetic Field-Induced Lipid Peroxidation in the Rat Brain." The Tohoku Journal of Experimental Medicine **208**(2): 133-140.

Benassi, B., G. Filomeni, C. Montagna, C. Merla, V. Lopresto, R. Pinto, C. Marino and C. Consales (2016). "Extremely Low Frequency Magnetic Field (ELF-MF) Exposure Sensitizes SH-SY5Y Cells to the Pro-Parkinson's Disease Toxin MPP(.)." Mol Neurobiol **53**(6): 4247-4260.
Bergandi, L., G. Grisolia, R. Granata, I. Gesmundo, A. Ponzetto, E. Paolucci, R. Borchiellini, E. Ghigo and F. Silvagno (2019). "The extremely low frequency electromagnetic stimulation selective for cancer cells elicits growth arrest through a metabolic shift." Biochimica et Biophysica Acta (BBA) - Molecular Cell Research **1866**.
Brisdelli, F., F. Bennato, A. Bozzi, B. Cinque, F. Mancini and R. Iorio (2014). "ELF-MF attenuates quercetin-induced apoptosis in K562 cells through modulating the expression of Bcl-2 family proteins." Mol Cell Biochem **397**(1-2): 33-43.
Buckner, C. A., A. L. Buckner, S. A. Koren, M. A. Persinger and R. M. Lafrenie (2015). "Inhibition of cancer cell growth by exposure to a specific time-varying electromagnetic field involves T-type calcium channels." PLoS One **10**(4): e0124136.
Bułdak, R. J., R. Polaniak, L. Bułdak, K. Zwirska-Korczala, M. Skonieczna, A. Monsiol, M. Kukla, A. Duława-Bułdak and E. Birkner (2012). "Short-term exposure to 50 Hz ELF-EMF alters the cisplatin-induced oxidative response in AT478 murine squamous cell carcinoma cells." Bioelectromagnetics **33**(8): 641-651.
Calcabrini, C., U. Mancini, R. De Bellis, A. R. Diaz, M. Martinelli, L. Cucchiarini, P. Sestili, V. Stocchi and L. Potenza (2017). "Effect of extremely low-frequency electromagnetic fields on antioxidant activity in the human keratinocyte cell line NCTC 2544." Biotechnol Appl Biochem **64**(3): 415-422.
Chen, Y., L. Hong, Y. Zeng, Y. Shen and Q. Zeng (2014). "Power frequency magnetic fields induced reactive oxygen species-related autophagy in mouse embryonic fibroblasts." Int J Biochem Cell Biol **57**: 108-114.
Cho, S. I., Y. S. Nam, L. Y. Chu, J. H. Lee, J. S. Bang, H. R. Kim, H. C. Kim, Y. J. Lee, H. D. Kim, J. D. Sul, D. Kim, Y. H. Chung and J. H. Jeong (2012). "Extremely low-frequency magnetic fields modulate nitric oxide signaling in rat brain." Bioelectromagnetics **33**(7): 568-574.
Chu, L. Y., J. H. Lee, Y. S. Nam, Y. J. Lee, W. H. Park, B. C. Lee, D. Kim, Y. H. Chung and J. H. Jeong (2011). "Extremely low frequency magnetic field induces oxidative stress in mouse cerebellum." Gen Physiol Biophys **30**(4): 415-421.
Consales, C., C. Cirotti, G. Filomeni, M. Panatta, A. Butera, C. Merla, V. Lopresto, R. Pinto, C. Marino and B. Benassi (2018). "Fifty-Hertz Magnetic Field Affects the Epigenetic Modulation of the miR-34b/c in Neuronal Cells." Mol Neurobiol **55**(7): 5698-5714.
Consales, C., M. Panatta, A. Butera, G. Filomeni, C. Merla, M. T. Carrì, C. Marino and B. Benassi (2019). "50-Hz magnetic field impairs the expression of iron-related genes in the in vitro SOD1(G93A) model of amyotrophic lateral sclerosis." Int J Radiat Biol **95**(3): 368-377.
Costantini, E., B. Sinjari, C. D'Angelo, G. Murmura, M. Reale and S. Caputi (2019). "Human Gingival Fibroblasts Exposed to Extremely Low-Frequency Electromagnetic Fields: In Vitro Model of Wound-Healing Improvement." Int J Mol Sci **20**(9).
de Groot, M. W., M. D. Kock and R. H. Westerink (2014). "Assessment of the neurotoxic potential of exposure to 50Hz extremely low frequency electromagnetic fields (ELF-EMF) in naïve and chemically stressed PC12 cells." Neurotoxicology **44**: 358-364.

Deng, Y., Y. Zhang, S. Jia, J. Liu, Y. Liu, W. Xu and L. Liu (2013). "Effects of Aluminum and Extremely Low Frequency Electromagnetic Radiation on Oxidative Stress and Memory in Brain of Mice." Biological Trace Element Research **156**(1): 243-252.
Destefanis, M., M. Viano, C. Leo, G. Gervino, A. Ponzetto and F. Silvagno (2015). "Extremely low frequency electromagnetic fields affect proliferation and mitochondrial activity of human cancer cell lines." Int J Radiat Biol **91**(12): 964-972.
Duan, W., C. Liu, L. Zhang, M. He, S. Xu, C. Chen, H. Pi, P. Gao, Y. Zhang, M. Zhong, Z. Yu and Z. Zhou (2015). "Comparison of the genotoxic effects induced by 50 Hz extremely low-frequency electromagnetic fields and 1800 MHz radiofrequency electromagnetic fields in GC-2 cells." Radiat Res **183**(3): 305-314.
Duong, C. N. and J. Y. Kim (2016). "Exposure to electromagnetic field attenuates oxygen-glucose deprivation-induced microglial cell death by reducing intracellular Ca(2+) and ROS." Int J Radiat Biol **92**(4): 195-201.
Erdal, N., S. Gürgül, L. Tamer and L. Ayaz (2008). "Effects of long-term exposure of extremely low frequency magnetic field on oxidative/nitrosative stress in rat liver." J Radiat Res **49**(2): 181-187.
Errico Provenzano, A., S. Amatori, M. G. Nasoni, G. Persico, S. Russo, A. R. Mastrogiacomo, A. Gambarara and M. Fanelli (2018). "Effects of Fifty-Hertz Electromagnetic Fields on Granulocytic Differentiation of ATRA-Treated Acute Promyelocytic Leukemia NB4 Cells." Cell Physiol Biochem **46**(1): 389-400.
Falone, S., A. Mirabilio, M. C. Carbone, V. Zimmitti, S. Di Loreto, M. A. Mariggiò, R. Mancinelli, C. Di Ilio and F. Amicarelli (2008). "Chronic exposure to 50Hz magnetic fields causes a significant weakening of antioxidant defence systems in aged rat brain." Int J Biochem Cell Biol **40**(12): 2762-2770.
Feng, B., A. Dai, L. Chen, L. Qiu, Y. Fu and W. Sun (2016). "NADPH oxidase-produced superoxide mediated a 50-Hz magnetic field-induced epidermal growth factor receptor clustering." Int J Radiat Biol **92**(10): 596-602.
Feng, B., L. Qiu, C. Ye, L. Chen, Y. Fu and W. Sun (2016). "Exposure to a 50-Hz magnetic field induced mitochondrial permeability transition through the ROS/GSK-3β signaling pathway." Int J Radiat Biol **92**(3): 148-155.
Feng, B., C. Ye, L. Qiu, L. Chen, Y. Fu and W. Sun (2016). "Mitochondrial ROS Release and Subsequent Akt Activation Potentially Mediated the Anti-Apoptotic Effect of a 50-Hz Magnetic Field on FL Cells." Cell Physiol Biochem **38**(6): 2489-2499.
Frahm, J., M.-O. Mattsson and M. Simkó (2010). "Exposure to ELF magnetic fields modulate redox related protein expression in mouse macrophages." Toxicology Letters **192**(3): 330-336.
Gholami, D., G. Riazi, R. Fathi, M. Sharafi and A. Shahverdi (2019). "Comparison of polymerization and structural behavior of microtubules in rat brain and sperm affected by the extremely low-frequency electromagnetic field." BMC Mol Cell Biol **20**(1): 41.
Hong, M. N., N. K. Han, H. C. Lee, Y. K. Ko, S. G. Chi, Y. S. Lee, Y. M. Gimm, S. H. Myung and J. S. Lee (2012). "Extremely low frequency magnetic fields do not elicit oxidative stress in MCF10A cells." J Radiat Res **53**(1): 79-86.
Höytö, A., M. Herrala, J. Luukkonen, J. Juutilainen and J. Naarala (2017). "Cellular detection of 50 Hz magnetic fields and weak blue light: effects on superoxide levels and genotoxicity." Int J Radiat Biol **93**(6): 646-652.

Huang, C. Y., C. W. Chang, C. R. Chen, C. Y. Chuang, C. S. Chiang, W. Y. Shu, T. C. Fan and I. C. Hsu (2014). "Extremely low-frequency electromagnetic fields cause G1 phase arrest through the activation of the ATM-Chk2-p21 pathway." PLoS One **9**(8): e104732.
Jelenković, A., B. Janać, V. Pesić, D. M. Jovanović, I. Vasiljević and Z. Prolić (2006). "Effects of extremely low-frequency magnetic field in the brain of rats." Brain Res Bull **68**(5): 355-360.
Jeong, W. Y., J. B. Kim, H. J. Kim and C. W. Kim (2017). "Extremely low-frequency electromagnetic field promotes astrocytic differentiation of human bone marrow mesenchymal stem cells by modulating SIRT1 expression." Biosci Biotechnol Biochem **81**(7): 1356-1362.
Jimenez, H., M. Wang, J. W. Zimmerman, M. J. Pennison, S. Sharma, T. Surratt, Z. X. Xu, I. Brezovich, D. Absher, R. M. Myers, B. DeYoung, D. L. Caudell, D. Chen, H. W. Lo, H. K. Lin, D. W. Godwin, M. Olivier, A. Ghanekar, K. Chen, L. D. Miller, Y. Gong, M. Capstick, R. B. D'Agostino, Jr., R. Munden, P. Merle, A. Barbault, A. W. Blackstock, H. L. Bonkovsky, G. Y. Yang, G. Jin, L. Liu, W. Zhang, K. Watabe, C. F. Blackman and B. C. Pasche (2019). "Tumour-specific amplitude-modulated radiofrequency electromagnetic fields induce differentiation of hepatocellular carcinoma via targeting Ca(v)3.2 T-type voltage-gated calcium channels and Ca(2+) influx." EBioMedicine **44**: 209-224.
Kesari, K. K., J. Juutilainen, J. Luukkonen and J. Naarala (2016). "Induction of micronuclei and superoxide production in neuroblastoma and glioma cell lines exposed to weak 50 Hz magnetic fields." J R Soc Interface **13**(114): 20150995.
Kesari, K. K., J. Luukkonen, J. Juutilainen and J. Naarala (2015). "Genomic instability induced by 50Hz magnetic fields is a dynamically evolving process not blocked by antioxidant treatment." Mutat Res Genet Toxicol Environ Mutagen **794**: 46-51.
Kim, S. J., Y. W. Jang, K. E. Hyung, D. K. Lee, K. H. Hyun, S. H. Jeong, K. H. Min, W. Kang, J. H. Jeong, S. Y. Park and K. W. Hwang (2017). "Extremely low-frequency electromagnetic field exposure enhances inflammatory response and inhibits effect of antioxidant in RAW 264.7 cells." Bioelectromagnetics **38**(5): 374-385.
Lazzarini, R., M. Eléxpuru-Zabaleta, F. Piva, M. Giulietti, G. Fulgenzi, M. F. Tartaglione, L. Zingaretti, A. Tagliabracci, M. Valentino, L. Santarelli and M. Bracci (2023). "Effects of extremely low-frequency magnetic fields on human MDA-MB-231 breast cancer cells: proteomic characterization." Ecotoxicol Environ Saf **253**: 114650.
Lei, T., Z. Liang, F. Li, C. Tang, K. Xie, P. Wang, X. Dong, S. Shan, M. Jiang, Q. Xu, E. Luo and G. Shen (2018). "Pulsed electromagnetic fields (PEMF) attenuate changes in vertebral bone mass, architecture and strength in ovariectomized mice." Bone **108**: 10-19.
Lekovic, M. H., N. E. Drekovic, N. D. Granica, E. H. Mahmutovic and N. Z. Djordjevic (2020). "Extremely low-frequency electromagnetic field induces a change in proliferative capacity and redox homeostasis of human lung fibroblast cell line MRC-5." Environ Sci Pollut Res Int **27**(31): 39466-39473.
Li, Y. and P. Heroux (2014). "Extra-low-frequency magnetic fields alter cancer cells through metabolic restriction." Electromagn Biol Med **33**(4): 264-275.

Liu, Y., W. B. Liu, K. J. Liu, L. Ao, J. Cao, J. L. Zhong and J. Y. Liu (2016). "Overexpression of miR-26b-5p regulates the cell cycle by targeting CCND2 in GC-2 cells under exposure to extremely low frequency electromagnetic fields." Cell Cycle **15**(3): 357-367.
Luukkonen, J., A. Liimatainen, J. Juutilainen and J. Naarala (2014). "Induction of genomic instability, oxidative processes, and mitochondrial activity by 50Hz magnetic fields in human SH-SY5Y neuroblastoma cells." Mutat Res **760**: 33-41.
Maiullari, S., A. Cicirelli, A. Picerno, F. Giannuzzi, L. Gesualdo, A. Notarnicola, F. Sallustio and B. Moretti (2023). "Pulsed Electromagnetic Fields Induce Skeletal Muscle Cell Repair by Sustaining the Expression of Proteins Involved in the Response to Cellular Damage and Oxidative Stress." Int J Mol Sci **24**(23): 16631.
Manikonda, P. K., P. Rajendra, D. Devendranath, B. Gunasekaran, Channakeshava, S. R. Aradhya, R. B. Sashidhar and C. Subramanyam (2014). "Extremely low frequency magnetic fields induce oxidative stress in rat brain." Gen Physiol Biophys **33**(1): 81-90.
Mannerling, A. C., M. Simkó, K. H. Mild and M. O. Mattsson (2010). "Effects of 50-Hz magnetic field exposure on superoxide radical anion formation and HSP70 induction in human K562 cells." Radiat Environ Biophys **49**(4): 731-741.
Martínez, M. A., A. Úbeda and M. Trillo (2021). "Role of NADPH oxidase in MAPK signaling activation by a 50 Hz magnetic field in human neuroblastoma cells." Electromagn Biol Med **40**(1): 103-116.
Martínez-Sámano, J., P. V. Torres-Durán, M. A. Juárez-Oropeza, D. Elías-Viñas and L. Verdugo-Díaz (2010). "Effects of acute electromagnetic field exposure and movement restraint on antioxidant system in liver, heart, kidney and plasma of Wistar rats: a preliminary report." Int J Radiat Biol **86**(12): 1088-1094.
Morabito, C., S. Guarnieri, G. Fanò and M. A. Mariggiò (2010). "Effects of acute and chronic low frequency electromagnetic field exposure on PC12 cells during neuronal differentiation." Cell Physiol Biochem **26**(6): 947-958.
Mousavi Maleki, N. S., M. Entezari, S. Abdi and N. Tekiyehmaroof (2022). "Electromagnetic Fields Change the Expression of Suppressor of Cytokine Signaling 3 (SOCS3) and Cathepsin L2 (CTSL2) Genes in Adenocarcinoma Gastric (AGS) Cell Line." Int J Cancer Manag **15**(3): e117270.
Nakayama, M., A. Nakamura, T. Hondou and H. Miyata (2016). "Evaluation of cell viability, DNA single-strand breaks, and nitric oxide production in LPS-stimulated macrophage RAW264 exposed to a 50-Hz magnetic field." Int J Radiat Biol **92**(10): 583-589.
Nezamtaheri, M. S., B. Goliaei, S. P. Shariatpanahi and A. M. Ansari (2022). "Differential biological responses of adherent and non-adherent (cancer and non-cancerous) cells to variable extremely low frequency magnetic fields." Scientific Reports **12**(1): 14225.
Park, J. E., Y. K. Seo, H. H. Yoon, C. W. Kim, J. K. Park and S. Jeon (2013). "Electromagnetic fields induce neural differentiation of human bone marrow derived mesenchymal stem cells via ROS mediated EGFR activation." Neurochem Int **62**(4): 418-424.
Patruno, A., P. Amerio, M. Pesce, G. Vianale, S. Di Luzio, A. Tulli, S. Franceschelli, A. Grilli, R. Muraro and M. Reale (2010). "Extremely low frequency electromagnetic fields modulate expression of inducible nitric oxide synthase, endothelial nitric oxide synthase and cyclooxygenase-2 in the human keratinocyte cell line HaCat: potential therapeutic effects in wound healing." Br J Dermatol **162**(2): 258-266.

Patruno, A., E. Costantini, A. Ferrone, M. Pesce, F. Diomede, O. Trubiani and M. Reale (2020). "Short ELF-EMF Exposure Targets SIRT1/Nrf2/HO-1 Signaling in THP-1 Cells." Int J Mol Sci **21**(19).
Patruno, A., M. Pesce, A. Marrone, L. Speranza, A. Grilli, M. A. De Lutiis, M. Felaco and M. Reale (2012). "Activity of matrix metallo proteinases (MMPs) and the tissue inhibitor of MMP (TIMP)-1 in electromagnetic field-exposed THP-1 cells." J Cell Physiol **227**(6): 2767-2774.
Patruno, A., S. Tabrez, M. Pesce, S. Shakil, M. A. Kamal and M. Reale (2015). "Effects of extremely low frequency electromagnetic field (ELF-EMF) on catalase, cytochrome P450 and nitric oxide synthase in erythro-leukemic cells." Life Sci **121**: 117-123.
Ramazi, S., M. Salimian, A. Allahverdi, S. Kianamiri and P. Abdolmaleki (2023). "Synergistic cytotoxic effects of an extremely low-frequency electromagnetic field with doxorubicin on MCF-7 cell line." Sci Rep **13**: 8844.
Reale, M., M. A. Kamal, A. Patruno, E. Costantini, C. D'Angelo, M. Pesce and N. H. Greig (2014). "Neuronal cellular responses to extremely low frequency electromagnetic field exposure: implications regarding oxidative stress and neurodegeneration." PLoS One **9**(8): e104973.
Schuermann, D. and M. Mevissen (2021). "Manmade Electromagnetic Fields and Oxidative Stress-Biological Effects and Consequences for Health." Int J Mol Sci **22**(7).
Sharma, S., S. Y. Wu, H. Jimenez, F. Xing, D. Zhu, Y. Liu, K. Wu, A. Tyagi, D. Zhao, H. W. Lo, L. Metheny-Barlow, P. Sun, J. D. Bourland, M. D. Chan, A. Thomas, A. Barbault, R. B. D'Agostino, C. T. Whitlow, V. Kirchner, C. Blackman, B. Pasche and K. Watabe (2019). "Ca2+ and CACNA1H mediate targeted suppression of breast cancer brain metastasis by AM RF EMF." EBioMedicine **44**: 194-208.
Siasi, E. and E. Moniri (2021). "The effect of extremely low frequency electromagnetic fields following on upregulation of miR-21 and miR-29 in gastric cancer cell line." Gastroenterol Hepatol Bed Bench **14**(1): 67-76.
Solek, P., L. Majchrowicz, D. Bloniarz, E. Krotoszynska and M. Koziorowski (2017). "Pulsed or continuous electromagnetic field induce p53/p21-mediated apoptotic signaling pathway in mouse spermatogenic cells in vitro and thus may affect male fertility." Toxicology **382**: 84-92.
Solek, P., L. Majchrowicz and M. Koziorowski (2018). "Aloe arborescens juice prevents EMF-induced oxidative stress and thus protects from pathophysiology in the male reproductive system in vitro." Environ Res **166**: 141-149.
Song, K., S. H. Im, Y. J. Yoon, H. M. Kim, H. J. Lee and G. S. Park (2018). "A 60 Hz uniform electromagnetic field promotes human cell proliferation by decreasing intracellular reactive oxygen species levels." PLoS One **13**(7): e0199753.
Su, L., A. Yimaer, X. Wei, Z. Xu and G. Chen (2017). "The effects of 50 Hz magnetic field exposure on DNA damage and cellular functions in various neurogenic cells." J Radiat Res **58**(4): 474-486.
Sun, L., L. Chen, L. Bai, Y. Xia, X. Yang, W. Jiang and W. Sun (2018). "Reactive oxygen species mediates 50-Hz magnetic field-induced EGF receptor clustering via acid sphingomyelinase activation." International Journal of Radiation Biology **94**(7): 678-684.
Tekutskaya, E. E., I. S. Ryabova, S. V. Kozin, K. A. Popov and V. V. Malyshko (2022). "Changes in Free Radical Processes under the Influence of Low-Frequency Electromagnetic Field in Rats." Bull Exp Biol Med **172**(5): 566-569.

Villarini, M., A. Gambelunghe, D. Giustarini, M. V. Ambrosini, C. Fatigoni, R. Rossi, L. Dominici, S. Levorato, G. Muzi, D. Piobbico and G. Mariucci (2017). "No evidence of DNA damage by co-exposure to extremely low frequency magnetic fields and aluminum on neuroblastoma cell lines." Mutat Res Genet Toxicol Environ Mutagen **823**: 11-21.
Wang, D., L. Zhang, G. Shao, S. Yang, S. Tao, K. Fang and X. Zhang (2018). "6-mT 0-120-Hz magnetic fields differentially affect cellular ATP levels." Environ Sci Pollut Res Int **25**(28): 28237-28247.
Wang, M. H., K. W. Chen, D. X. Ni, H. J. Fang, L. S. Jang and C. H. Chen (2021). "Effect of extremely low frequency electromagnetic field parameters on the proliferation of human breast cancer." Electromagn Biol Med **40**(3): 384-392.
Wang, Y., X. Liu, Y. Zhang, B. Wan, J. Zhang, W. He, D. Hu, Y. Yang, J. Lai, M. He and C. Chen (2019). "Exposure to a 50 Hz magnetic field at 100 µT exerts no DNA damage in cardiomyocytes." Biol Open **8**(8).
Wójcik-Piotrowicz, K., J. Kaszuba-Zwoińska, P. Piszczek, B. Nowak, P. Guzdek, K. Gil and E. Rokita (2023). "Low-frequency electromagnetic fields influence the expression of calcium metabolism related proteins in leukocytic cell lines." Environ Toxicol Pharmacol **104**: 104320.
Wust, P., P. D. Veltsista, E. Oberacker, P. Yavvari, W. Walther, O. Bengtsson, A. Sterner-Kock, M. Weinhart, F. Heyd, P. Grabowski, S. Stintzing, W. Heinrich, U. Stein and P. Ghadjar (2022). "Radiofrequency Electromagnetic Fields Cause Non-Temperature-Induced Physical and Biological Effects in Cancer Cells." Cancers (Basel) **14**(21).
Xu, A., Q. Wang and T. Lin (2020). "Low-Frequency Magnetic Fields (LF-MFs) Inhibit Proliferation by Triggering Apoptosis and Altering Cell Cycle Distribution in Breast Cancer Cells." Int J Mol Sci **21**(8).
Yokus, B., D. U. Cakir, M. Z. Akdag, C. Sert and N. Mete (2005). "Oxidative DNA damage in rats exposed to extremely low frequency electro magnetic fields." Free Radic Res **39**(3): 317-323.
Zeng, Y., Y. Shen, L. Hong, Y. Chen, X. Shi, Q. Zeng and P. Yu (2017). "Effects of Single and Repeated Exposure to a 50-Hz 2-mT Electromagnetic Field on Primary Cultured Hippocampal Neurons." Neurosci Bull **33**(3): 299-306.
Zhai, M., C. Zhang, J. Cui, J. Liu, Y. Li, K. Xie, E. Luo and C. Tang (2023). "Electromagnetic fields ameliorate hepatic lipid accumulation and oxidative stress: potential role of CaMKKβ/AMPK/SREBP-1c and Nrf2 pathways." Biomed Eng Online **22**: 51.
Zimmerman, J. W., M. J. Pennison, I. Brezovich, N. Yi, C. T. Yang, R. Ramaker, D. Absher, R. M. Myers, N. Kuster, F. P. Costa, A. Barbault and B. Pasche (2012). "Cancer cell proliferation is inhibited by specific modulation frequencies." Br J Cancer **106**(2): 307-313.
